# Supplementary figures and images for: Highly multiplexed selection of RNA aptamers against a small molecule library
Source: PLoS One. 2022 Sep 15;17(9):e0273381. doi: 10.1371/journal.pone.0273381 (PMC9477273; doi:10.1371/journal.pone.0273381)

a

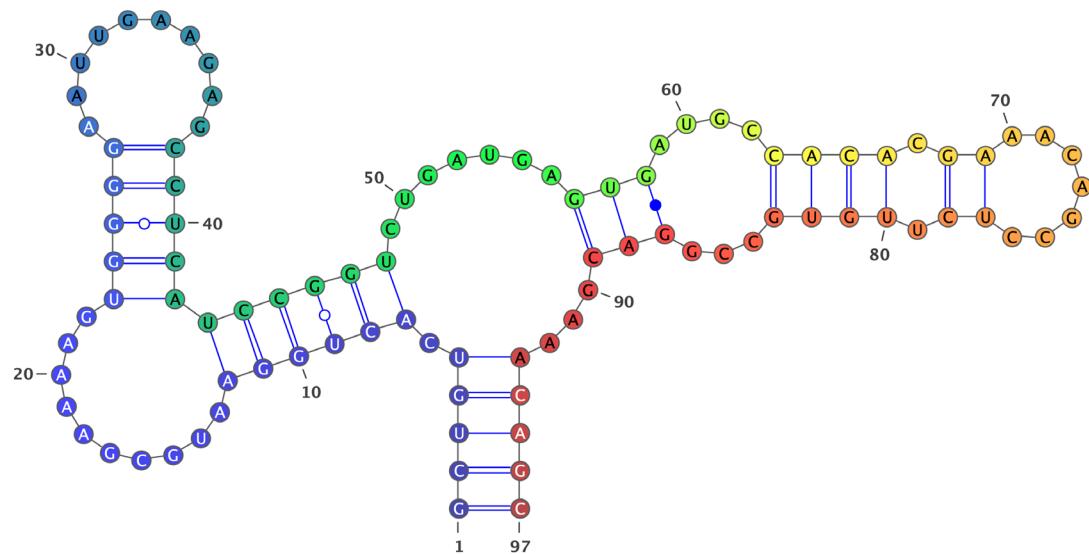

b

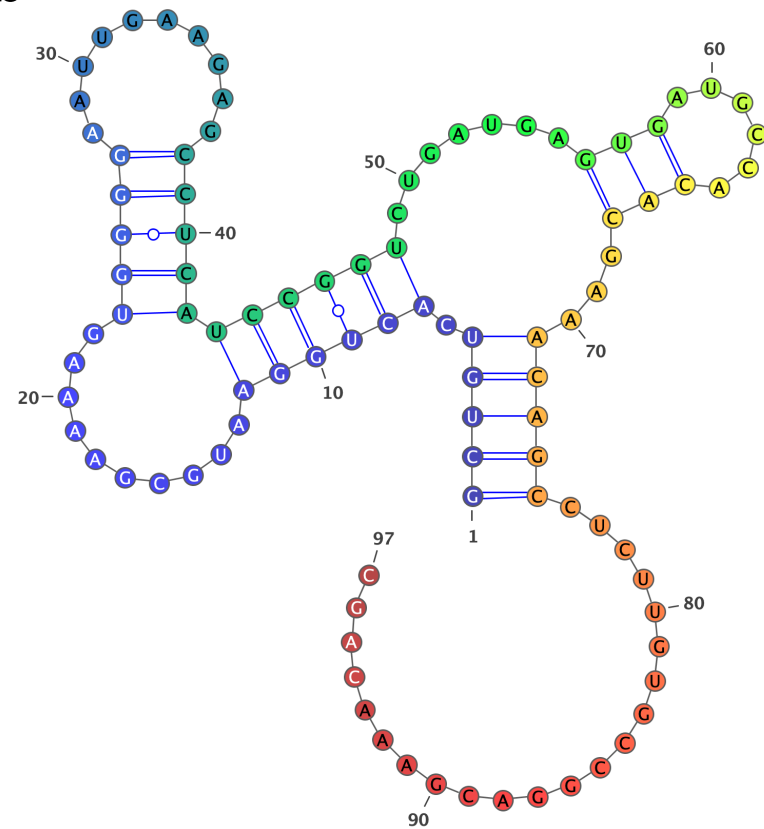

Supplement: S1 Fig — The sequence above is representative of several sequences that were enriched early in the selection and contain a structure that appears to have two stable secondary structures. (a) secondary structure in which all the nucleotides are involved in forming the ribozyme; (b) an alternative secondary structure which leaves the 5’ end free to anneal to the reverse transcription primer without disrupting the ribozyme structure. (PDF) [file pone.0273381.s001.pdf]

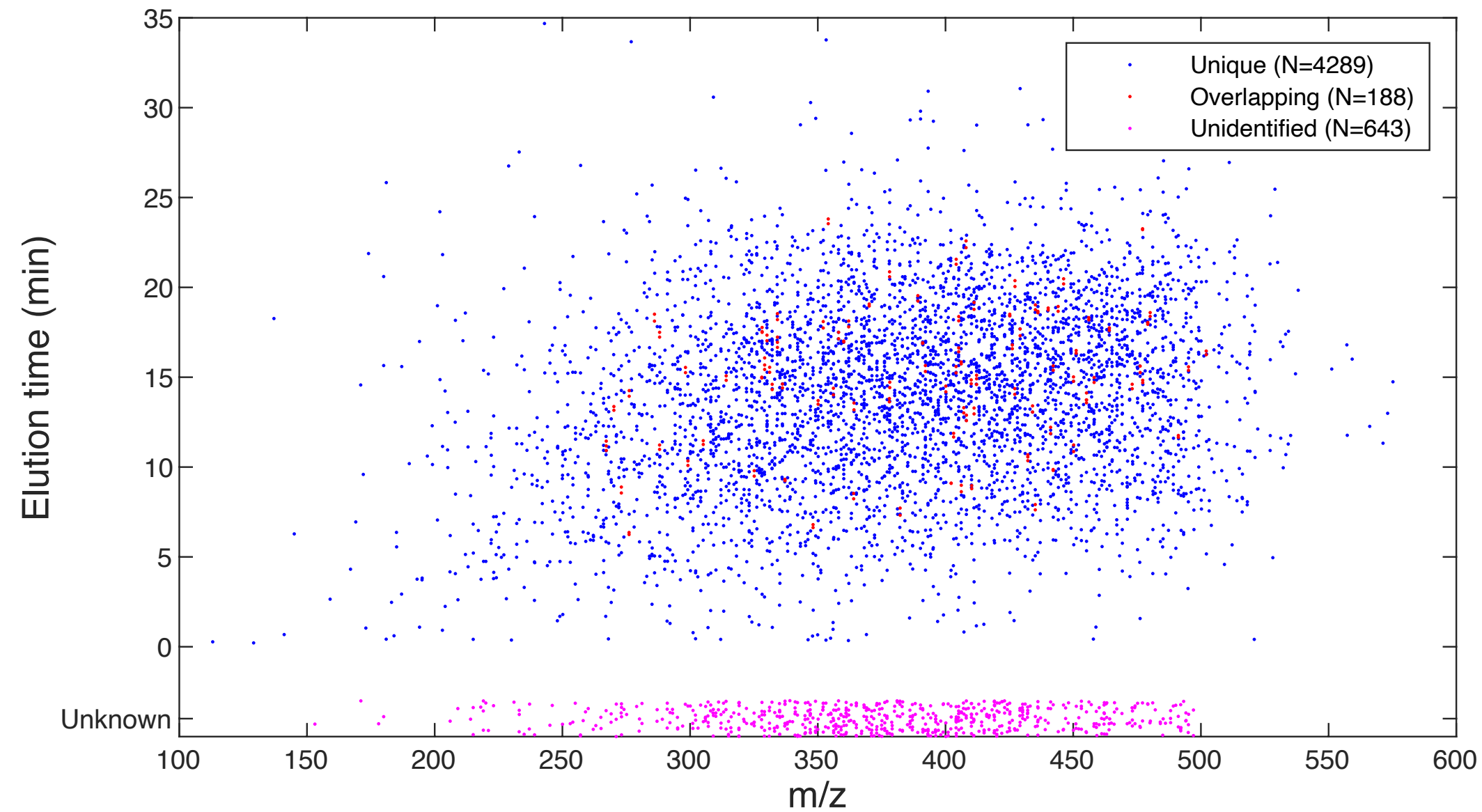

Supplement: S2 Fig — The elution time and m/z of the largest ion count peak matching expected adducts are shown for each of the compounds that occur in at least 4 out of 5 expected mixtures. Blue points indicate unambiguous assignments, red points are for compounds that overlap in elution time and m/z with at least one other compound, and magenta points show compounds that were not assigned an elution time. Data plotted here is contained in S1 Table. (PDF) [file pone.0273381.s002.pdf]

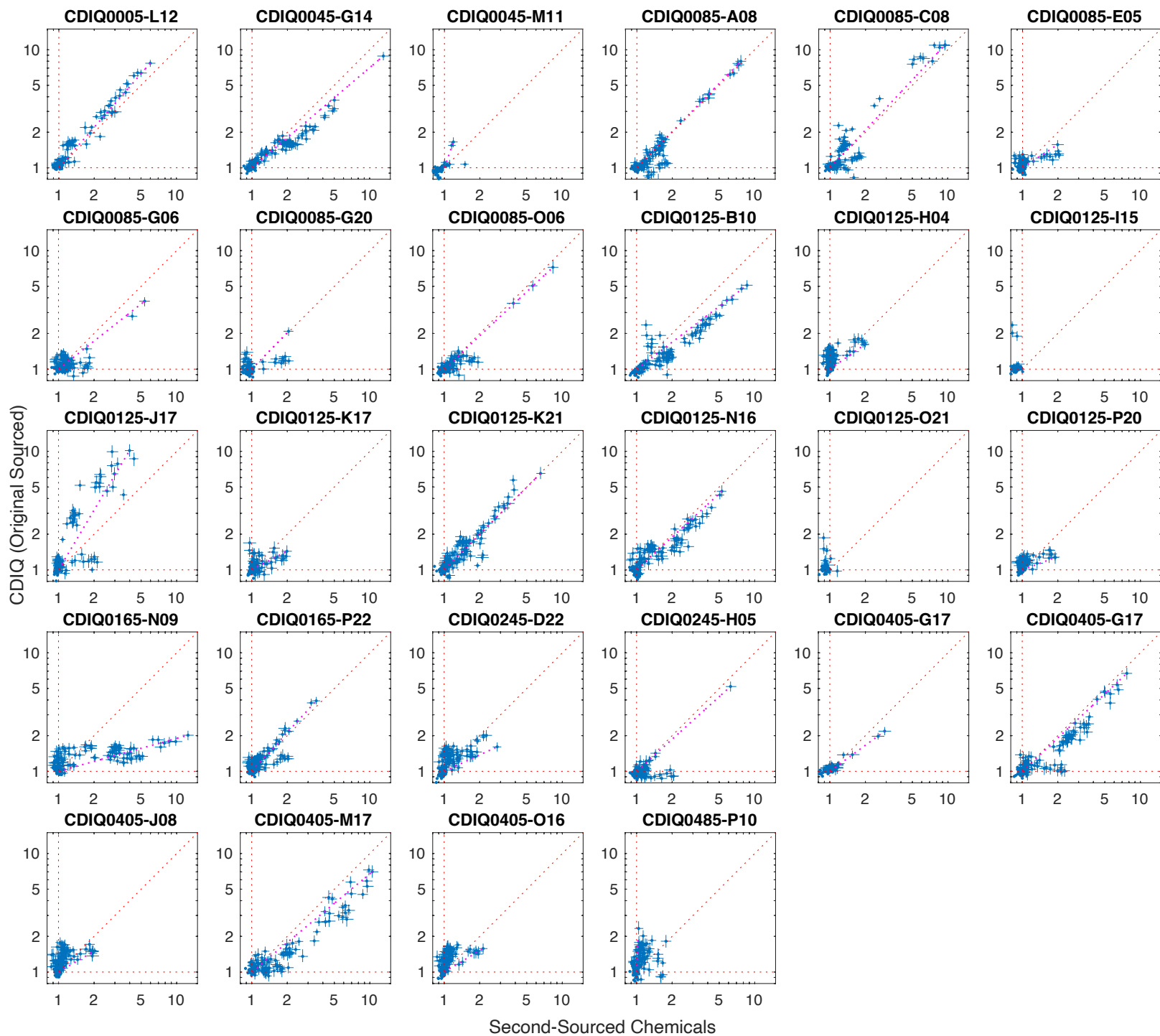

Supplement: S3 Fig — Each subplot shows the fold-change of cleavage of the sequences in the same library in response to two different formulations of purportedly the same compound. Error bars indicate the 95% confidence interval for each measurement based on the number of sequence reads; they are shown for sequences for which the lower-bound of the confidence interval is greater than 1.0 with either formulation. (PDF) [file pone.0273381.s003.pdf]
