# Supplementary material for: Highly multiplexed selection of RNA aptamers against a small molecule library
Source: PLoS One. 2022 Sep 15;17(9):e0273381. doi: 10.1371/journal.pone.0273381 (PMC9477273; doi:10.1371/journal.pone.0273381)
Supplement: S1 File — Comprehensive listing of each sensor identified, chart of fold change in the presence of each compound that affects it, and structures of those compounds in same format as Fig 10. (PDF) [file pone.0273381.s010.pdf]

Hits with fold >= 2.0 (11-Aug-2021 11:20:08)

470840759 GCTGTC ACTGGA TGGACGCT TCCGGT CTGACGA GTCCTG CAACATACCCAGTTGCCCGCTAGCCC CAGGAC GAAACAGC

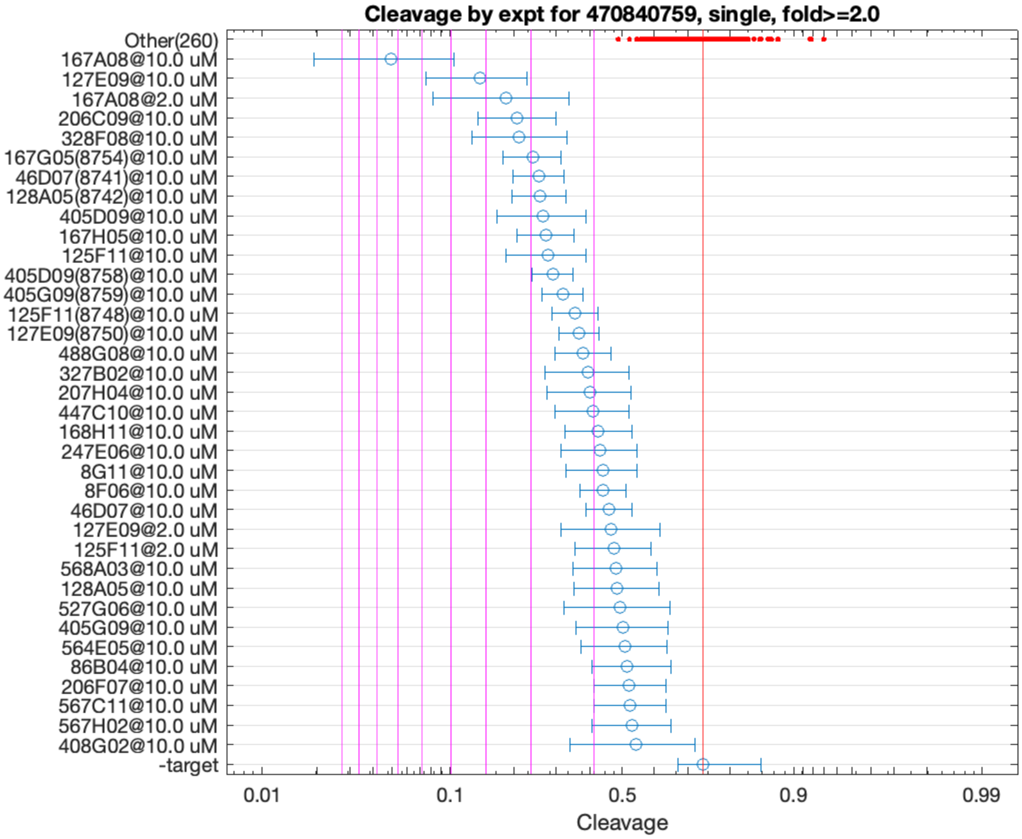

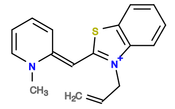

167A08

v>3.1 s=7.5

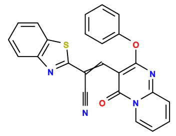

127E09

v>2.1 s=4.1

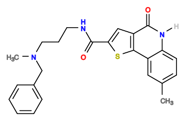

328F08

v>2.2 s=3.2

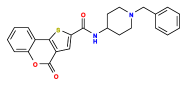

206C09

v>2.6 s=3.2

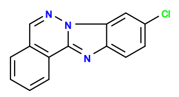

167G05

v>2.2 s=3.0

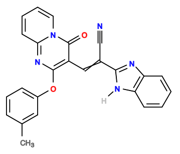

128A05

v>1.8 s=2.9

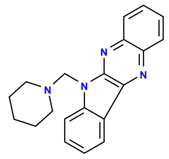

46D07

v>2.2 s=2.9

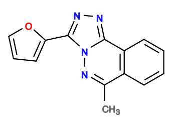

405D09

v>2.5 s=2.8

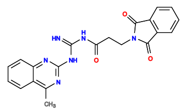

125F11

v>1.9 s=2.7

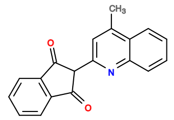

167H05

v>1.7 s=2.7

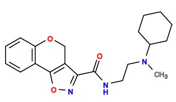

207H09

v>2.6

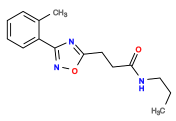

405A07

v>2.6

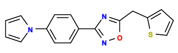

125B05

v>2.6

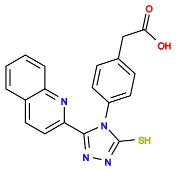

168C07

v>2.6

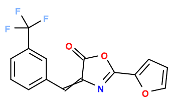

564A05

v>2.6

471146339 GCTGTC ACCGGA GCGGAGA TCTGGT CTGATGA GTCC TGGATACTGAGTAACCACACCGTTACCGCGC GGAC GAAACAGC

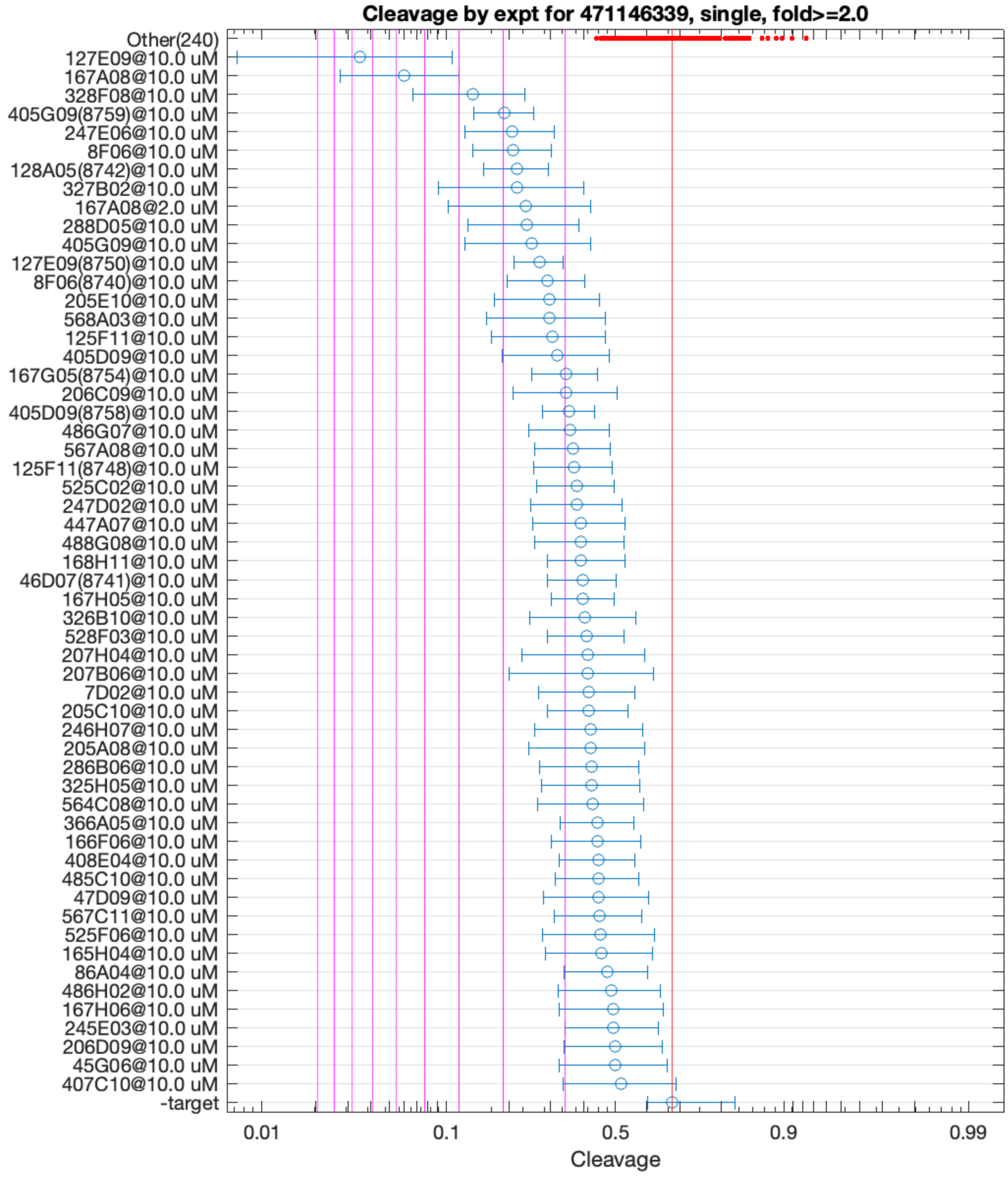

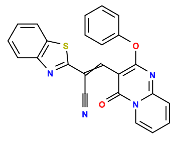

127E09

v>1.7 s=7.5

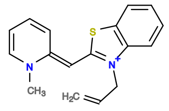

167A08

v>2.3 s=5.6

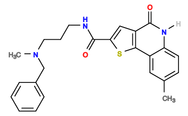

328F08

v>1.6 s=3.6

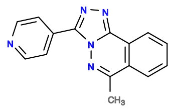

405G09

v>1.9 s=3.0

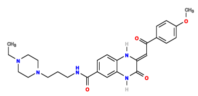

247E06

v>1.8 s=2.8

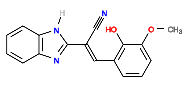

8F06

v>1.4 s=2.8

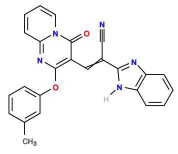

128A05

v>1.1 s=2.8

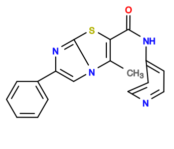

288D05

v>1.3 s=2.6

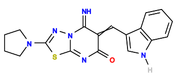

327B02

v>1.0 s=2.6

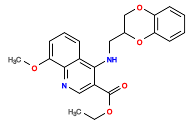

205E10

v>1.7 s=2.2

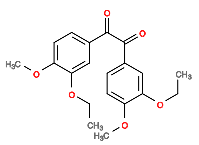

568A03

v>1.2 s=2.2

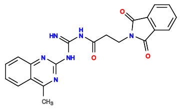

125F11

v>1.2 s=2.1

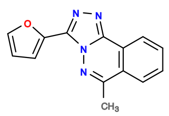

405D09

v>1.6 s=2.1

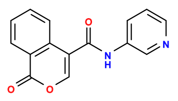

206C11

v>2.0

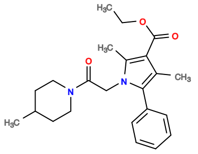

325D07

v>2.0

472067216 GCTGTC ACTGGA GGGAAAG TCTGGT CTGATGA GTCC TTGTCATCGCCGCCACGGATGATGTTGCCC GGAC GAAACAGC

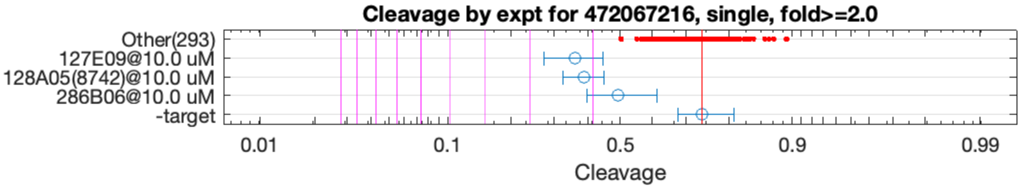

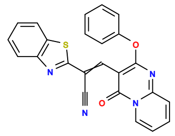

127E09

v>1.2 s=2.3

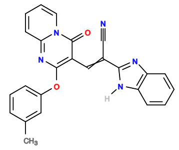

128A05

v>1.2 s=2.2

472764094 GCTGTC ACAGA ATCGCGAG TCTGT CTGATGA GTCC ACGATGGTGCCCCGATAGCGCCCTCTCCGC GGAC GAAACAGC

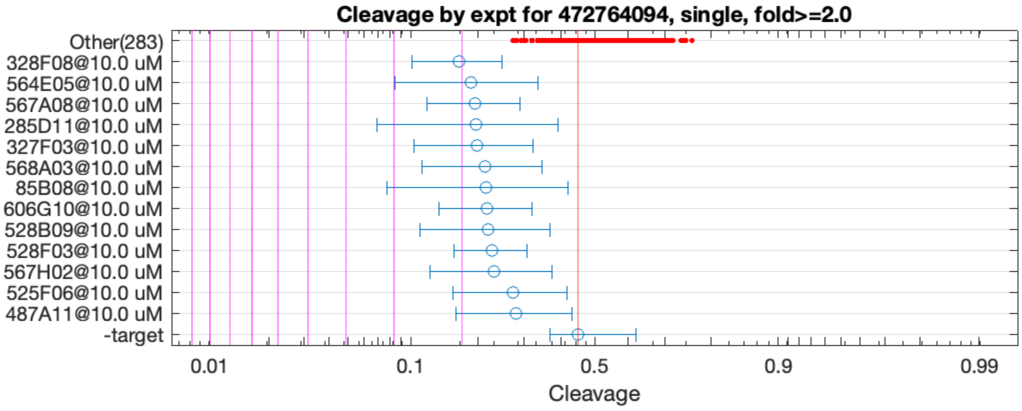

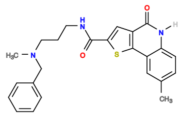

328F08

v>0.8 s=2.0

472766050 GCTGTC ACCGGA ACGCGGA TCTGGT CTGATGA GTCC GTATACCTGCTCGCCGGTGGGTACACGCGA GGAC GAAACAGC

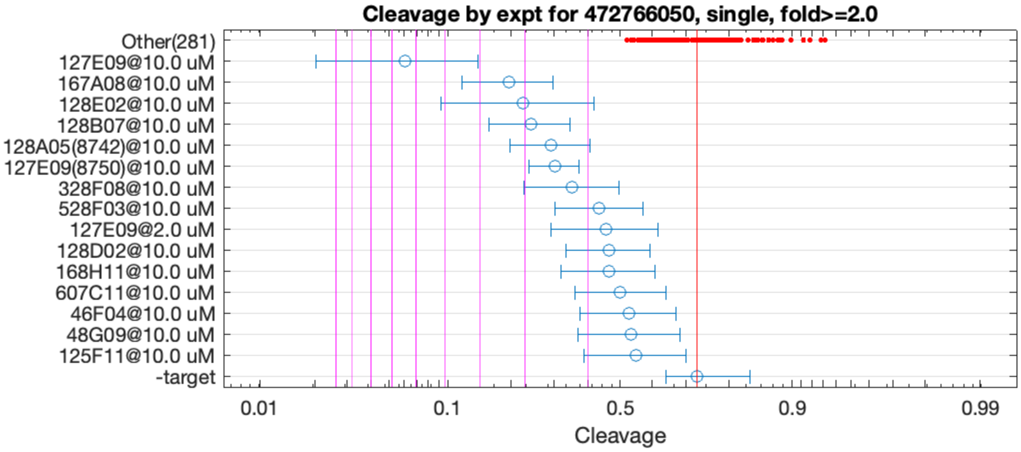

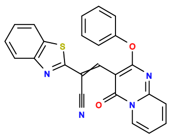

127E09

v>1.2 s=6.5

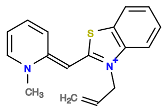

167A08

v>2.0 s=3.4

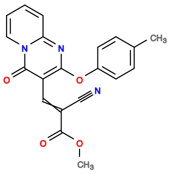

128E02

v>1.5 s=3.0

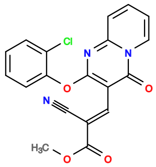

128B07

v>1.6 s=2.9

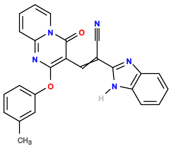

128A05

v>1.8 s=2.6

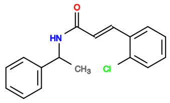

7B04

v>2.5

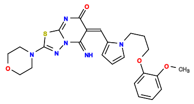

327D09

v>2.5

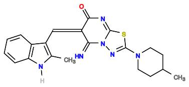

327B10

v>2.3

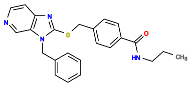

365B03

v>2.3

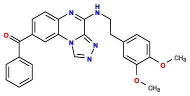

248D10

v>2.3

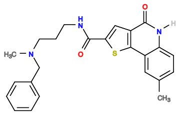

328F08

v>1.8 s=2.2

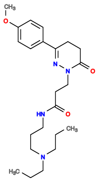

607B11

v>2.2

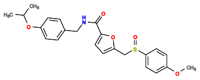

487E05

v>2.2

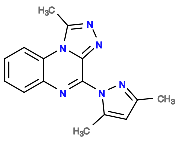

248H10

v>2.2

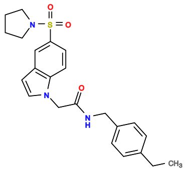

528A04

v>2.1

472786659 GCTGTC ACTGGA TGGAGGCT TCCGGT CTGATGA GTCCTG CAACATACCCAGTTGCCCGCTAGCC CAGGAC GAAACAGC

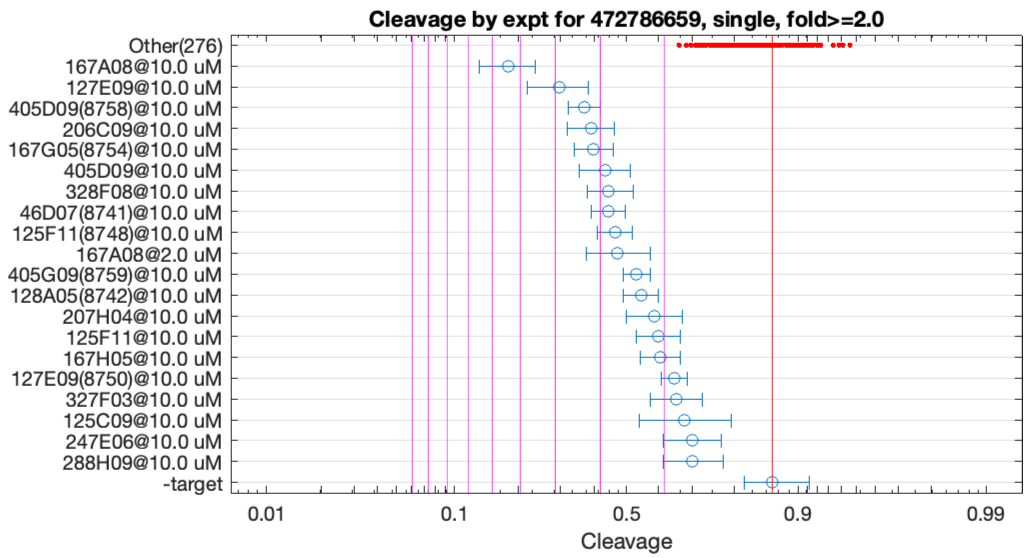

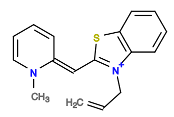

167A08

v>2.2 s=5.4

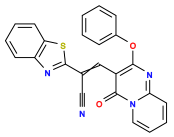

127E09

v>1.7 s=3.9

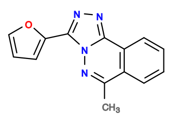

405D09

v>1.9 s=3.3

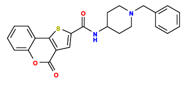

206C09

v>1.8 s=3.2

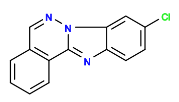

167G05

v>1.9 s=3.1

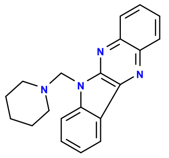

46D07

v>2.0 s=2.9

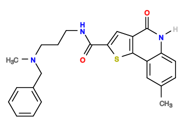

328F08

v>2.2 s=2.8

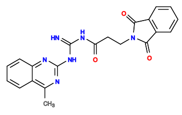

125F11

v>1.6 s=2.7

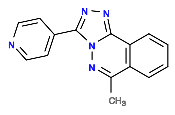

405G09

v>1.8 s=2.4

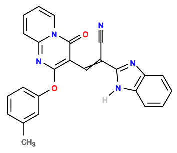

128A05

v>1.5 s=2.3

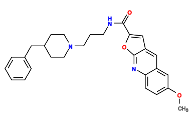

207H04

v>1.8 s=2.2

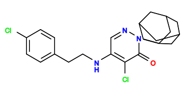

247D09

v>2.1

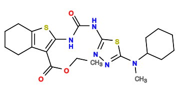

445D06

v>2.0

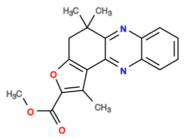

165D05

v>2.0

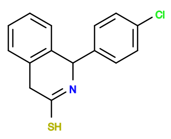

168A06

v>2.0

472911226 GCTGTC ACAGA ATCTGGAG TCTGT CTGATGA GTCC TGCGATGCCTGATACACATGCCTCTCCGC GGAC GAAACAGC

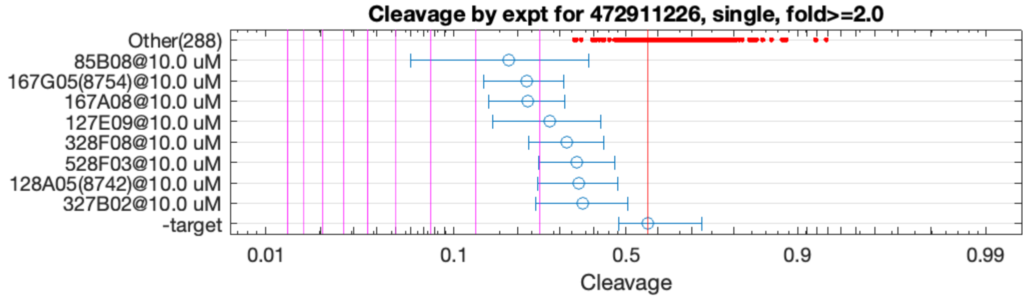

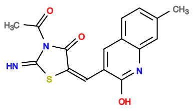

85B08

v>0.9 s=2.5

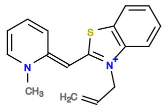

167A08

v>1.1 s=2.2

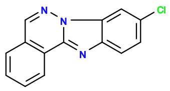

167G05

v>1.0 s=2.1

472924986 GCTGTC ACAG GATACCGTAAA CTGT CTGATGA ATCCAAATCTGCTCGATTTCGTGTGGGTGCGGGGGAC GAAACAGC

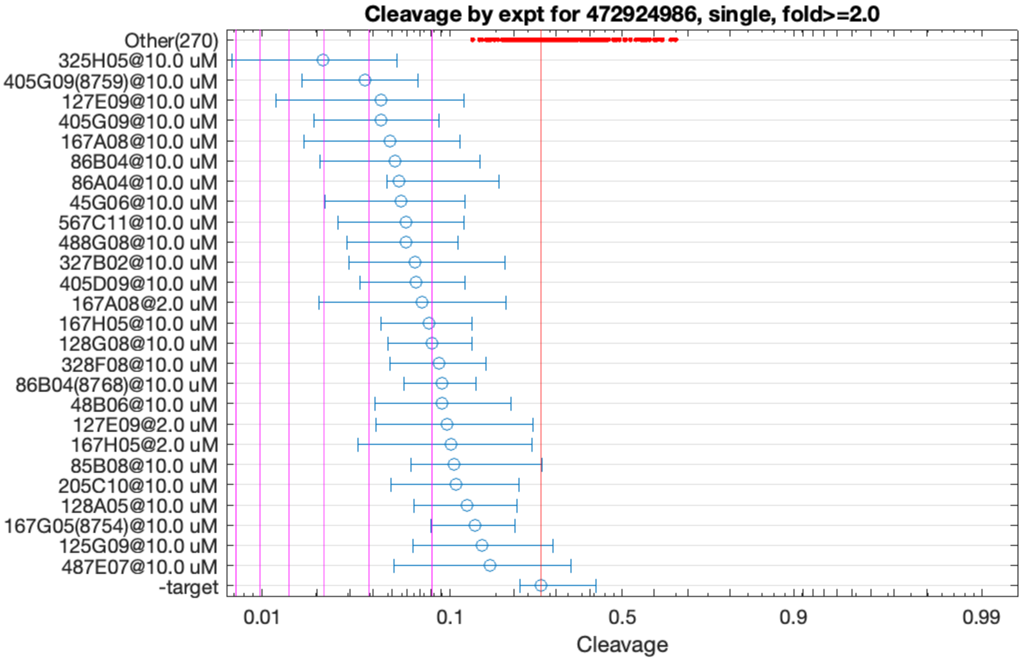

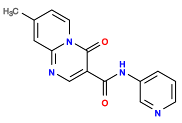

325H05

v>1.3 s=4.0

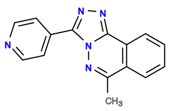

405G09

v>1.3 s=3.0

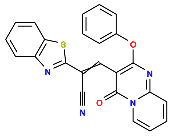

127E09

v>1.2 s=2.8

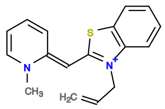

167A08

v>1.2 s=2.6

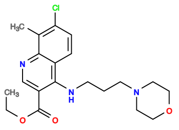

86B04

v>1.4 s=2.5

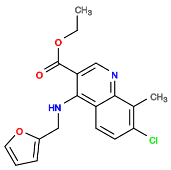

45G06

v>1.4 s=2.5

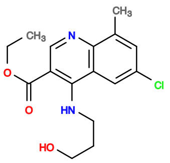

86A04

v>1.0 s=2.5

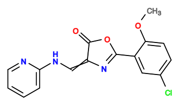

567C11

v>1.2 s=2.4

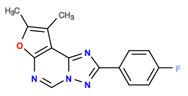

488G08

v>1.2 s=2.4

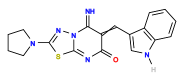

327B02

v>1.1 s=2.2

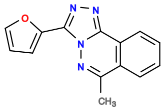

405D09

v>1.1 s=2.2

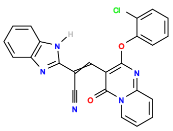

128G08

v>1.3 s=2.0

472985468 GCTGTC ACCGGA GCGGAGA TCTGGT CTGAAGA GTCC TGGATACTGAGTAACAACACCGTTACCGCGC GGAC GAAACAGC

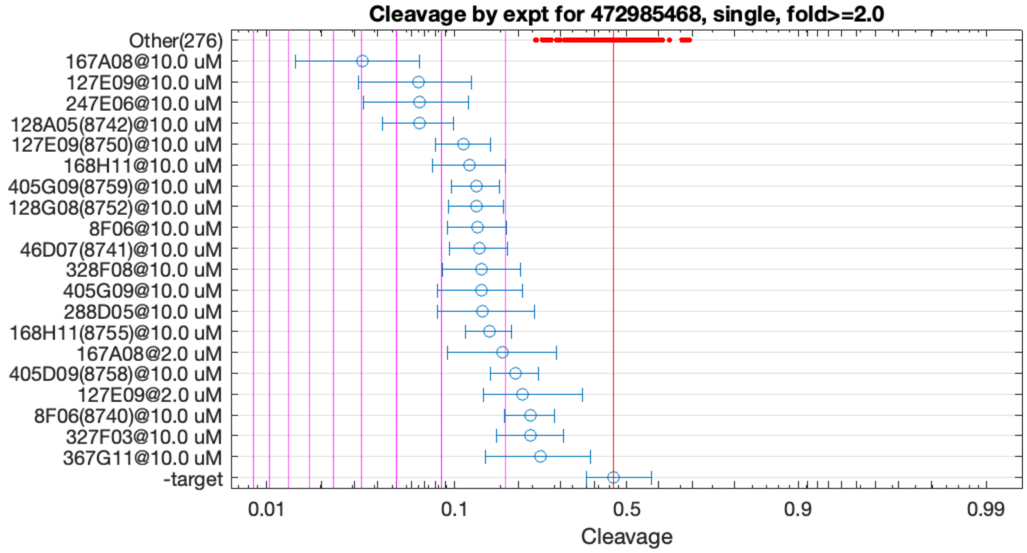

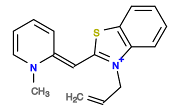

167A08

v>2.9 s=5.2

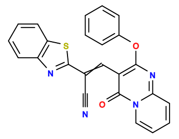

127E09

v>2.0 s=3.5

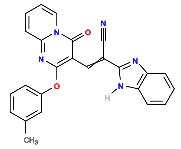

128A05

v>1.8 s=3.5

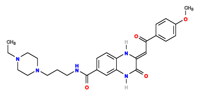

247E06

v>2.2 s=3.4

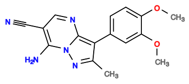

168H11

v>1.8 s=2.5

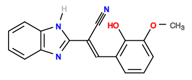

8F06

v>1.6 s=2.4

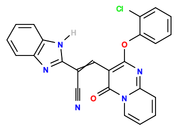

128G08

v>2.2 s=2.4

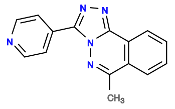

405G09

v>2.1 s=2.4

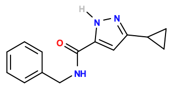

525B07

v>2.3

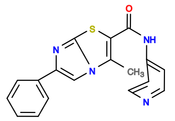

288D05

v>1.9 s=2.3

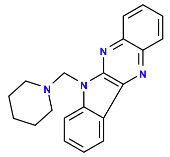

46D07

v>1.8 s=2.3

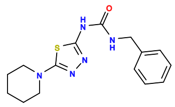

445A03

v>2.3

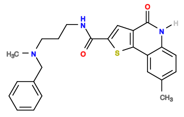

328F08

v>1.7 s=2.3

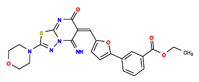

327D08

v>2.3

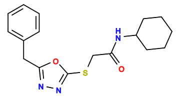

246G05

v>2.3

472986817 GCTGTC ACCGGA GGGAAAG TCTGGT CTGATGA GTCC TTGTCATCGCCACCTCGGATGATGTTGCCC GGAC GAAACAGC

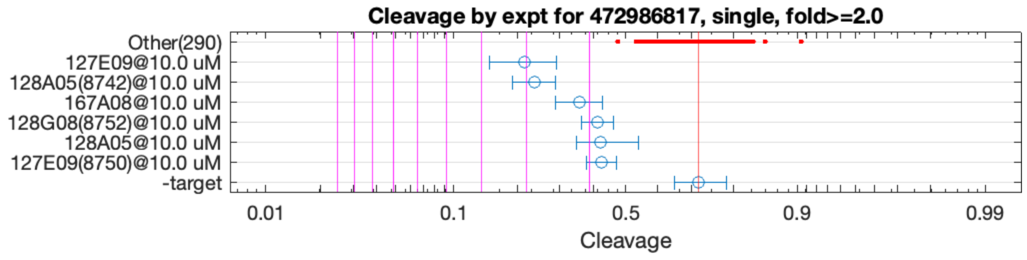

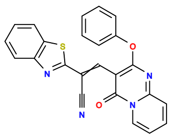

127E09

v>1.3 s=3.0

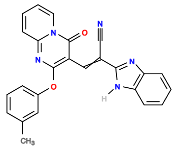

128A05

v>1.6 s=2.9

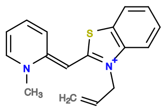

167A08

v>1.3 s=2.1

473025843 GCTGTC ACCGGA GTTCGAGTTGTCGATTCGATTGAGGGACGG TCCGGT CTGACGA GTCCA CCATCC TGGAC GAAACAGC

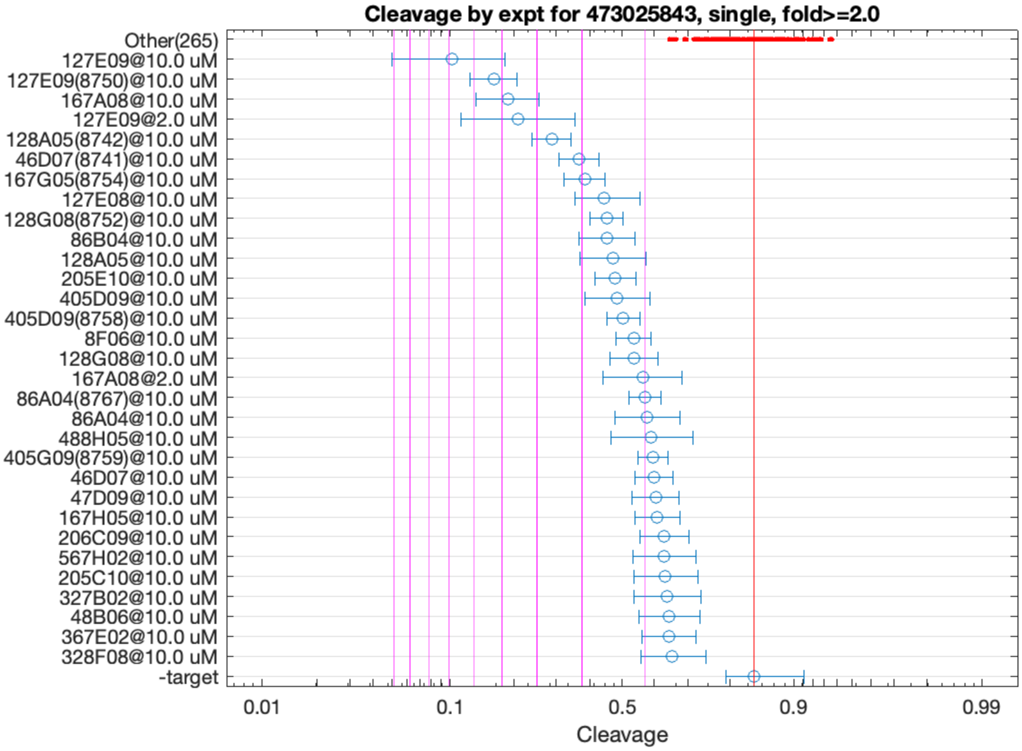

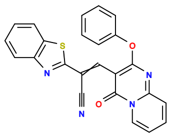

127E09

v>4.0 s=6.9

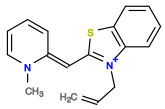

167A08

v>2.4 s=4.8

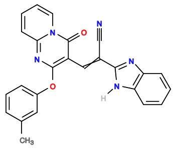

128A05

v>2.0 s=3.6

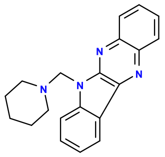

46D07

v>1.9 s=3.1

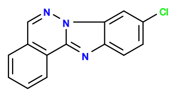

167G05

v>2.0 s=2.9

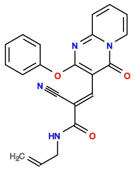

127E08

v>2.1 s=2.6

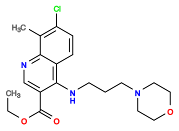

86B04

v>2.2 s=2.6

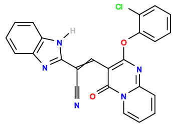

128G08

v>2.1 s=2.6

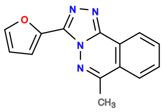

405D09

v>1.7 s=2.4

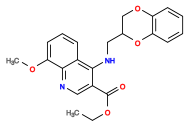

205E10

v>1.7 s=2.4

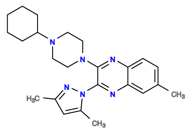

248E02

v>2.2

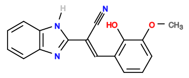

8F06

v>1.4 s=2.2

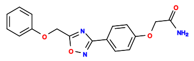

125D05

v>2.2

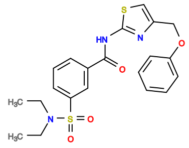

88G05

v>2.1

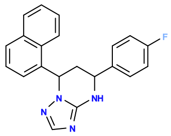

125H02

v>2.1

473078304 GCTGTC ACTGGA GGGAAAG TCTGGT CTGATGA GTCC TTGTCATCGCCACCATGGATGATGTTGCCC GGAC GAAACAGC

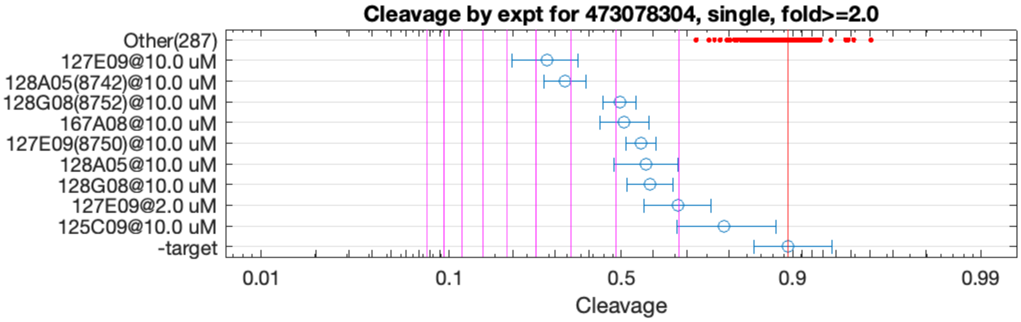

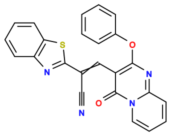

127E09

v>1.7 s=4.6

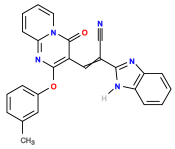

128A05

v>1.8 s=4.2

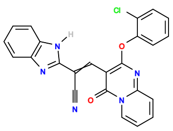

128G08

v>2.1 s=2.9

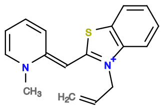

167A08

v>1.5 s=2.8

473081357 GCTGTC ACTGGA TCCAAGCT TCCGGT CTGATGA GTCCA ATGAGGGTGCTTTCACTTCTCGCAACGG TGGAC GAAACAGC

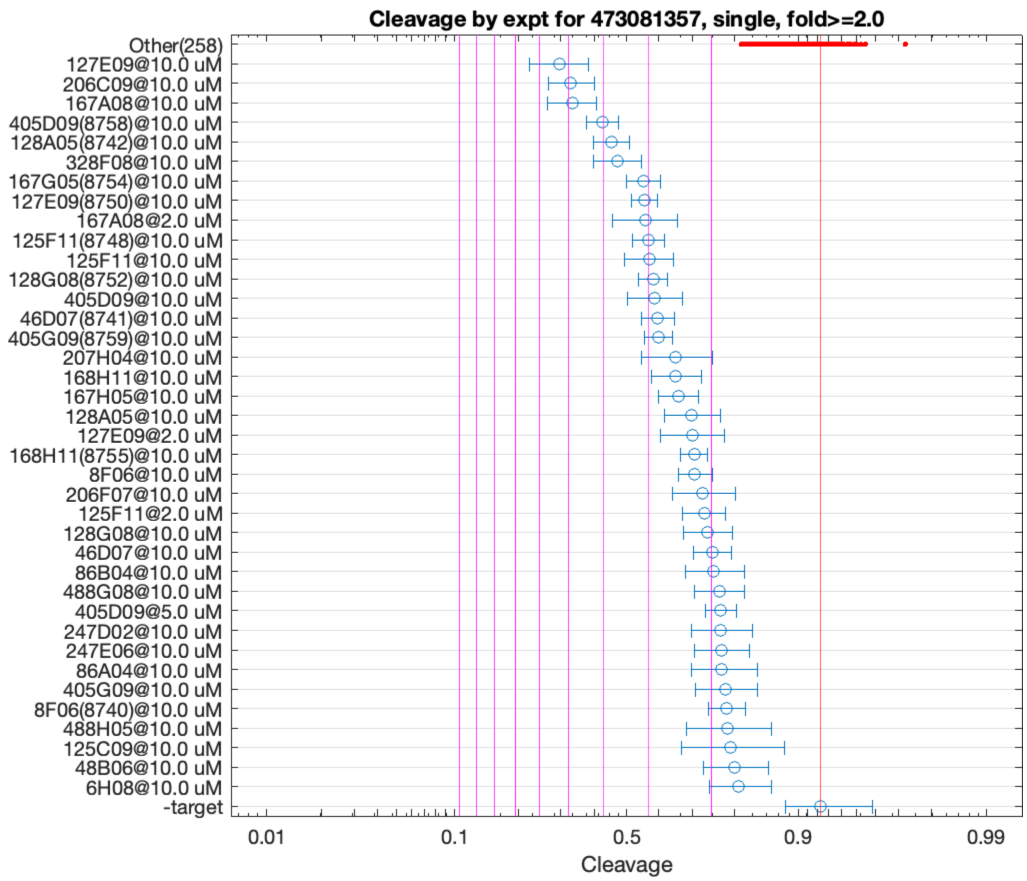

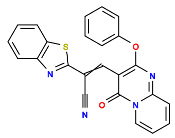

127E09

v>2.5 s=5.3

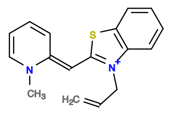

167A08

v>2.9 s=4.9

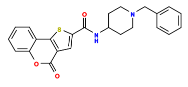

206C09

v>3.1 s=4.9

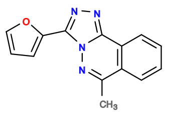

405D09

v>2.5 s=4.1

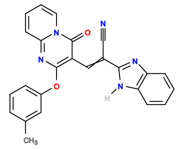

128A05

v>2.3 s=3.8

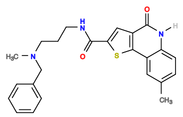

328F08

v>2.7 s=3.6

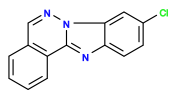

167G05

v>2.3 s=3.1

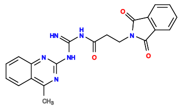

125F11

v>2.1 s=3.0

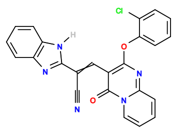

128G08

v>2.8 s=2.9

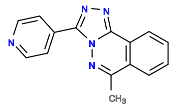

405G09

v>2.3 s=2.8

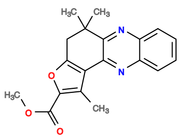

165D05

v>2.8

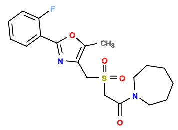

286G08

v>2.8

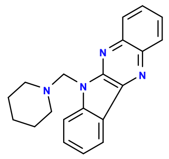

46D07

v>2.4 s=2.8

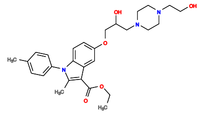

87H04

v>2.8

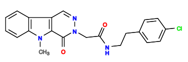

368H06

v>2.7

473082538 GCTGTC ACTGGA TCGATGCT TCCGGT CTGACGA GTCC TTTCCCAGGCAGAAATTTGCTCGCGATCGT GGAC GAAACAGC

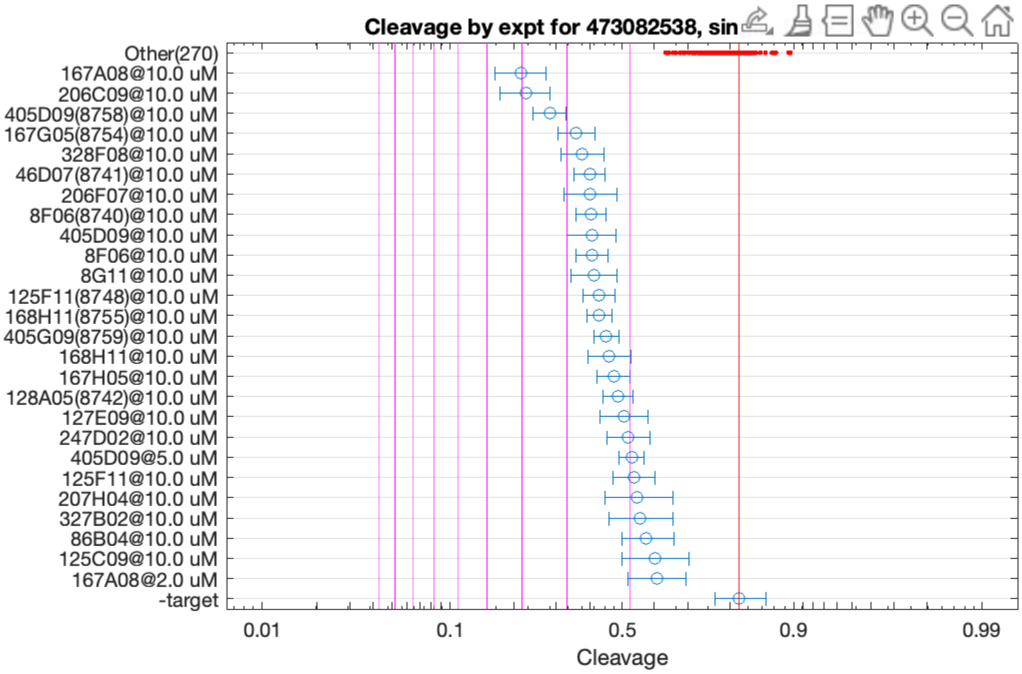

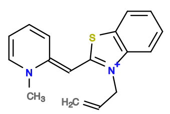

167A08

v>1.6 s=4.0

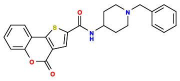

206C09

v>2.3 s=3.9

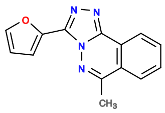

405D09

v>1.8 s=3.4

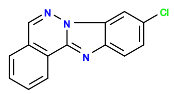

167G05

v>1.6 s=2.8

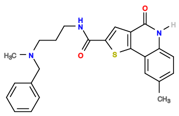

328F08

v>1.9 s=2.7

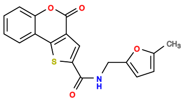

206F07

v>1.8 s=2.6

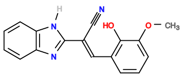

8F06

v>1.4 s=2.6

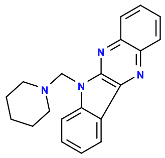

46D07

v>1.7 s=2.6

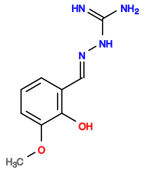

8G11

v>1.9 s=2.5

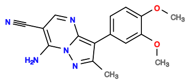

168H11

v>1.6 s=2.5

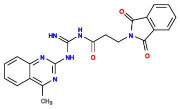

125F11

v>1.4 s=2.5

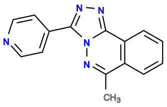

405G09

v>1.5 s=2.3

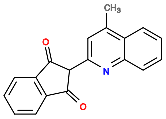

167H05

v>1.5 s=2.2

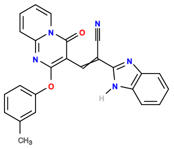

128A05

v>1.3 s=2.2

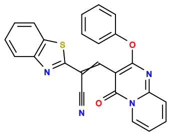

127E09

v>1.6 s=2.1

473086978 GCTGTC ACTGGA TGTGGTGC TCCGGT CTGACGA GTCC GATGTACGTTTGTCGACGCGCATCCCTTAA GGAC GAAACAGC

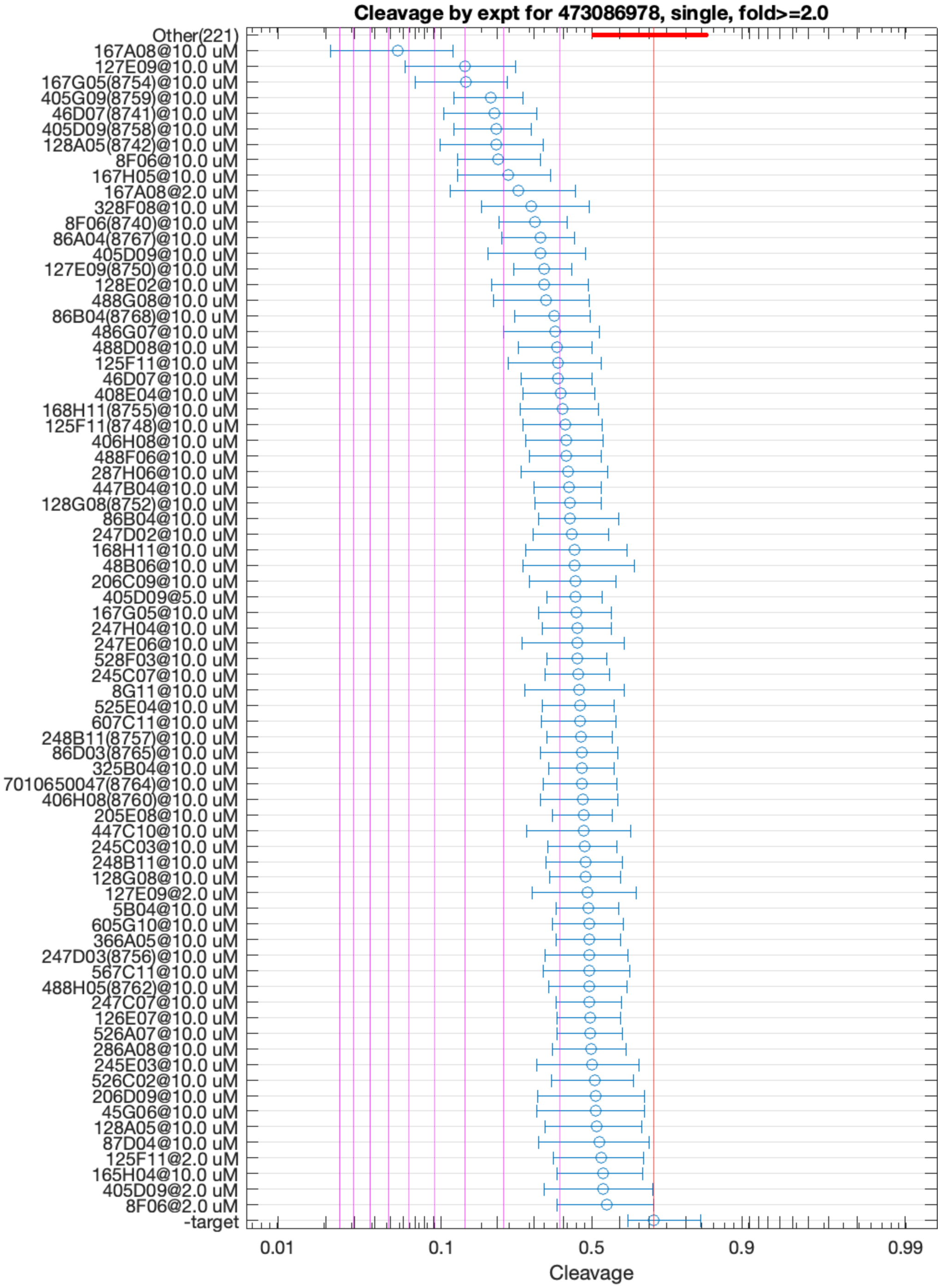

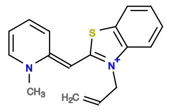

167A08

v>2.8 s=6.3

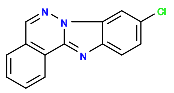

167G05

v>2.5 s=4.2

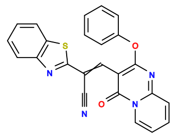

127E09

v>2.3 s=4.0

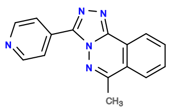

405G09

v>2.3 s=3.3

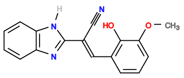

8F06

v>2.1 s=3.2

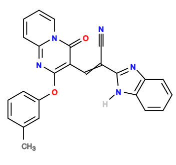

128A05

v>1.8 s=3.2

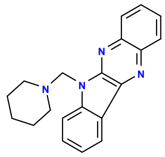

46D07

v>2.4 s=3.2

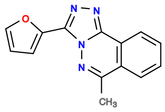

405D09

v>2.4 s=3.2

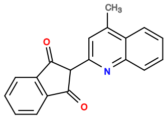

167H05

v>1.8 s=2.9

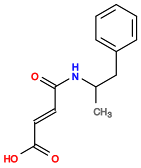

6E06

v>2.7

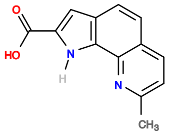

208E04

v>2.7

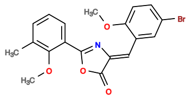

566G03

v>2.6

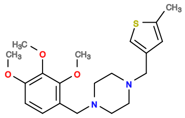

486G03

v>2.6

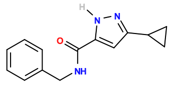

525B07

v>2.6

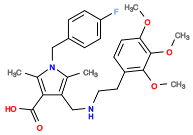

366H05

v>2.6

473362869 GCTGTC ACTGGA TCGACGCT TCCGGT CTGACGA GTCC TTTCCCAGGCAGAAATTTGCTCGCGATCGC GGAC GAAACAGC

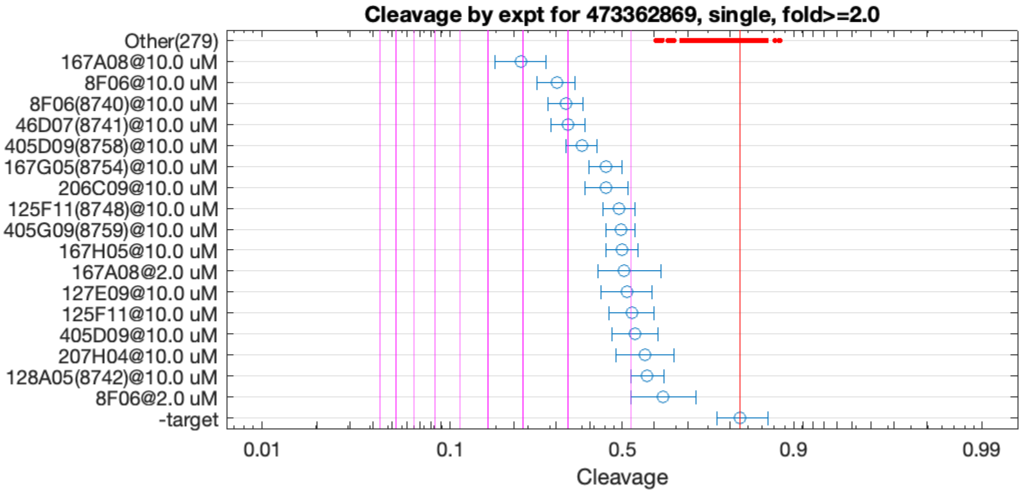

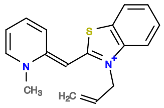

167A08

v>1.8 s=4.0

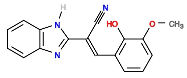

8F06

v>1.5 s=3.2

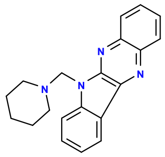

46D07

v>1.6 s=3.0

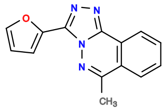

405D09

v>1.6 s=2.7

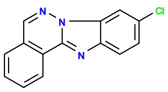

167G05

v>1.5 s=2.4

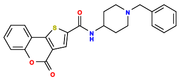

206C09

v>1.6 s=2.3

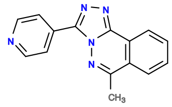

405G09

v>1.5 s=2.2

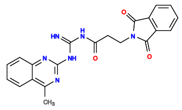

125F11

v>1.5 s=2.1

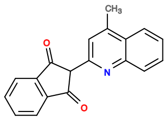

167H05

v>1.5 s=2.1

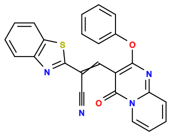

127E09

v>1.5 s=2.0

476982823 GCTGTC AC TGGAAAACGTAAACT GT CTGATGA GTCC AAATCTGCTCGATTTCGTGTGGGTGCGTG GGAC GAAACAGC

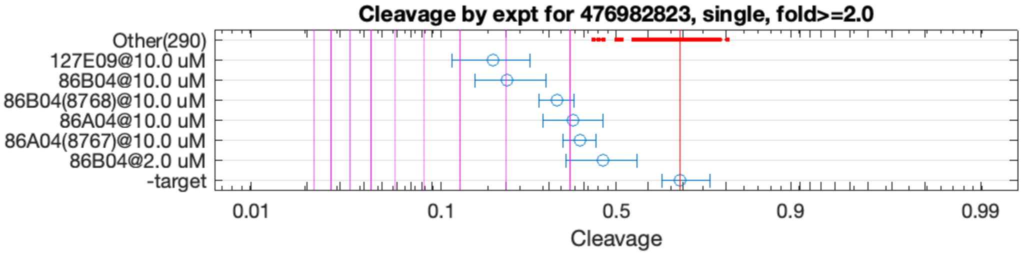

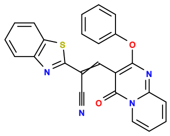

127E09

v>1.4 s=3.3

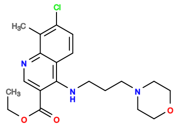

86B04

v>1.7 s=2.9

476984717 GCTGTC ACTGGA AACGAAGG TCCGGT CTGAAGA GTCC GCCTAAGCGCTGCTCACGCAGTGGCTCGTG GGAC GAAACAGC

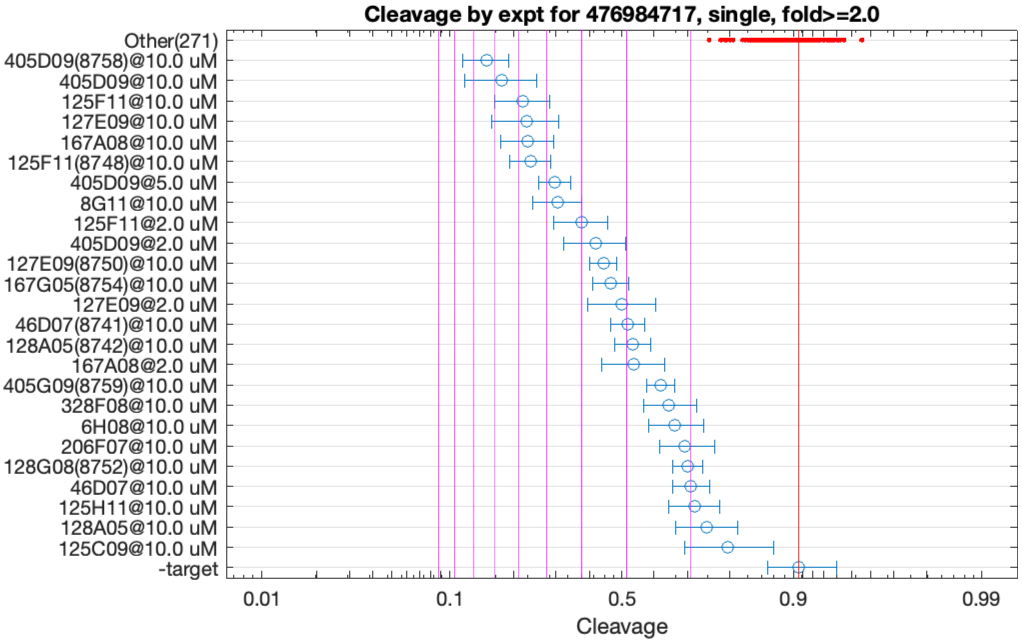

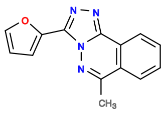

405D09

v>5.9 s=7.2

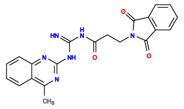

125F11

v>2.5 s=6.0

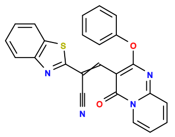

127E09

v>3.3 s=5.6

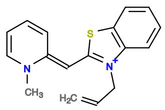

167A08

v>3.2 s=5.6

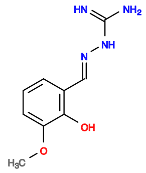

8G11

v>4.7 s=4.7

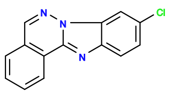

167G05

v>2.6 s=3.4

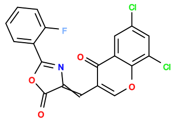

564E04

v>3.1

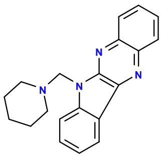

46D07

v>2.6 s=3.0

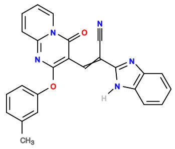

128A05

v>2.5 s=2.9

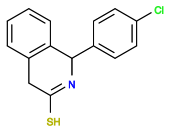

168A06

v>2.9

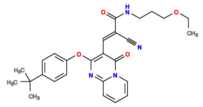

127C11

v>2.9

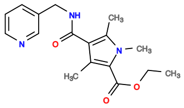

326A05

v>2.9

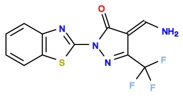

48A05

v>2.9

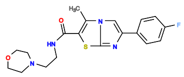

288G06

v>2.8

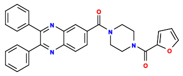

568D05

v>2.8

477172569 GCTGTC ACTGGAT TGAACTTCTGCGTAGGTTCAGGCACCGTT ATCCGGT CCGATGA GTCC CTGGTGTA GGAC GAAACAGC

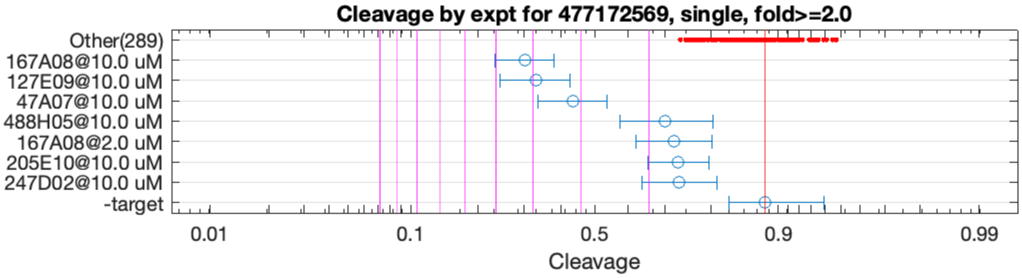

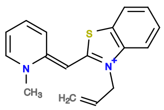

167A08

v>1.5 s=4.1

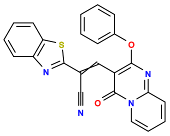

127E09

v>1.3 s=3.9

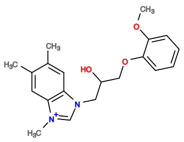

47A07

v>1.6 s=3.2

477174303 GCTGTC AC TGGATTGGCGTAAATCC GT CTGATGA GTCC AGCGATGCCTGATACACATGCCTCTCCGCC GGAC GAAACAGC

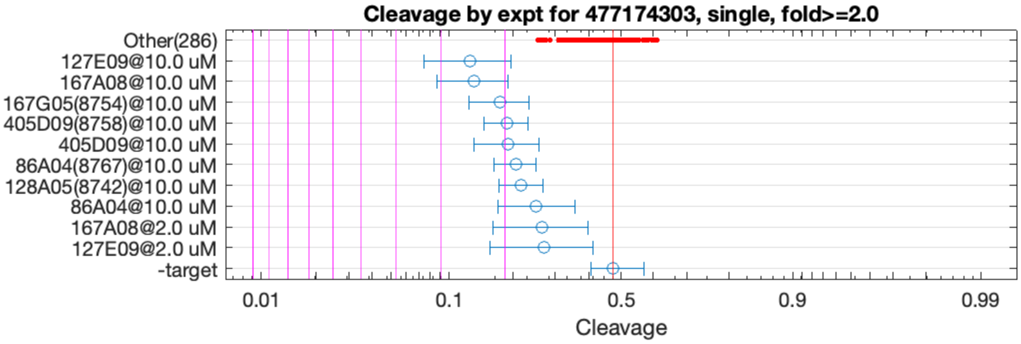

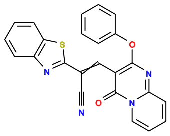

127E09

v>1.4 s=2.5

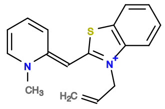

167A08

v>1.8 s=2.4

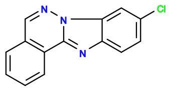

167G05

v>1.5 s=2.1

477791857 GCTGTC ACAGGATCGGGAGA CTGTCTGA GGAGTCCTACGGCCGGAC GAAACAGC

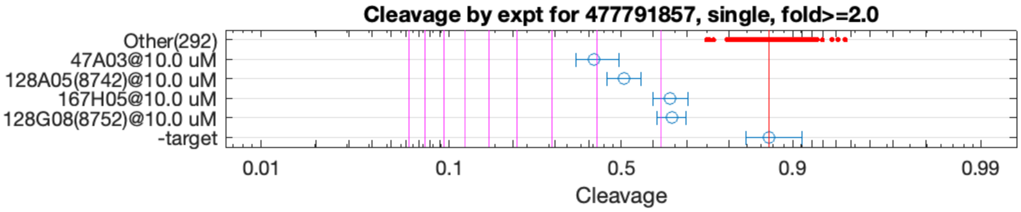

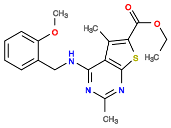

47A03

v>1.9 s=3.1

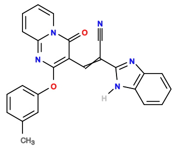

128A05

v>1.4 s=2.6

478014511 GCTGTC ACTGGA ACGCGGA TCTGGT CTGATGA GTCC GTATACCTGCTCACCGGTGGGTACACGCGA GGAC GAAACAGC

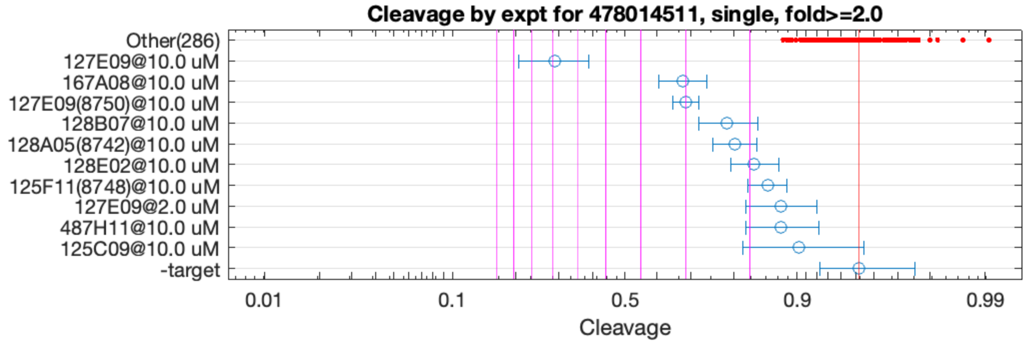

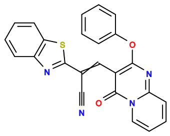

127E09

v>1.4 s=7.0

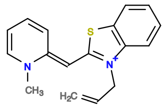

167A08

v>1.4 s=3.1

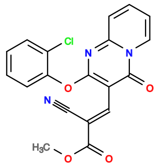

128B07

v>1.3 s=2.3

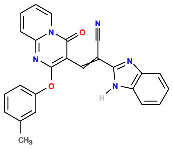

128A05

v>1.0 s=2.2

482370107 GCTGTC ACTGGATGCGAT CTGATGA GTCC ACGTATGACGAAACAGCTCTGCCTGGCCCG GGAC GAAACAGC

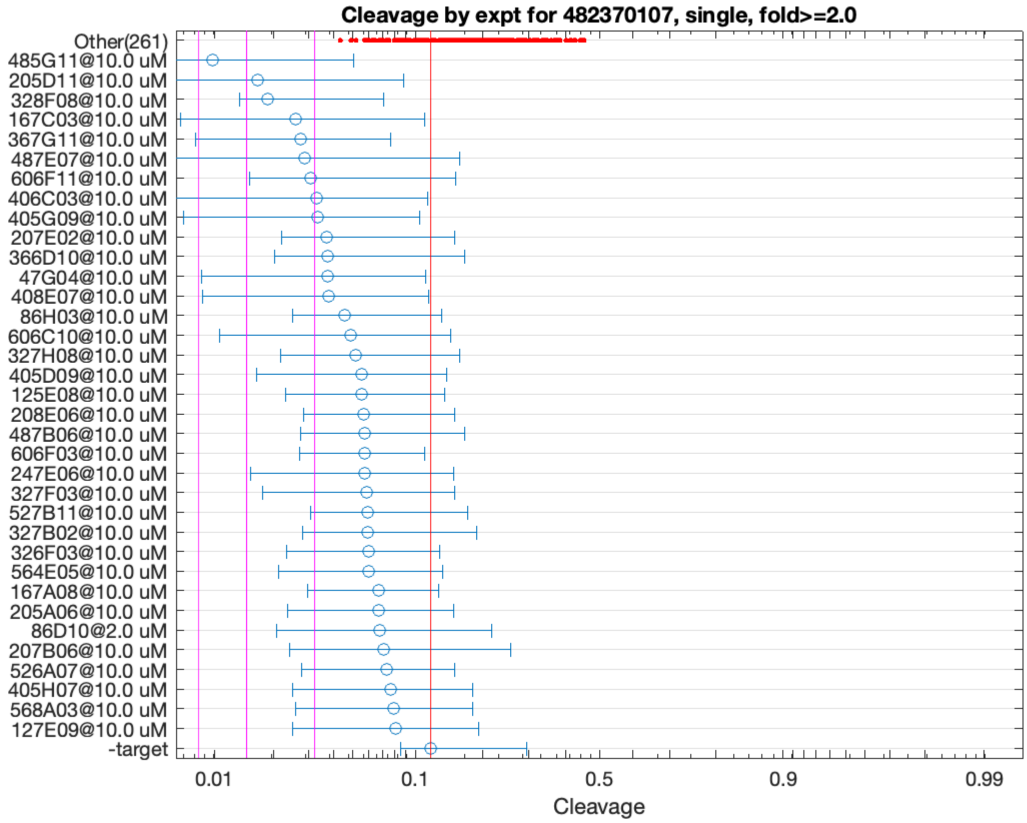

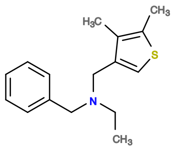

485G11

v>0.5 s=3.7

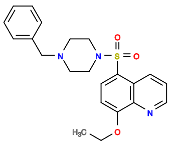

205D11

v>0.5 s=2.7

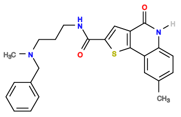

328F08

v>0.5 s=2.6

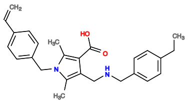

367G11

v>0.5 s=2.3

483584637 GCTGTC ACTGGA GCGGAGA TCTGGT CTGATGA GTCC TGGATACTGAGTAACCACACCGTTACCGCGC GGAC GAAACAGC

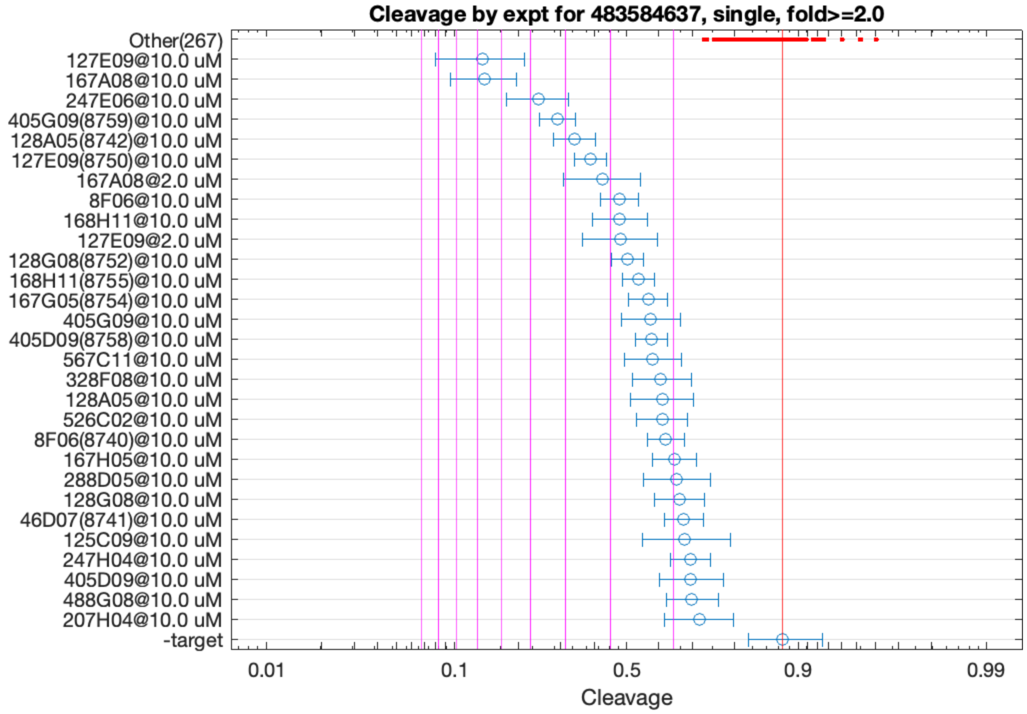

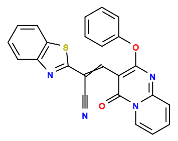

127E09

v>2.7 s=6.8

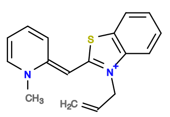

167A08

v>3.3 s=6.7

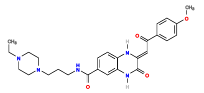

247E06

v>2.5 s=4.7

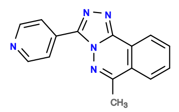

405G09

v>2.6 s=4.2

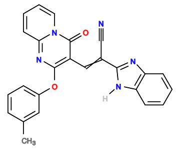

128A05

v>2.0 s=3.7

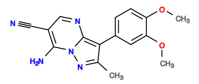

168H11

v>2.2 s=2.8

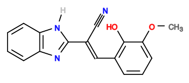

8F06

v>1.7 s=2.8

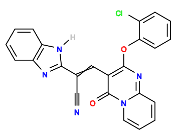

128G08

v>2.5 s=2.7

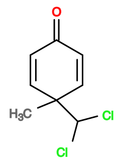

7A08

v>2.4

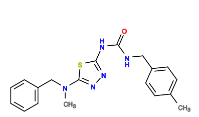

445E03

v>2.4

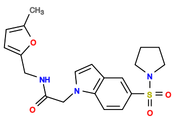

528B04

v>2.4

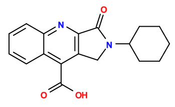

208A06

v>2.4

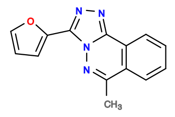

405D09

v>1.8 s=2.3

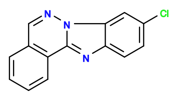

167G05

v>2.1 s=2.3

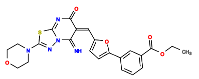

327D08

v>2.3

485142034 GCTGTC ACTGGA GTTCGAGTTGTCGATTCGATTGAGGGACGG TCCGGT CTGACGA GTCCA CCATCC TGGAC GAAACAGC

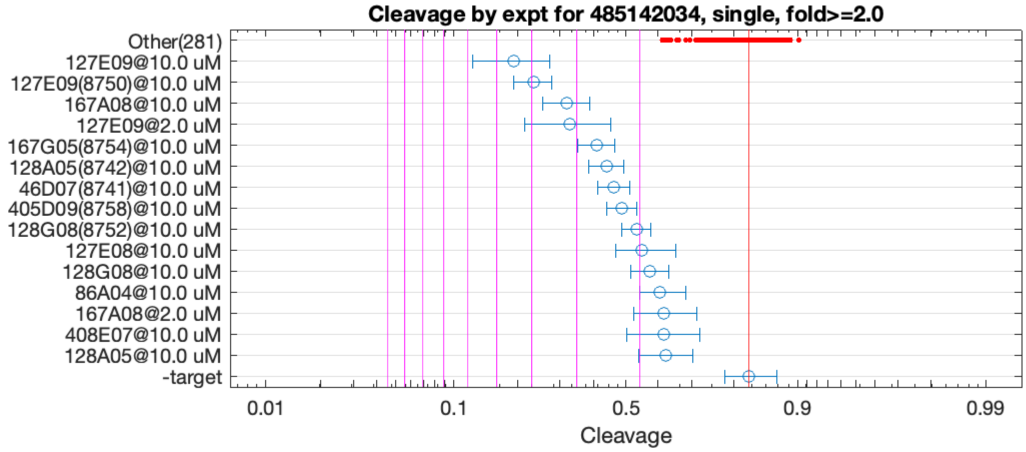

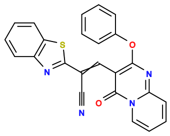

127E09

v>2.7 s=4.5

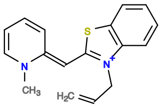

167A08

v>1.7 s=3.2

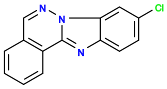

167G05

v>1.6 s=2.7

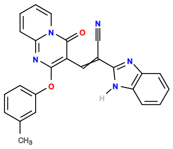

128A05

v>1.4 s=2.5

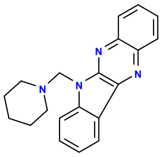

46D07

v>1.5 s=2.4

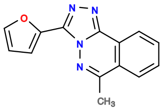

405D09

v>1.4 s=2.2

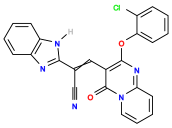

128G08

v>1.7 s=2.1

485200498 GCTGTC ACTGGA GACGAAGG TCCAGT CTGAAGA GTCC GCCTAAGCGCTGCTCACACAGTGGCTCGTG GGAC GAAACAGC

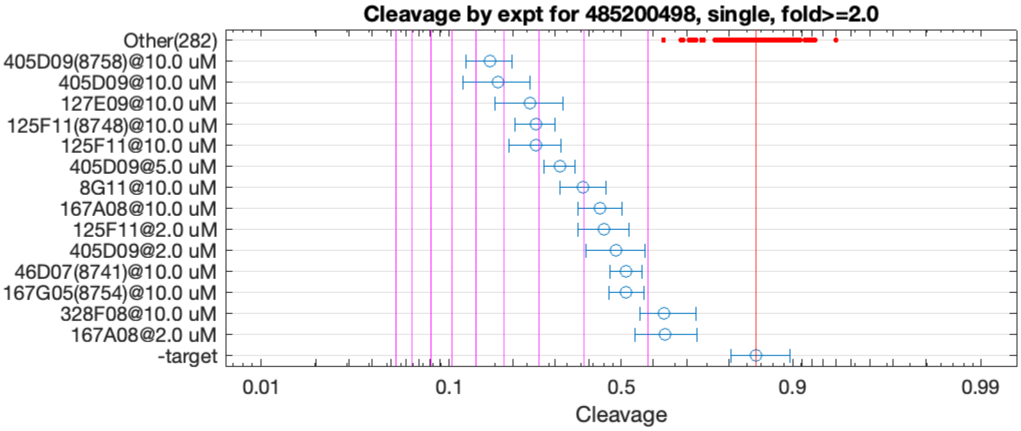

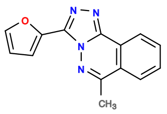

405D09

v>4.0 s=5.5

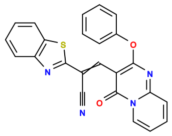

127E09

v>1.9 s=4.2

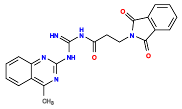

125F11

v>1.5 s=4.2

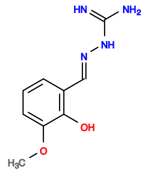

8G11

v>3.1 s=3.0

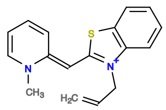

167A08

v>1.9 s=2.7

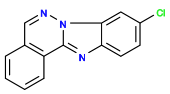

167G05

v>1.5 s=2.3

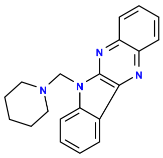

46D07

v>1.7 s=2.3

487839220 GCTGTC ACTGGA TGCATGACCATGTTTGGTGTGCTGAGCGGC TCCGGT CTGATGA GTTC GTGGCG GGAC GAAACAGC

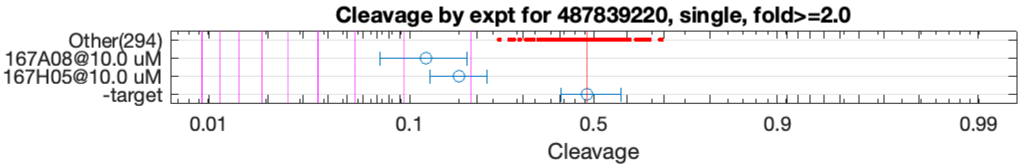

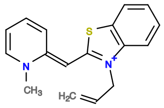

167A08

v>1.1 s=2.6

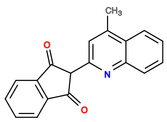

167H05

v>1.1 s=2.2

505772600 GCTGTC ACTGGA GCGGAGA TCTGGT CTGATGA GTCC TGGATTCTGAGTAACAACACCGTTACCGCGC GGAC GAAACAGC

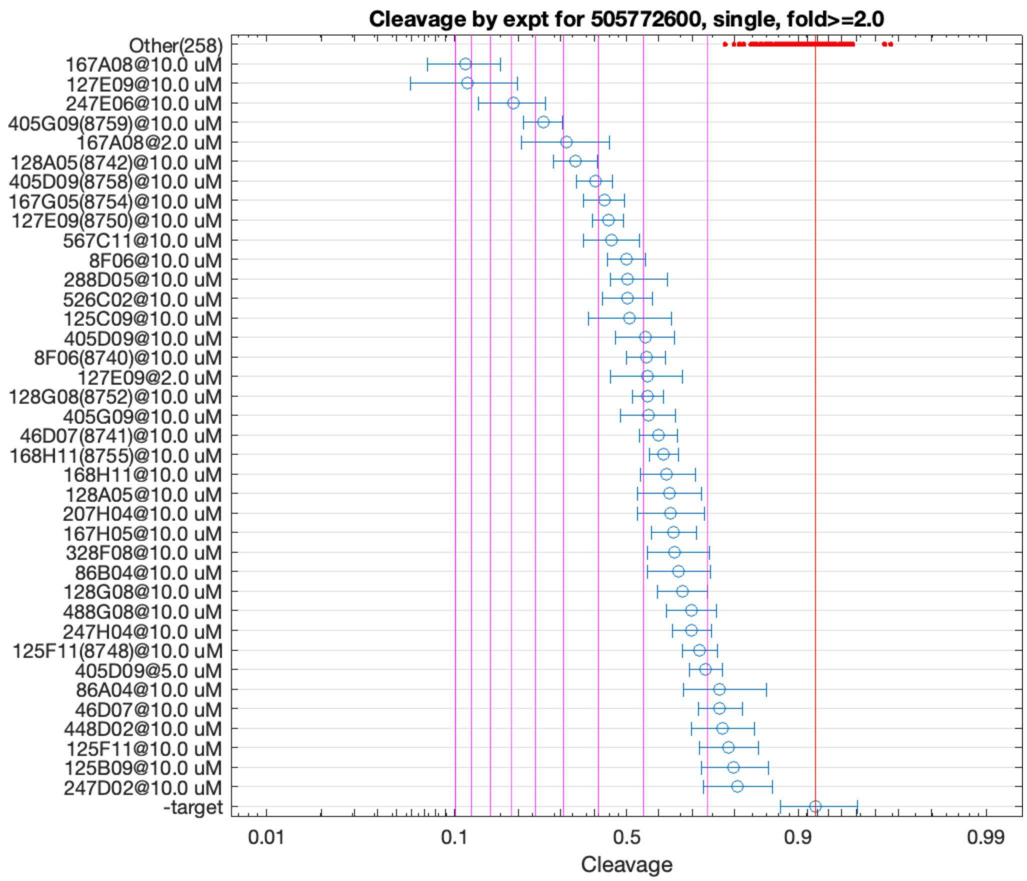

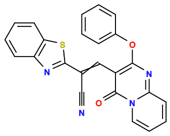

127E09

v>3.1 s=9.4

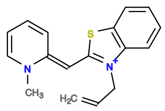

167A08

v>4.9 s=9.4

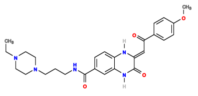

247E06

v>3.0 s=6.8

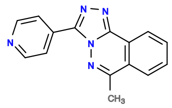

405G09

v>3.2 s=5.6

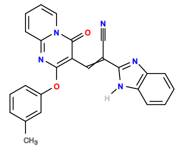

128A05

v>2.4 s=4.7

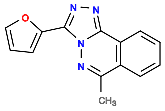

405D09

v>2.4 s=4.1

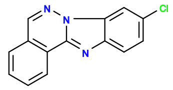

167G05

v>2.4 s=3.8

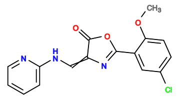

567C11

v>2.6 s=3.6

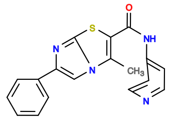

288D05

v>2.7 s=3.4

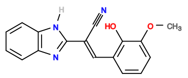

8F06

v>2.0 s=3.3

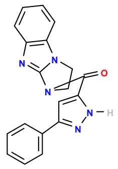

526C02

v>2.8 s=3.3

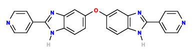

125C09

v>2.7 s=3.3

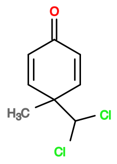

7A08

v>3.0

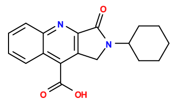

208A06

v>3.0

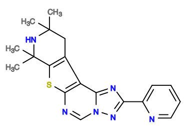

125D07

v>2.9

510203249 GCTGTC ACTGGA AGGCACGTCTTTGAGTAGCTAAAACTGGAA TCCGGT CTGACGA GTCC GATACGTTGGCGAGTAGTTGCATGTGCCCG GGAC GAAACAGC

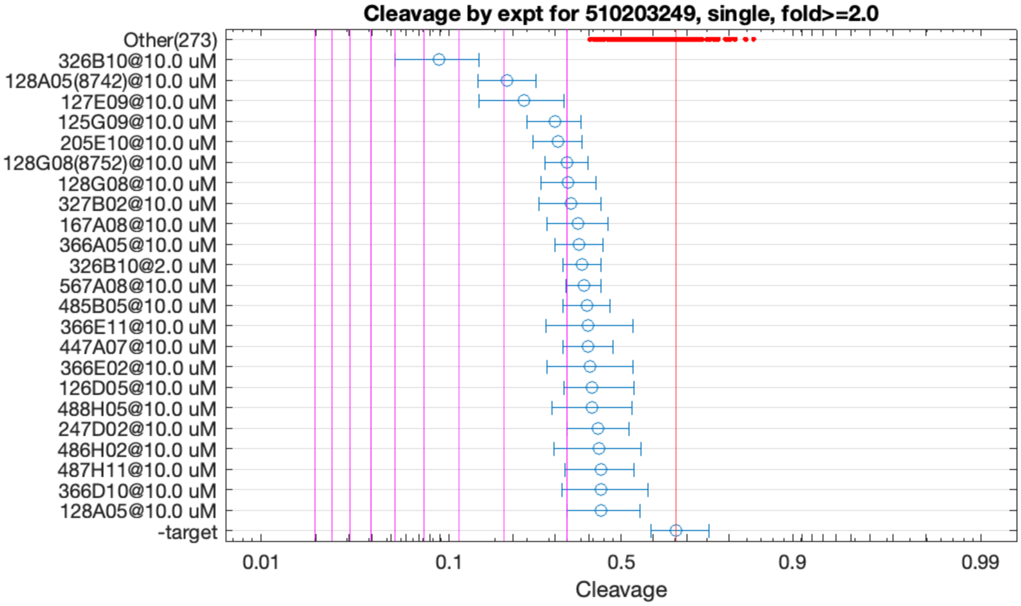

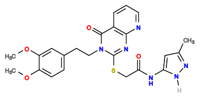

326B10

v>1.6 s=4.5

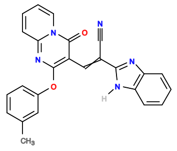

128A05

v>1.2 s=3.0

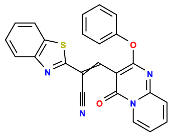

127E09

v>1.1 s=2.6

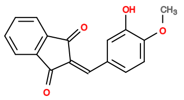

125G09

v>1.2 s=2.1

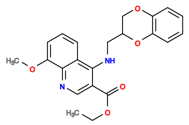

205E10

v>1.1 s=2.1

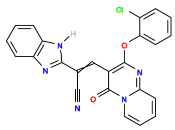

128G08

v>1.4 s=2.0

510203337 GCTGTC ACTGGA CCTGGTGGAGAGATTACTTGGGTGTTGCGA TCCGGT CTGATGA GTCC AATACGTGTTCAAGCTTCTCCTCTTACGGC GGAC GAAACAGC

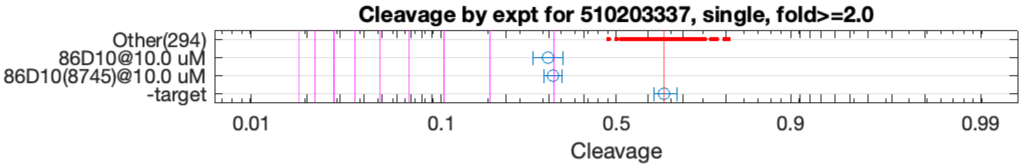

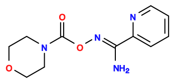

86D10

v>1.6 s=2.1

510218739 GCTGTC ACTGGA CTTTCTCGGAGGTTTATTCGGGAAGGCGGA TCCGGT CTGATGA ATCCGTGGCGGGAC GAAACAGC

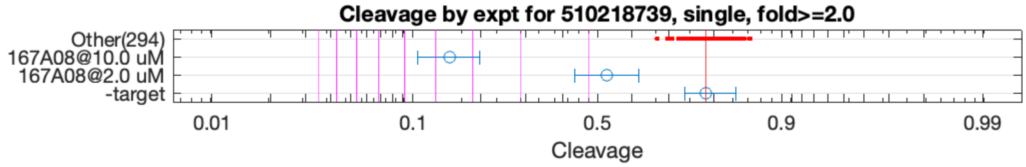

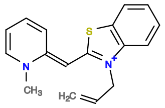

167A08

v>1.3 s=4.6

510245864 GCTGTC ACTGGA AAACCGAACGATTGATGGACGGACACCTC TCCGGT CTGACGA GTCC GATGTGTCTGCGTGTTGGGTG GGAC GAAACAGC

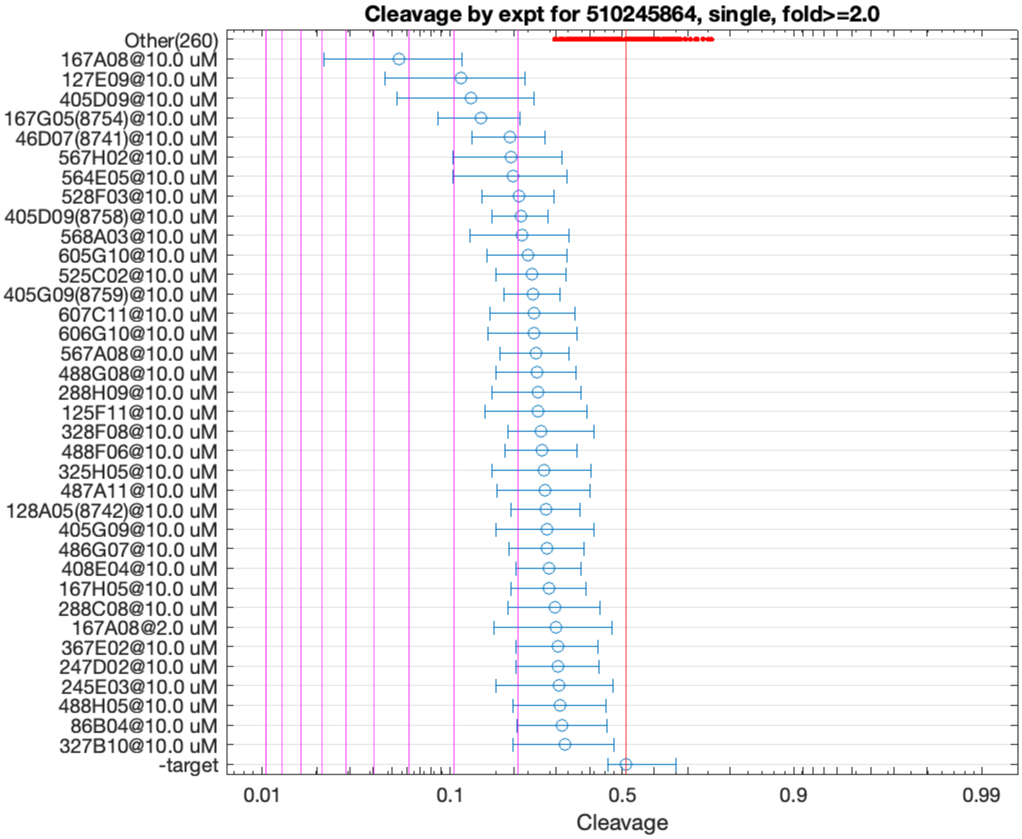

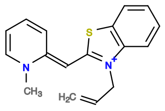

167A08

v>1.8 s=4.3

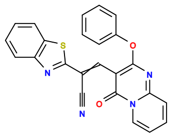

127E09

v>1.5 s=2.8

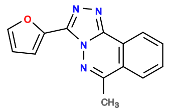

405D09

v>1.5 s=2.8

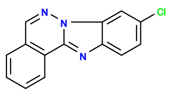

167G05

v>1.6 s=2.5

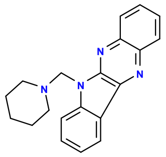

46D07

v>1.4 s=2.1

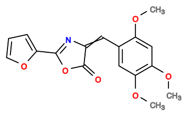

564E05

v>1.6 s=2.1

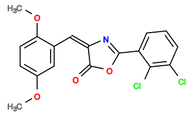

567H02

v>1.2 s=2.0

510255226 GCTGTC ACTGGA CTAGA TTCGGT CTGAGGA ACTGTGCGACGCAGC GAAACAGC ATTAGTGCGGGACGAAACAGC

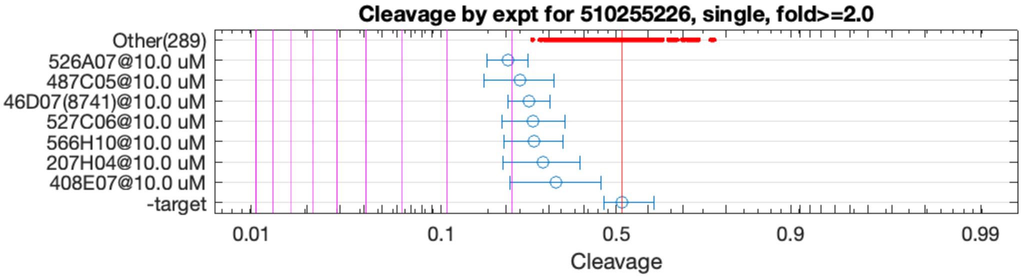

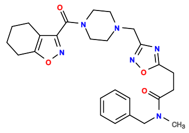

526A07

v>1.1 s=2.0

510318329 GCTGTC ACTGG ATGCATGACCATATTTGGTGTGCCGAGCGGCA CCAGT CTGATGA GTTC GTGGCG GGAC GAAACAGC

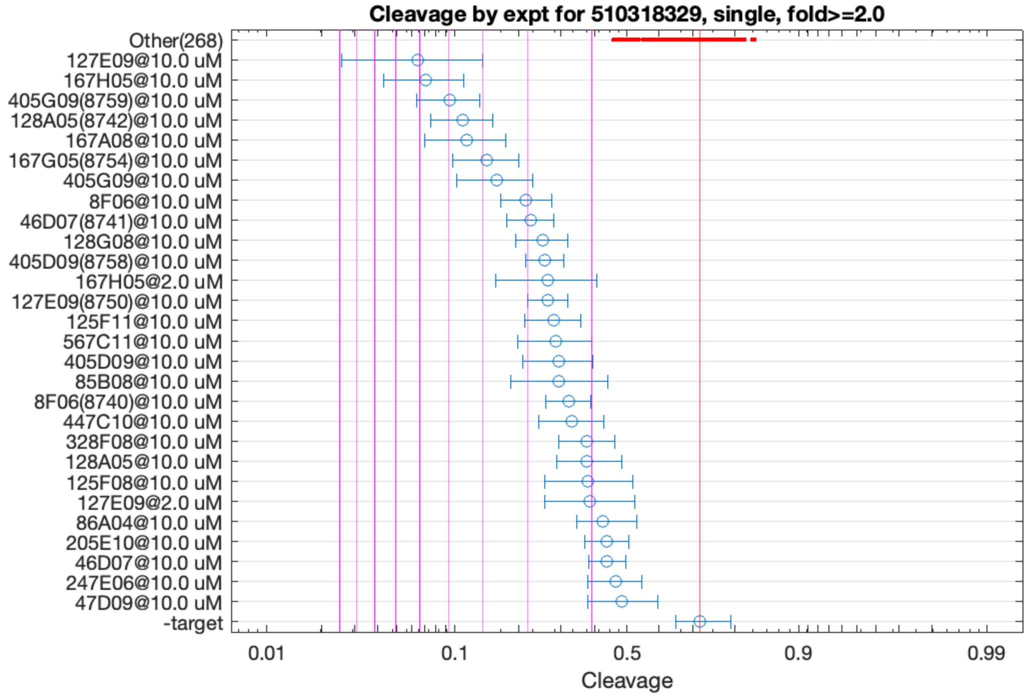

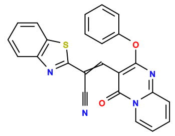

127E09

v>2.6 s=6.2

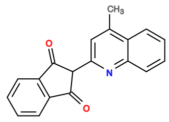

167H05

v>1.4 s=5.8

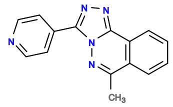

405G09

v>3.0 s=4.9

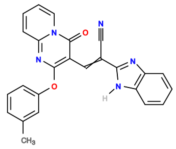

128A05

v>2.2 s=4.5

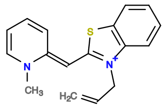

167A08

v>2.6 s=4.5

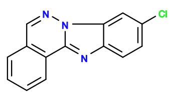

167G05

v>2.6 s=3.9

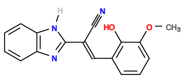

8F06

v>2.2 s=3.0

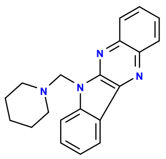

46D07

v>2.2 s=2.9

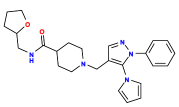

446C03

v>2.9

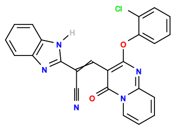

128G08

v>2.9 s=2.8

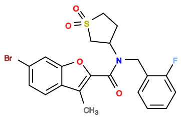

408A05

v>2.8

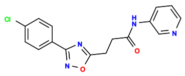

405A06

v>2.7

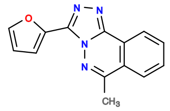

405D09

v>2.4 s=2.7

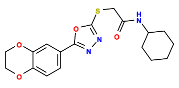

246A07

v>2.7

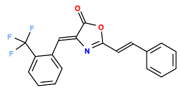

566D10

v>2.7

510337926 GCTGTC ACTGG ATTTTCGTG TCAGT CTGATGA GTTCT TGTTGACGAAACAGCAGAGTGCCC GGGAC GAAACAGC

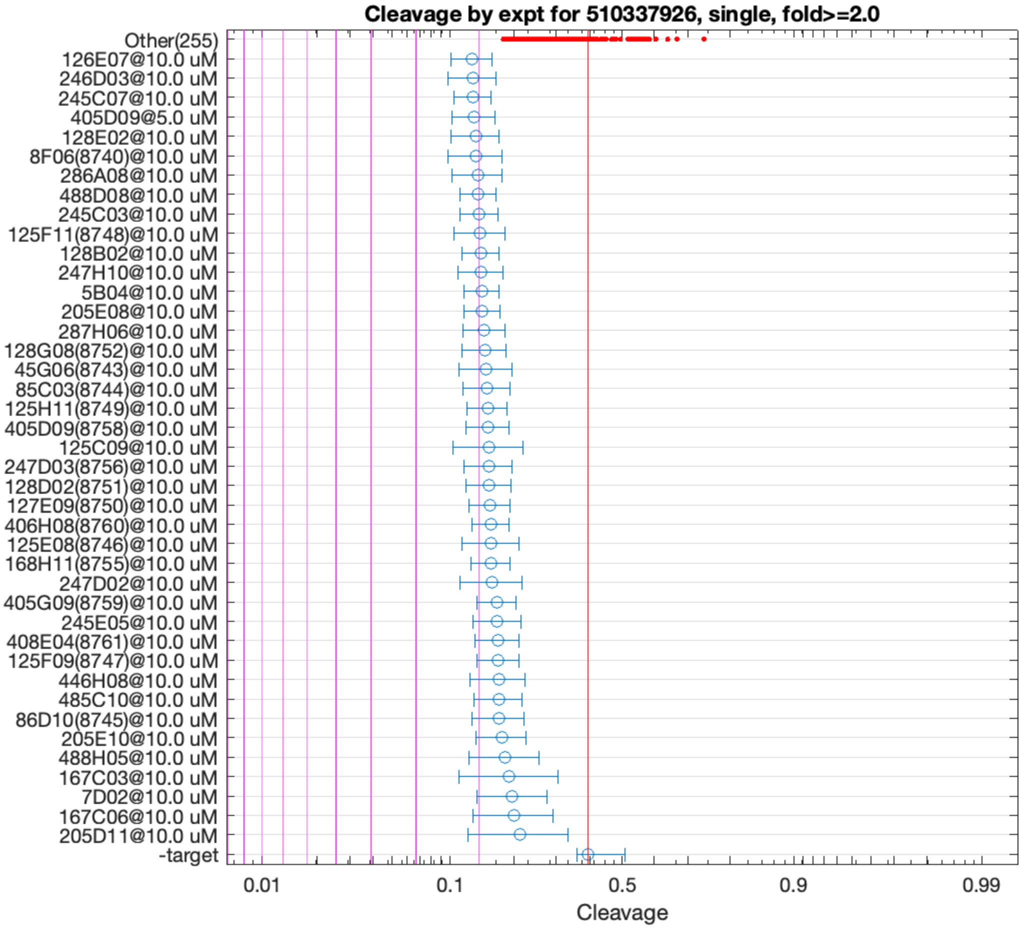

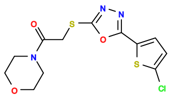

246D03

v>0.9 s=2.1

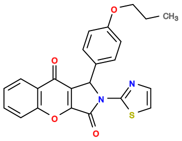

126E07

v>1.0 s=2.1

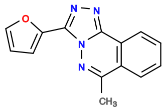

405D09

v>1.0 s=2.1

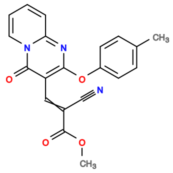

128E02

v>0.9 s=2.1

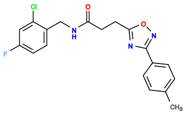

245C07

v>0.9 s=2.1

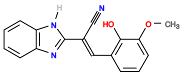

8F06

v>1.0 s=2.1

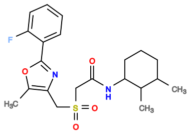

286A08

v>0.7 s=2.1

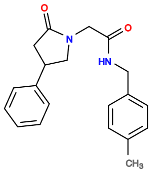

247H10

v>0.9 s=2.0

510463768 GCTGTC ACTGGA TTTCCAAGATGATGGACAGACACCTC TCCGGT CTGATGA GTCC GATGTGTCTGCGAATGTCTGCGTGTTGGTG GGAC GAAACAGC

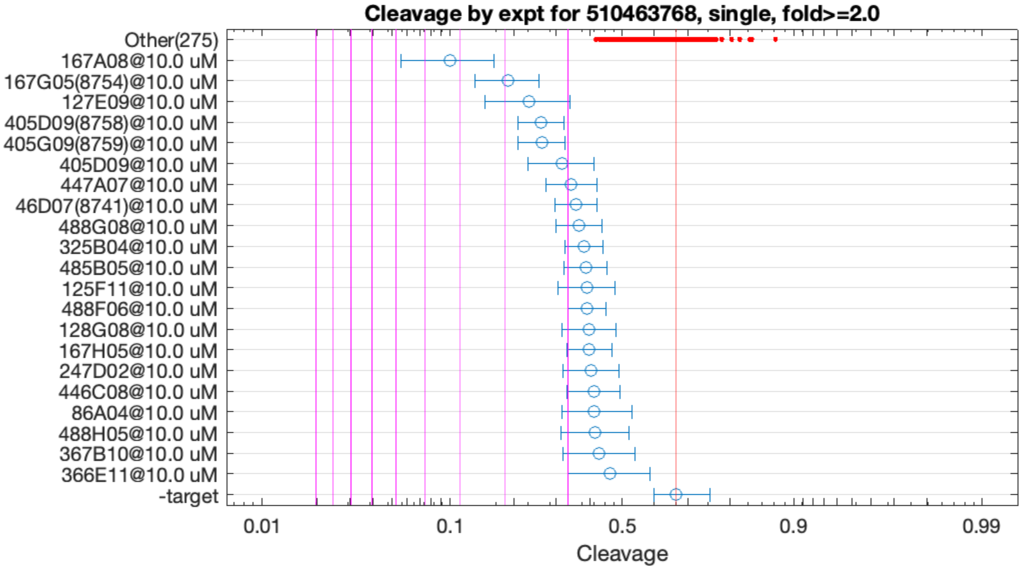

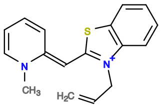

167A08

v>1.6 s=4.3

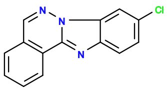

167G05

v>1.3 s=2.9

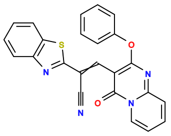

127E09

v>1.3 s=2.5

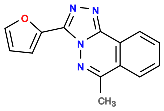

405D09

v>1.5 s=2.4

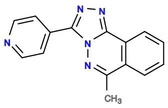

405G09

v>1.4 s=2.4

511084246 GCTGTC ACTGGA AACGAAGG TCTAGT CTGAAGA GTCC GCCTAAGCGCTGCTCGCACAGTGGCTCGTG GGAC GAAACAGC

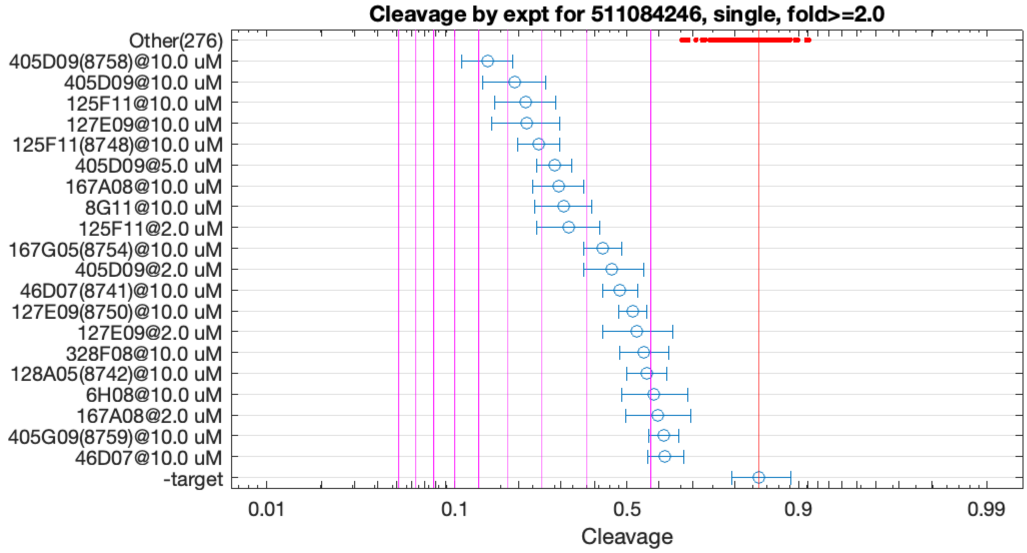

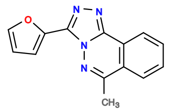

405D09

v>4.3 s=5.6

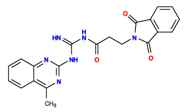

125F11

v>2.0 s=4.4

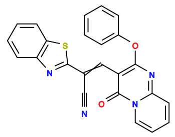

127E09

v>2.4 s=4.3

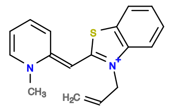

167A08

v>2.4 s=3.6

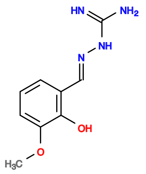

8G11

v>3.5 s=3.5

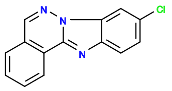

167G05

v>2.0 s=2.7

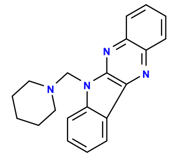

46D07

v>2.2 s=2.4

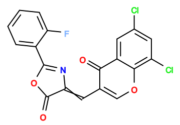

564E04

v>2.4

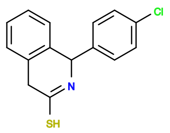

168A06

v>2.3

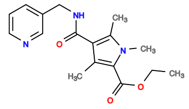

326A05

v>2.3

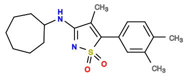

446G09

v>2.3

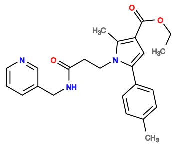

325C10

v>2.3

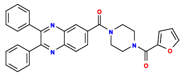

568D05

v>2.2

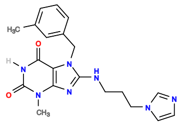

88D02

v>2.2

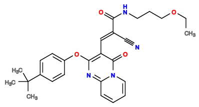

127C11

v>2.2

511447013 GCTGTC AC TGGAGATGAGTCC GT CTGATGA GCGC TTAGCC GCGT GAAACAGC CTACCTGCCCGGACGAAACAGC

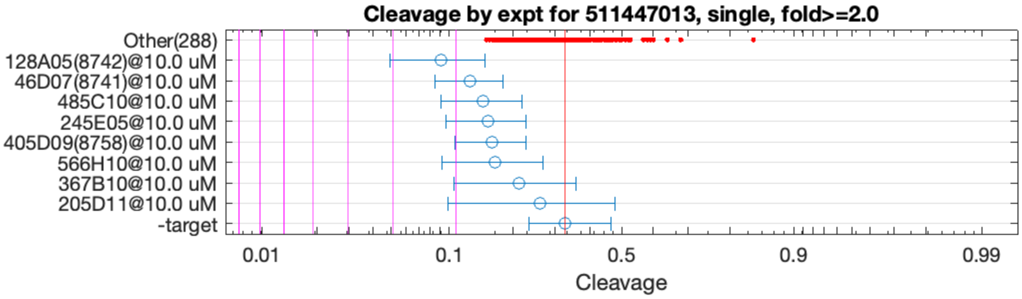

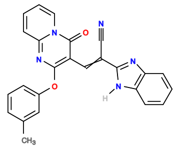

128A05

v>0.6 s=2.3

512101813 GCTGTC ACTGG ATGCATGACCATATTTGGTGTGCCGAGCGGCA CCAGT CTGACGA GTCC GTGGCG GGAC GAAACAGC

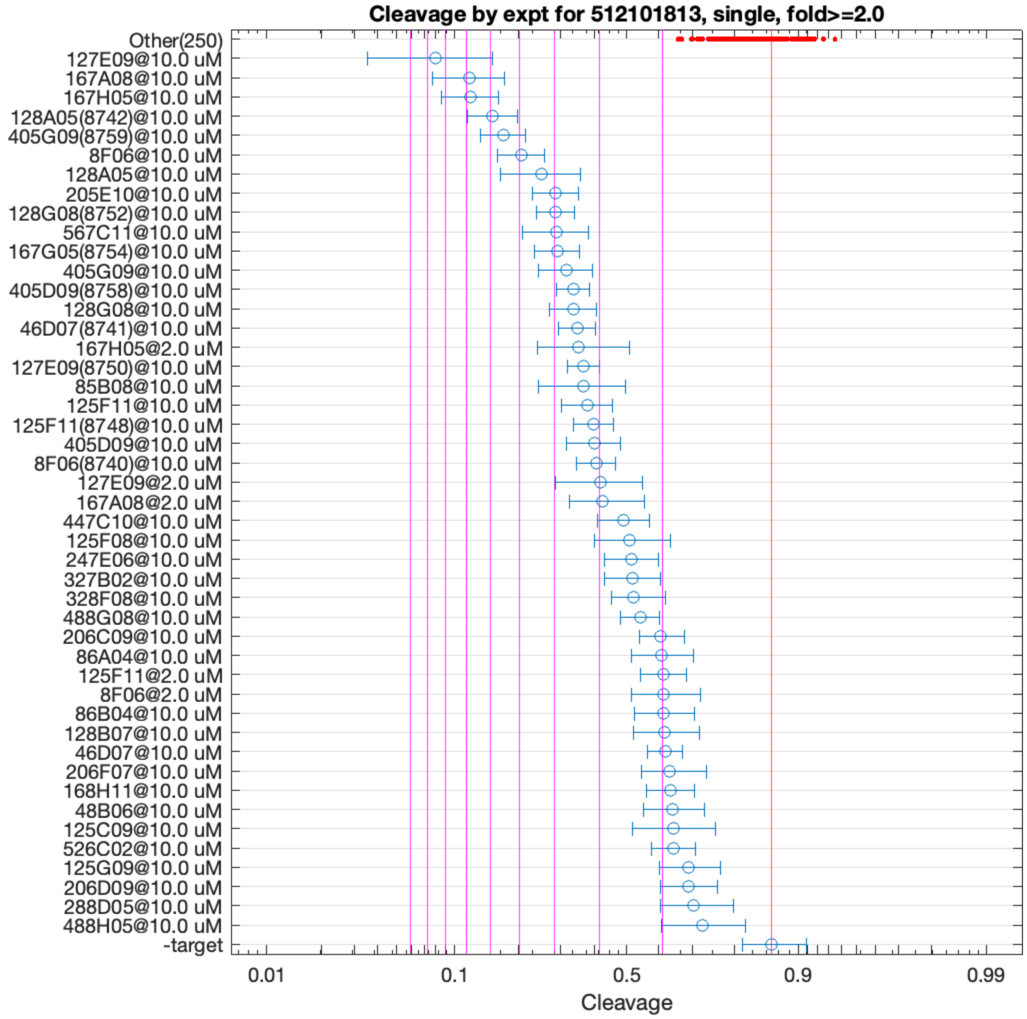

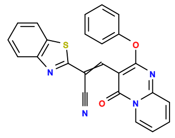

127E09

v>3.5 s=8.7

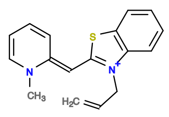

167A08

v>3.0 s=7.0

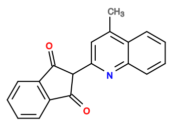

167H05

v>3.5 s=6.9

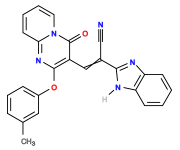

128A05

v>3.1 s=5.9

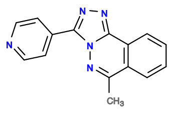

405G09

v>3.9 s=5.6

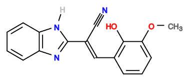

8F06

v>2.5 s=4.9

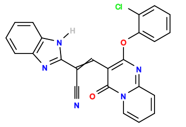

128G08

v>3.3 s=3.9

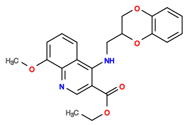

205E10

v>2.8 s=3.9

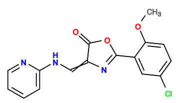

567C11

v>2.7 s=3.9

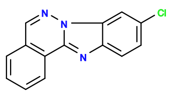

167G05

v>3.0 s=3.9

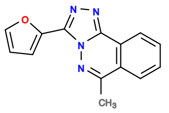

405D09

v>2.6 s=3.5

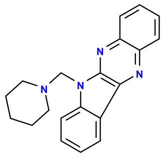

46D07

v>2.6 s=3.5

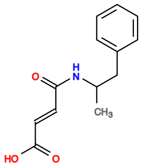

6E06

v>3.4

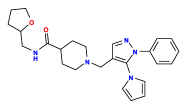

446C03

v>3.4

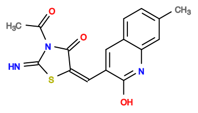

85B08

v>2.9 s=3.3

512112258 GCTGTC ACTGGA AACGAAGG TCTAGT CTGAAGA GTCC GCCTAAGCGCTGCTCACACAGTGTCTCGTG GGAC GAAACAGC

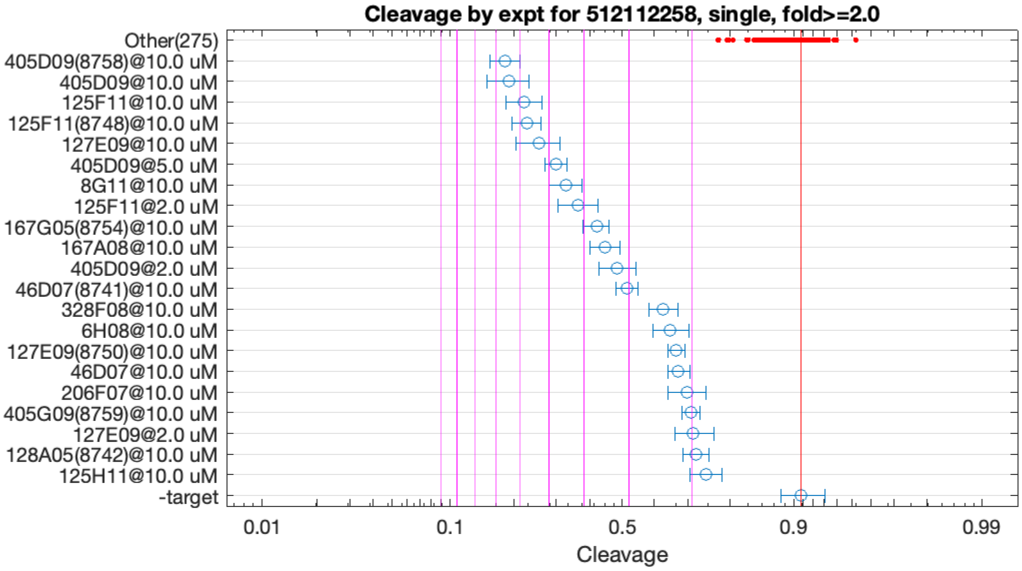

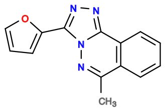

405D09

v>6.4 s=6.7

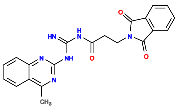

125F11

v>2.7 s=5.8

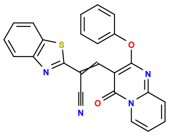

127E09

v>3.0 s=5.3

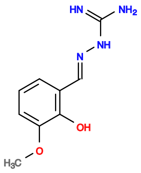

8G11

v>5.1 s=4.5

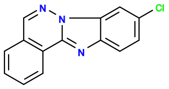

167G05

v>2.8 s=3.6

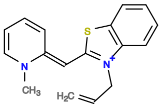

167A08

v>2.9 s=3.5

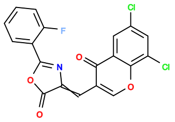

564E04

v>3.2

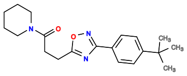

405A02

v>3.1

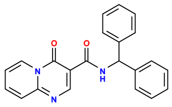

325F04

v>3.1

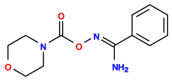

86H09

v>3.0

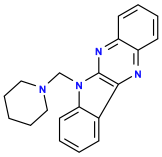

46D07

v>3.1 s=3.0

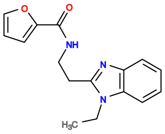

408C11

v>3.0

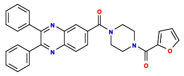

568D05

v>2.9

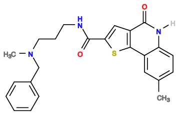

328F08

v>2.5 s=2.4

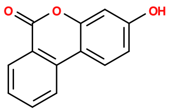

6H08

v>3.8 s=2.3

512117004 GCTGTC ACTGGA GGGGCAAG TCCGGT CTGACGA GTCCT GATAACGAGCCTATTGGCGCACCCGGG GGGAC GAAACAGC

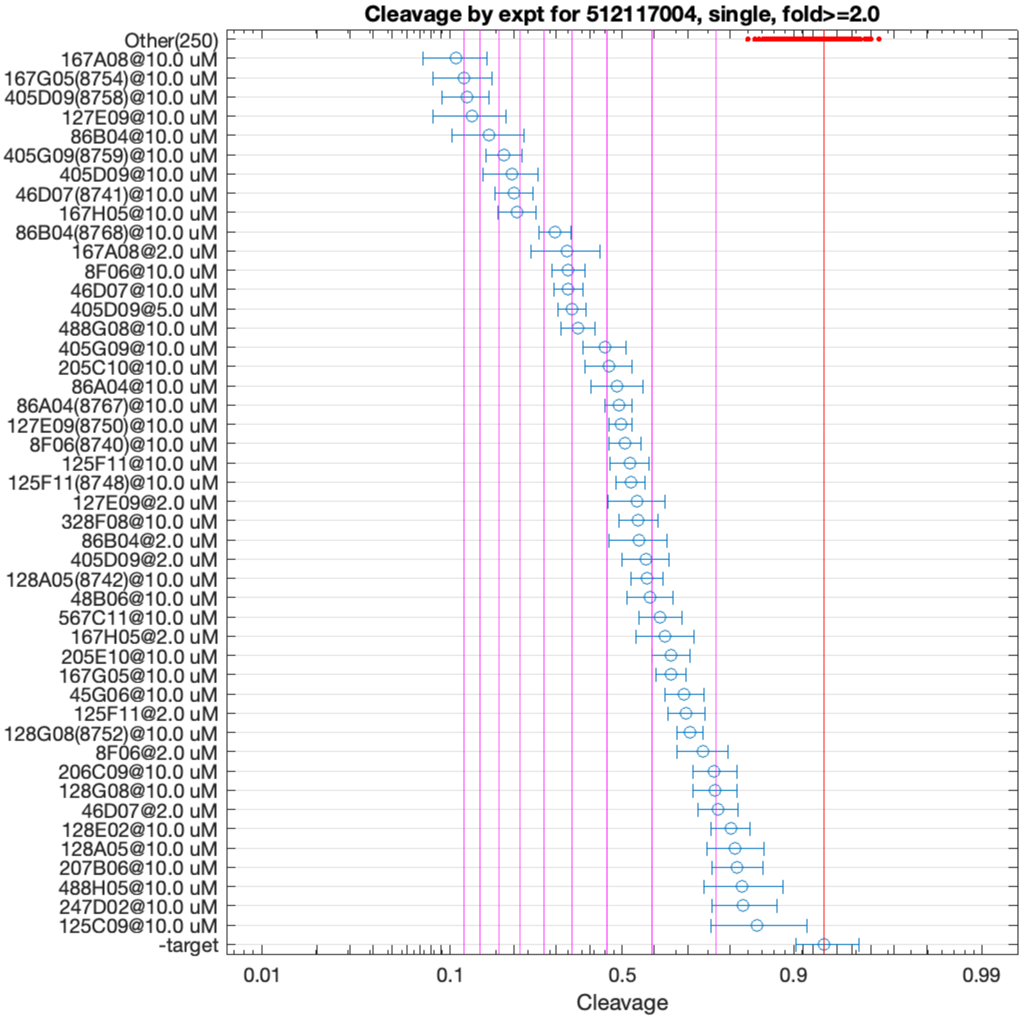

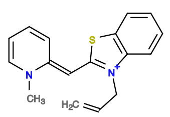

167A08

v>8.0 s=10.2

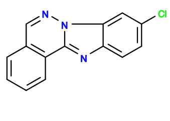

167G05

v>7.5 s=10.1

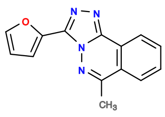

405D09

v>7.4 s=9.6

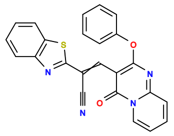

127E09

v>5.1 s=9.6

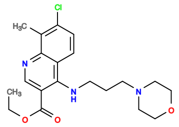

86B04

v>6.3 s=8.5

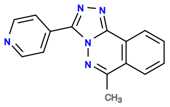

405G09

v>6.4 s=7.9

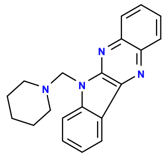

46D07

v>6.6 s=7.3

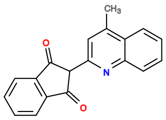

167H05

v>3.8 s=7.1

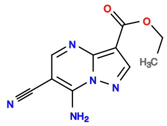

205E05

v>6.1

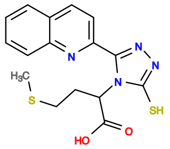

168F07

v>6.0

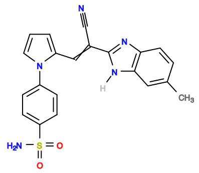

46B07

v>5.8

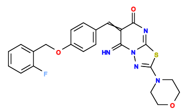

327H06

v>5.8

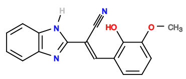

8F06

v>3.9 s=5.2

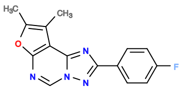

488G08

v>4.2 s=4.8

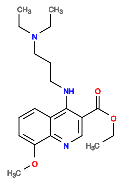

205C10

v>6.4 s=3.9

512119028 GCTGTC ACTGG ATGCATGACCATATTTGGTGTGCCGAGCGGCA CCAGT CTGACGA GTCT GTGGCG GGAC GAAACAGC

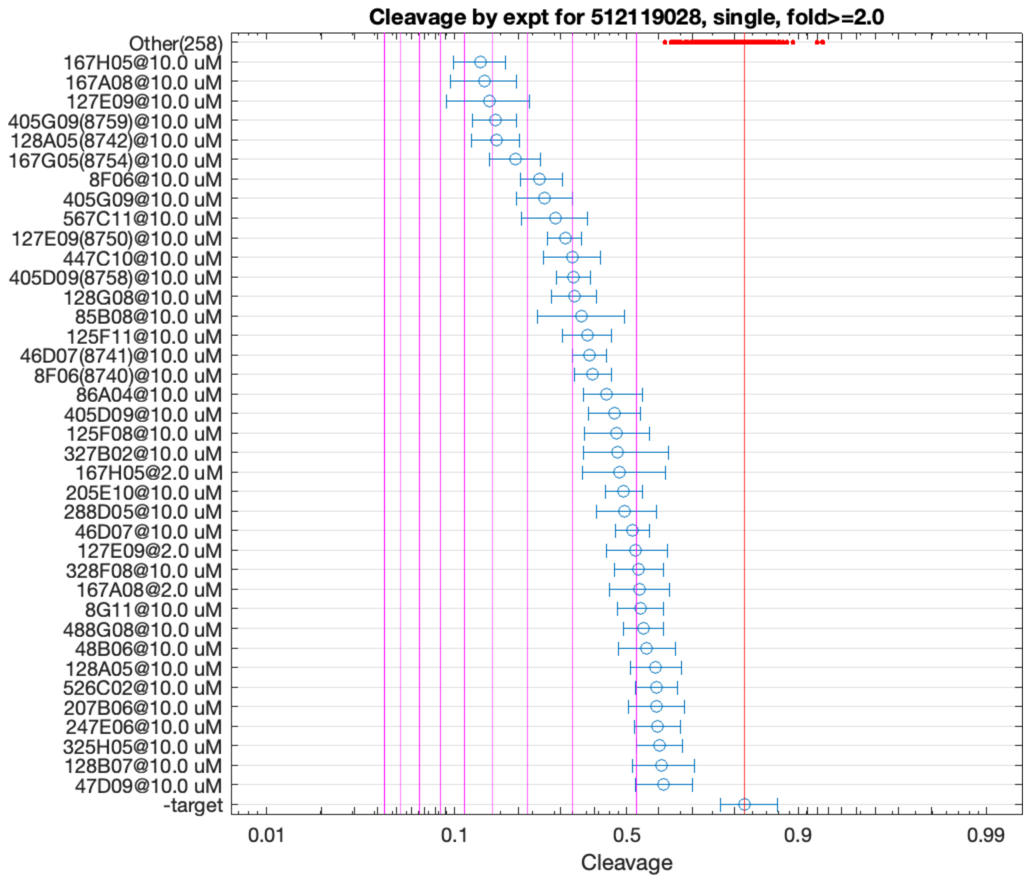

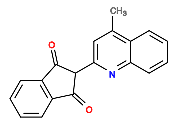

167H05

v>1.7 s=5.5

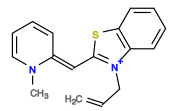

167A08

v>2.9 s=5.3

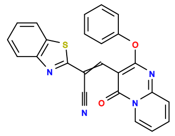

127E09

v>3.0 s=5.1

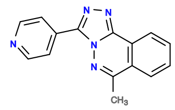

405G09

v>3.6 s=4.9

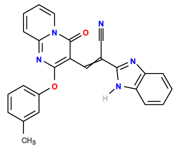

128A05

v>2.4 s=4.9

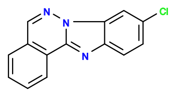

167G05

v>3.0 s=4.3

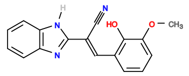

8F06

v>2.3 s=3.7

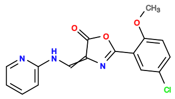

567C11

v>2.5 s=3.4

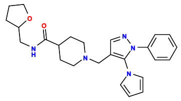

446C03

v>3.1

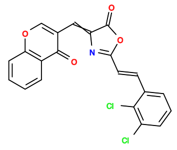

567D05

v>3.1

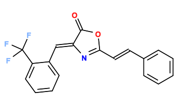

566D10

v>3.0

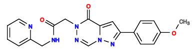

447C10

v>2.9 s=3.0

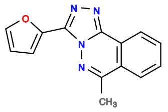

405D09

v>2.4 s=3.0

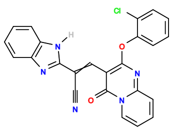

128G08

v>3.3 s=3.0

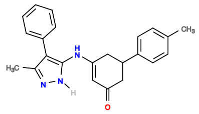

168E02

v>2.9

512119032 GCTGTC ACTGGA ATTATGCCTTTCGAAGGGTTGCCGATCTAA TCTGGT CTGAAGA GTCT GTGGCG GGAC GAAACAGC

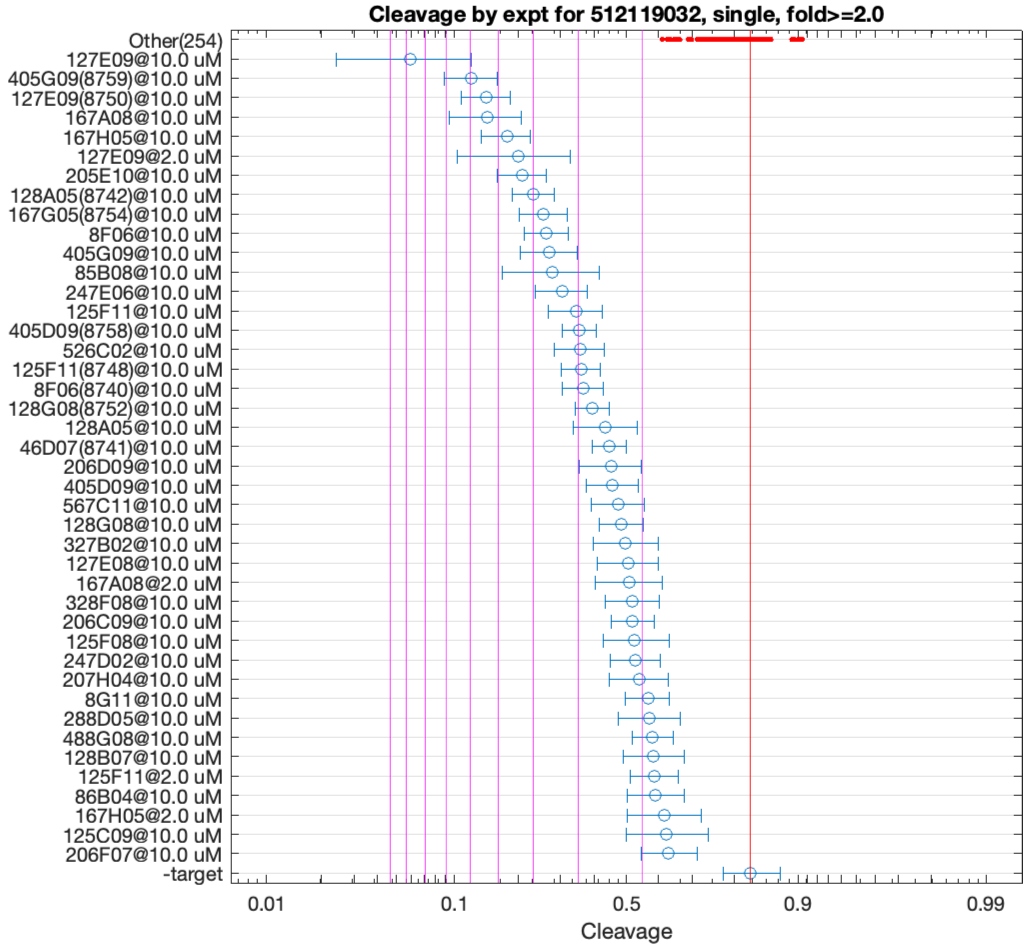

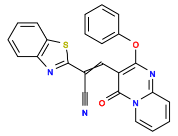

127E09

v>3.5 s=8.7

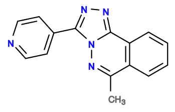

405G09

v>3.6 s=6.0

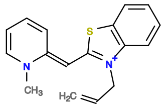

167A08

v>2.7 s=5.3

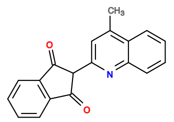

167H05

v>2.7 s=4.7

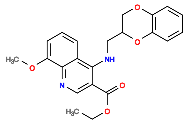

205E10

v>2.8 s=4.3

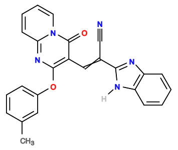

128A05

v>2.4 s=4.0

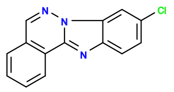

167G05

v>2.9 s=3.8

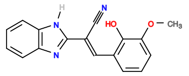

8F06

v>2.3 s=3.7

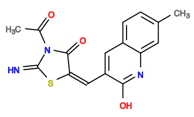

85B08

v>2.7 s=3.5

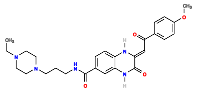

247E06

v>2.6 s=3.3

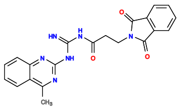

125F11

v>2.2 s=3.1

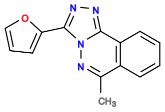

405D09

v>2.5 s=3.0

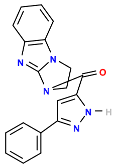

526C02

v>2.9 s=3.0

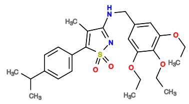

447C04

v>2.9

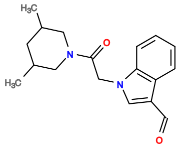

606E08

v>2.9

512119326 GCTGTC ACTGG ATGCATGACCATATTTGGTGTGCCGAGCGGCA CCAGT CTGACGA GTTC GTGGCG GGAC GAAACAGC

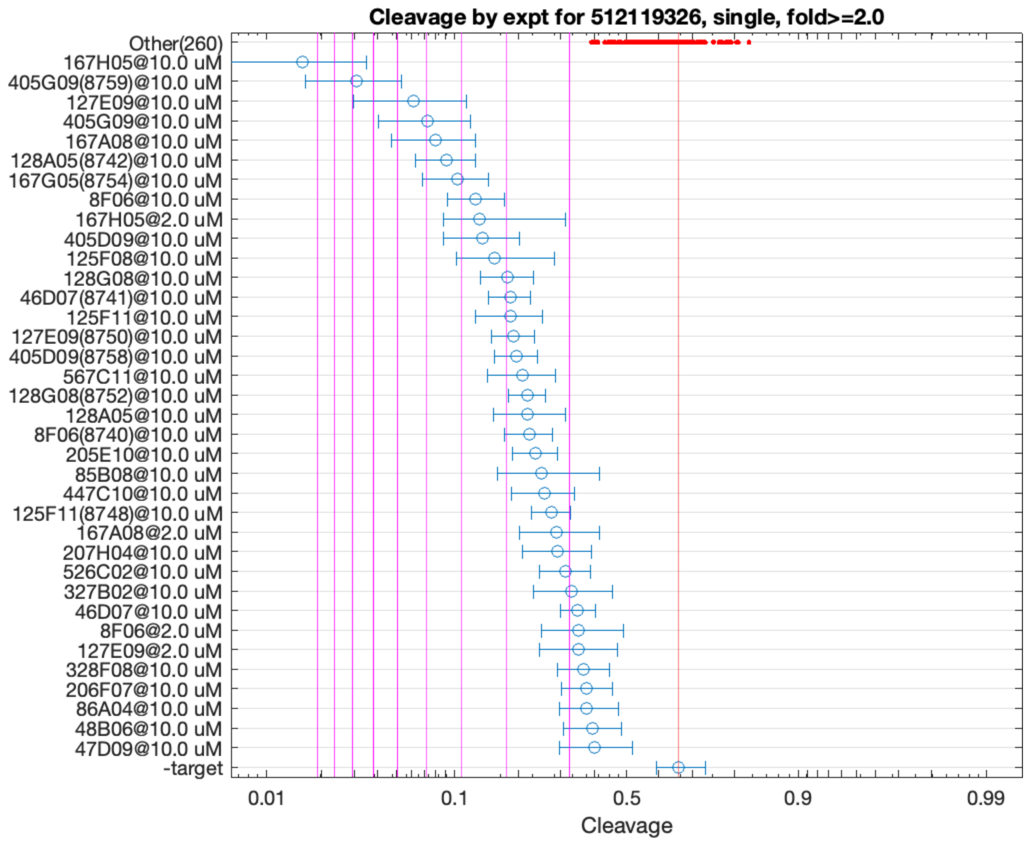

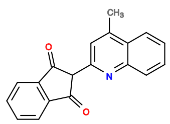

167H05

v>2.3 s=11.3

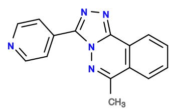

405G09

v>3.2 s=7.7

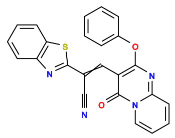

127E09

v>3.0 s=5.4

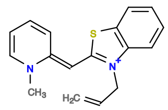

167A08

v>2.9 s=4.8

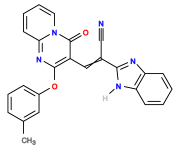

128A05

v>2.5 s=4.4

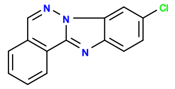

167G05

v>3.1 s=4.2

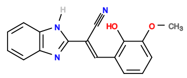

8F06

v>2.3 s=3.6

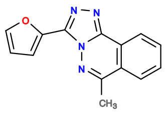

405D09

v>2.4 s=3.5

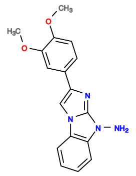

125F08

v>2.4 s=3.2

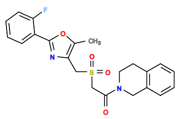

286H08

v>3.0

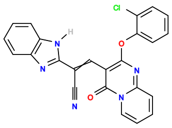

128G08

v>3.1 s=3.0

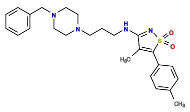

446E08

v>2.9

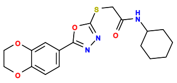

246A07

v>2.9

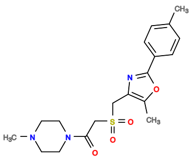

285A10

v>2.9

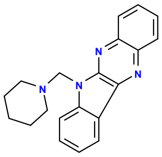

46D07

v>2.6 s=2.9

512158904 GCTGTC ACTGGAG AAACTGCACATCGTGTAGTGGAGGCAAC TTCCGGT CTGAAGA GTCC TAGAGCCT GGAC GAAACAGC

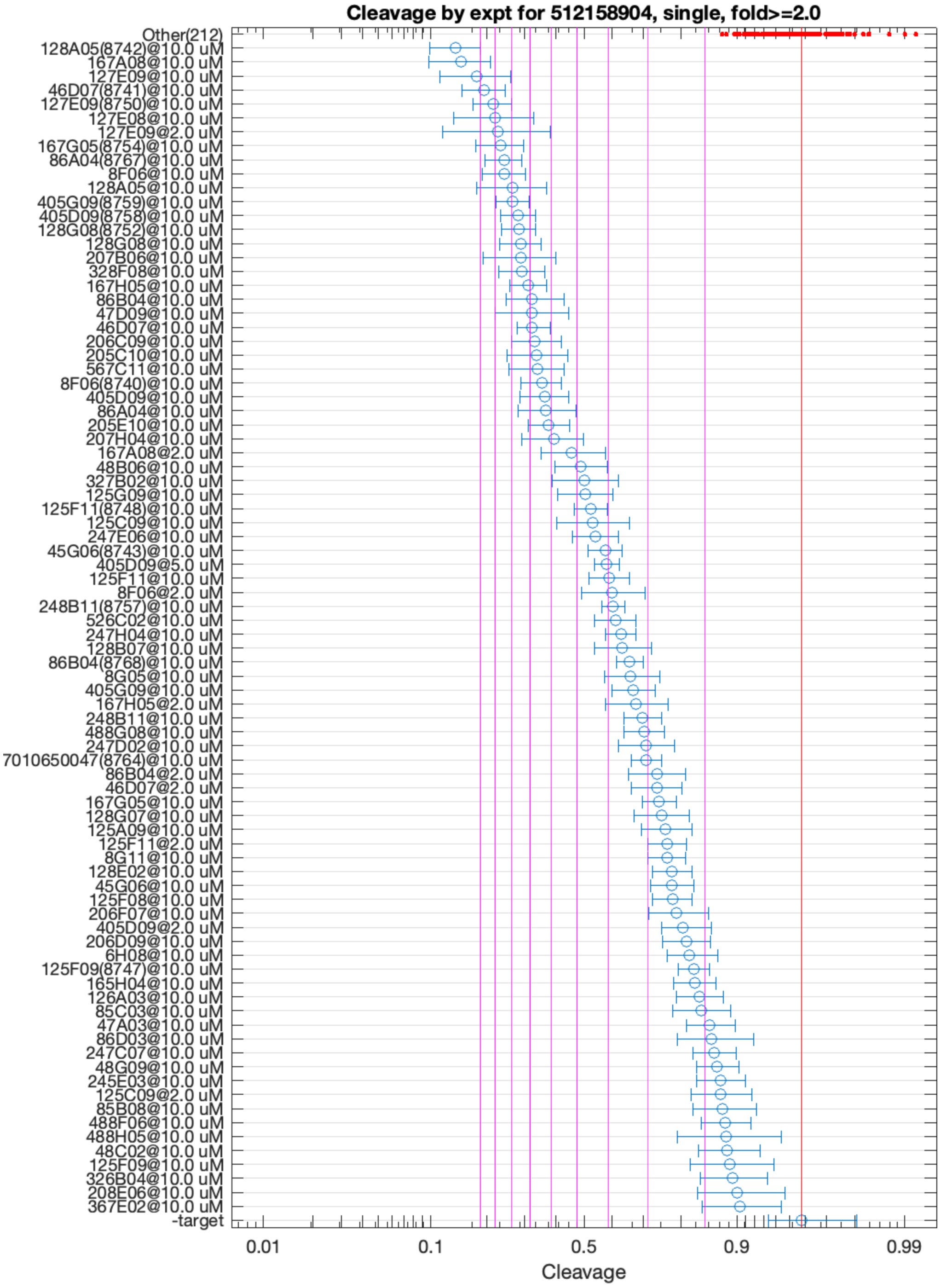

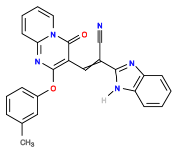

128A05

v>6.9 s=11.8

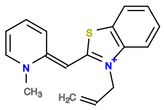

167A08

v>7.4 s=11.5

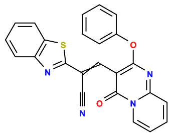

127E09

v>8.6 s=10.4

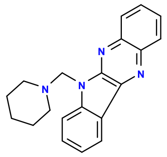

46D07

v>7.1 s=9.8

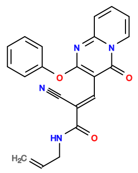

127E08

v>8.8 s=8.9

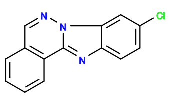

167G05

v>7.4 s=8.8

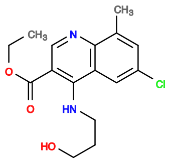

86A04

v>6.0 s=8.7

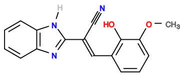

8F06

v>6.1 s=8.5

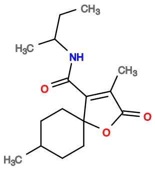

286D11

v>8.3

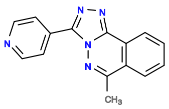

405G09

v>6.3 s=8.1

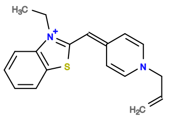

167E07

v>8.1

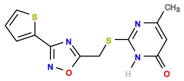

168G03

v>8.0

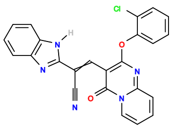

128G08

v>7.7 s=7.8

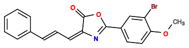

567A09

v>7.7

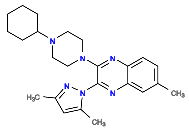

248E02

v>7.7

512213078 GCTGTC ACTGGA TGCATGACCTATTTGGTGTGCTGAGCTGC TCTGGT CTGACGA GTCC GTGGCG GGAC GAAACAGC

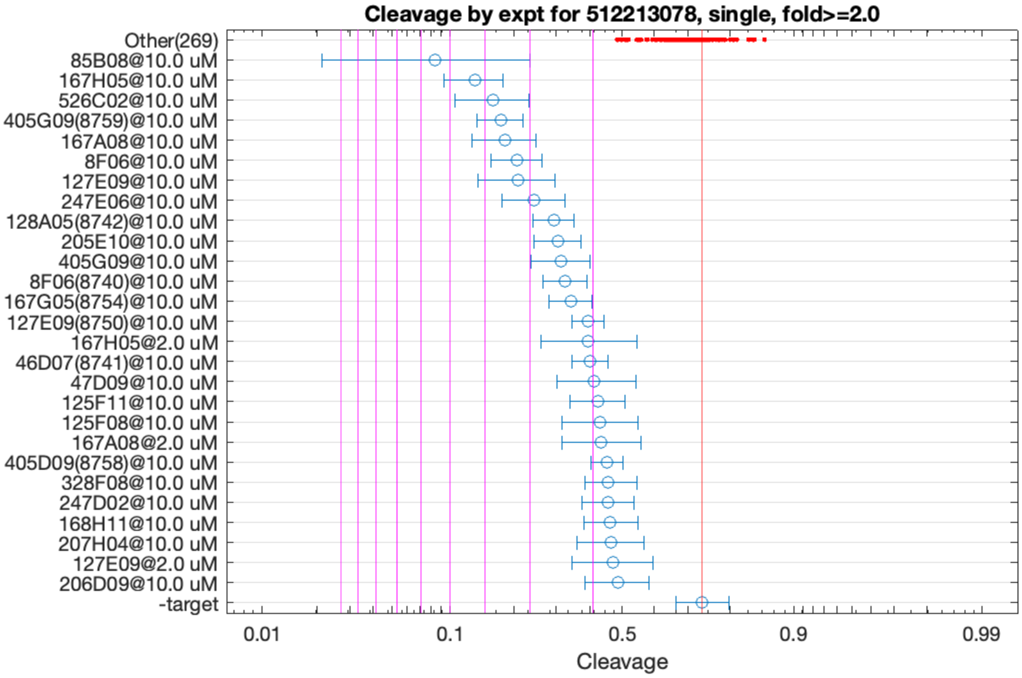

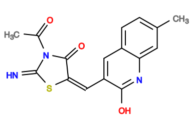

85B08

v>2.7 s=5.6

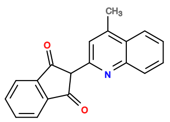

167H05

v>2.1 s=4.3

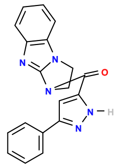

526C02

v>2.7 s=3.8

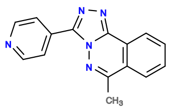

405G09

v>2.5 s=3.6

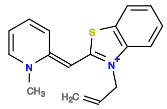

167A08

v>2.2 s=3.5

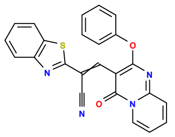

127E09

v>2.2 s=3.3

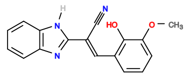

8F06

v>1.9 s=3.2

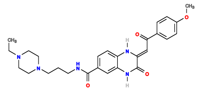

247E06

v>2.2 s=2.9

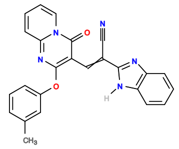

128A05

v>1.9 s=2.6

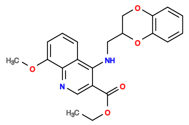

205E10

v>2.0 s=2.5

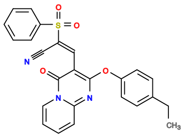

128G06

v>2.5

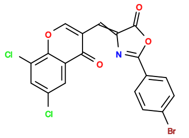

564H11

v>2.5

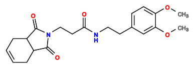

205G07

v>2.5

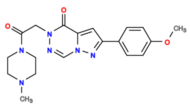

447E09

v>2.4

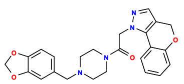

328G11

v>2.4

512213338 GCTGTC ACTGGA TGGAGGCT TCTAGT CTGACGA GTCCTG CAACAACCCAGTTGCACGCAAGCC CGGGAC GAAACAGC

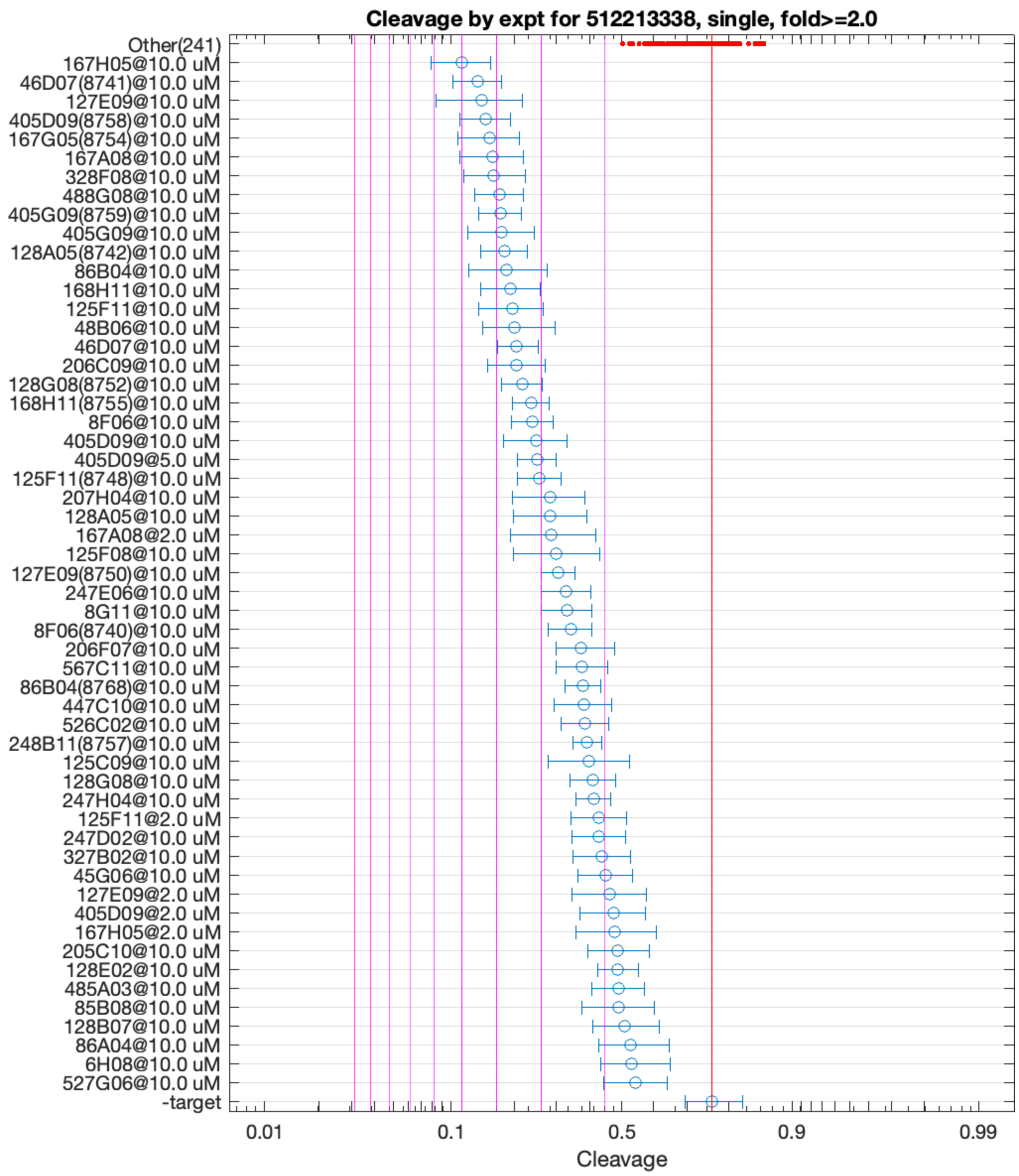

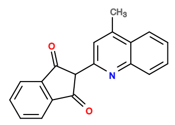

167H05

v>3.1 s=5.1

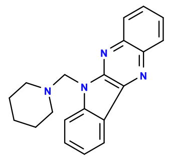

46D07

v>3.1 s=4.5

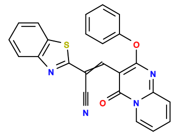

127E09

v>3.0 s=4.4

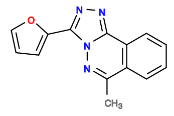

405D09

v>3.4 s=4.4

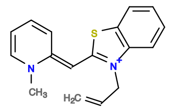

167A08

v>3.1 s=4.1

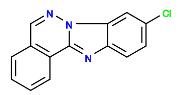

167G05

v>3.3 s=4.1

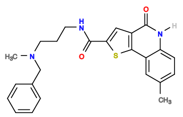

328F08

v>3.4 s=4.0

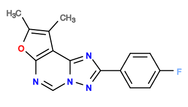

488G08

v>3.0 s=4.0

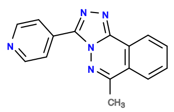

405G09

v>3.0 s=4.0

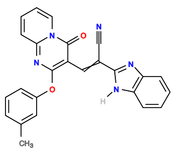

128A05

v>2.8 s=3.8

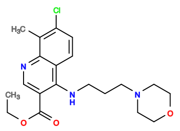

86B04

v>3.2 s=3.8

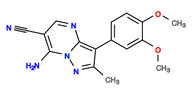

168H11

v>3.2 s=3.7

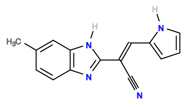

48B06

v>3.3 s=3.6

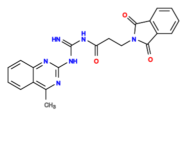

125F11

v>2.7 s=3.6

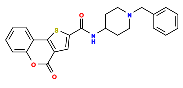

206C09

v>2.9 s=3.5

512228786 GCTGTC ACTGGA AACGAAGG TCTAGT CTGAAGA GTCC GCCTAAGCGCTGCTCACACAGTGACTCGTG GGAC GAAACAGC

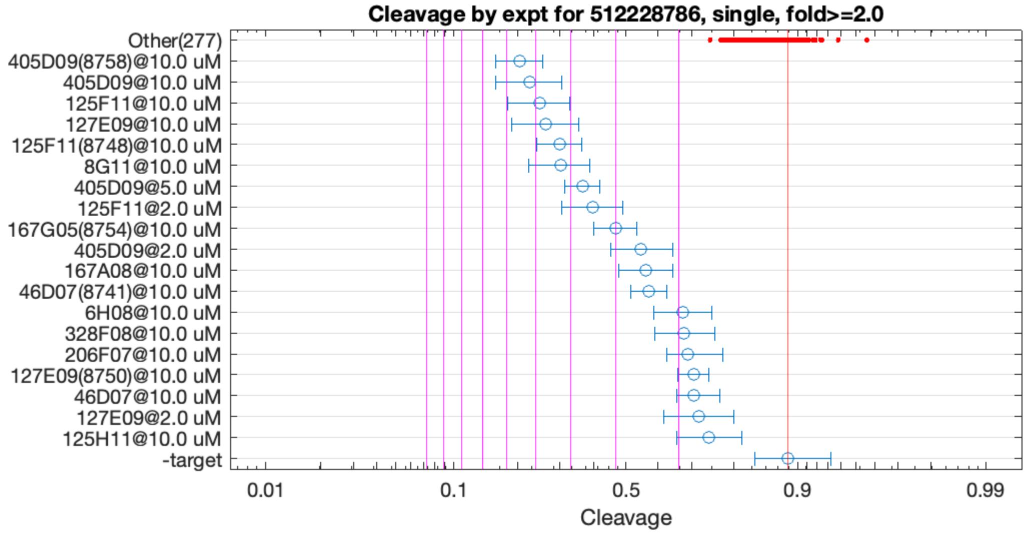

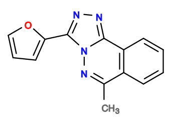

405D09

v>4.7 s=5.5

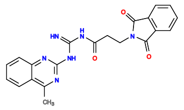

125F11

v>2.0 s=4.8

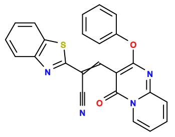

127E09

v>2.2 s=4.6

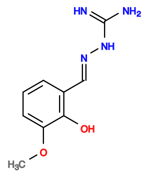

8G11

v>3.7 s=4.2

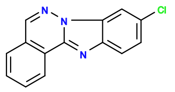

167G05

v>2.1 s=3.0

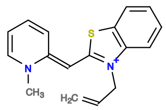

167A08

v>2.0 s=2.5

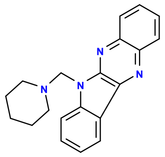

46D07

v>2.3 s=2.4

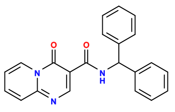

325F04

v>2.2

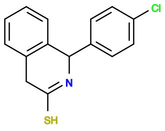

168A06

v>2.2

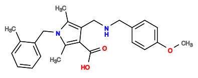

365C08

v>2.2

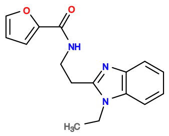

408C11

v>2.2

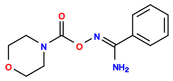

86H09

v>2.1

512257380 GCTGTC ACTG GATGGGAAA CAGT CTGATGA GTCC TGCGATGCCTGATACACATGCCTCTCCGC GGAC GAAACAGC

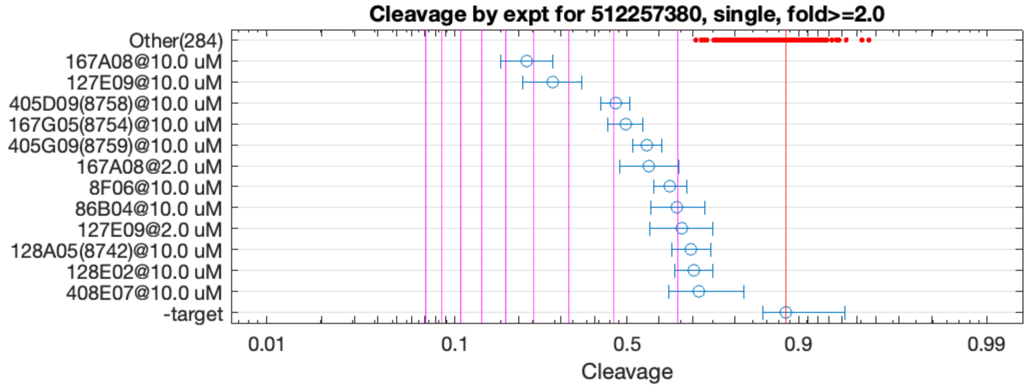

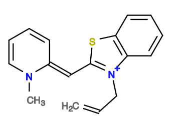

167A08

v>2.9 s=5.3

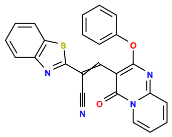

127E09

v>2.4 s=4.4

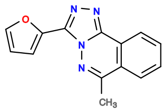

405D09

v>2.4 s=3.0

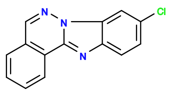

167G05

v>2.4 s=2.8

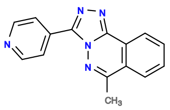

405G09

v>2.3 s=2.4

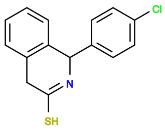

168A06

v>2.3

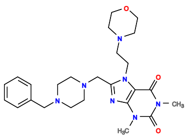

166C04

v>2.2

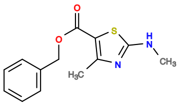

86F10

v>2.2

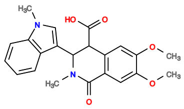

207A03

v>2.2

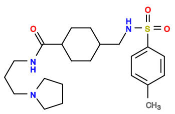

606A04

v>2.2

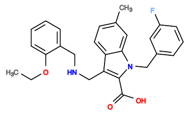

527D04

v>2.2

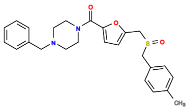

487A06

v>2.1

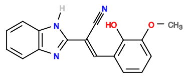

8F06

v>1.9 s=2.1

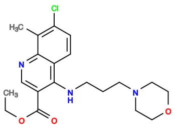

86B04

v>2.3 s=2.0

512293240 GCTGTC ACTGGA GAACACG TTCAGT CTGATGA GTCC ACTGCCTCTGCCCTATCTGCAAGCGTACCG GGAC GAAACAGC

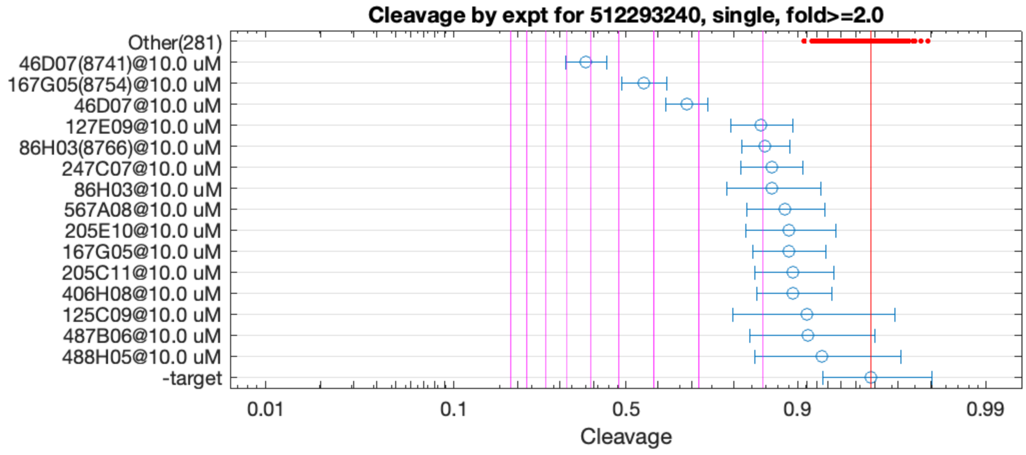

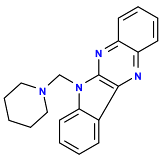

46D07

v>2.1 s=6.2

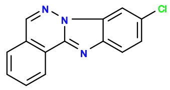

167G05

v>1.2 s=4.2

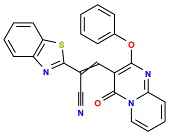

127E09

v>1.0 s=2.0

512295690 GCTGTC ACTGGA GGCAGGA TCTGGT CTGATGA GTCC TCGATGTGGTCGCAATTCCGCATACGTGCC GGAC GAAACAGC

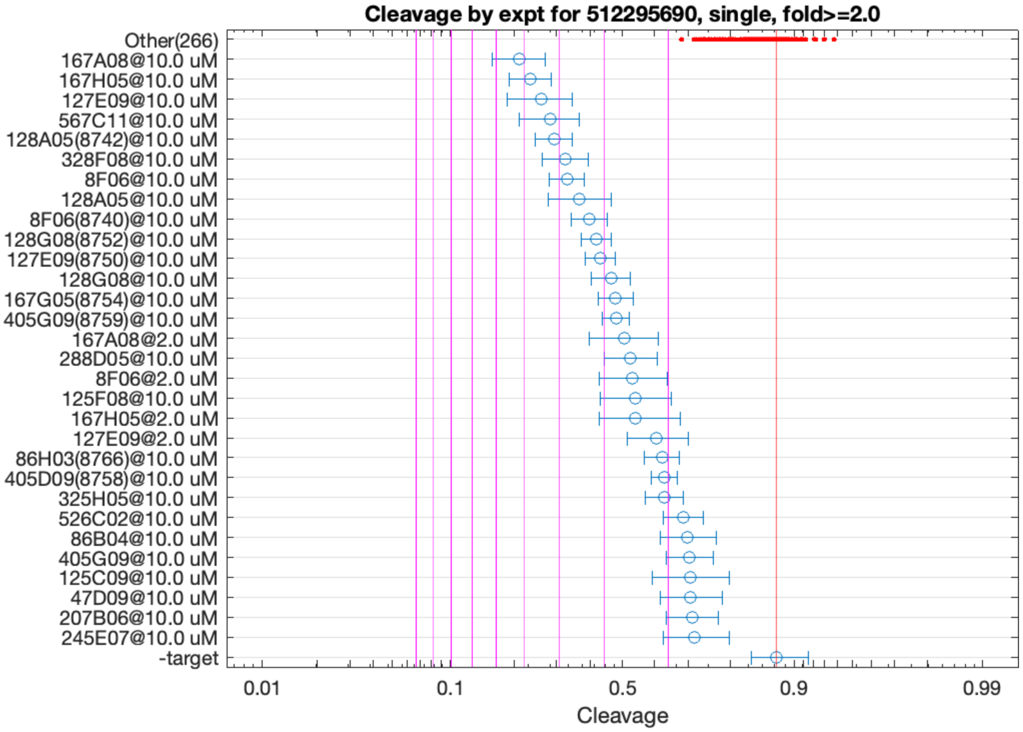

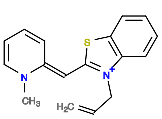

167A08

v>3.4 s=5.3

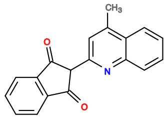

167H05

v>3.3 s=4.8

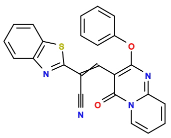

127E09

v>2.9 s=4.5

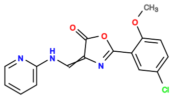

567C11

v>3.4 s=4.3

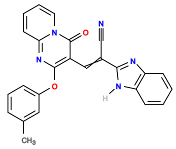

128A05

v>2.8 s=4.1

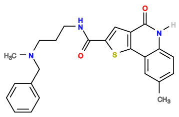

328F08

v>3.4 s=3.8

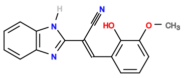

8F06

v>3.0 s=3.8

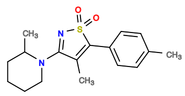

446H05

v>3.7

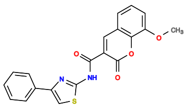

568H09

v>3.7

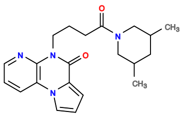

485H06

v>3.6

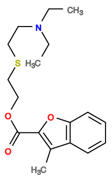

48G05

v>3.5

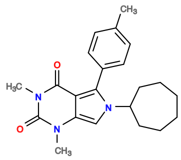

408D06

v>3.5

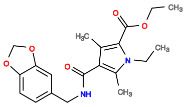

326G06

v>3.5

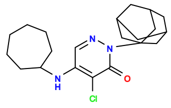

247D10

v>3.5

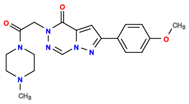

447E09

v>3.5

512298529 GCTGTC ACTGGAT CGGACA ATCTAGT CTGAAGA GTCC TGCGATGCCTGATACACATGCCTCTCCGC GGAC GAAACAGC

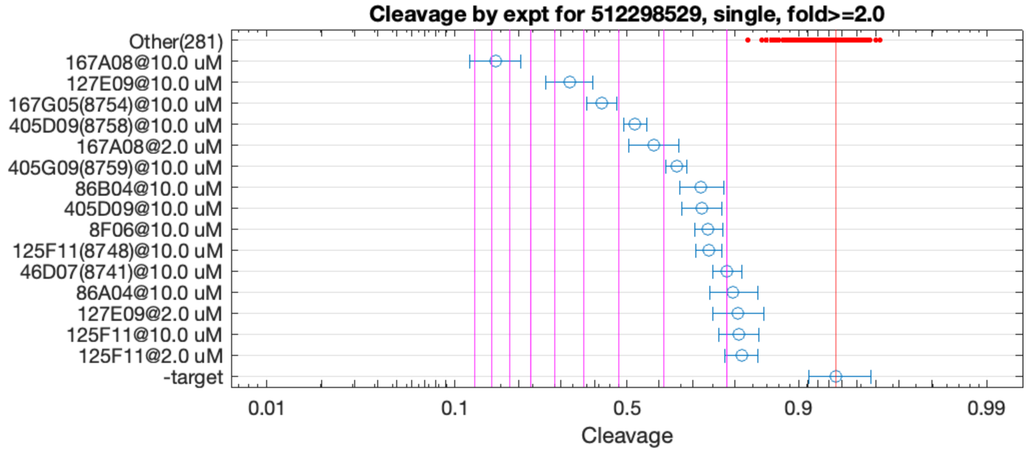

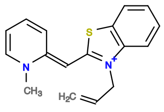

167A08

v>3.0 s=8.7

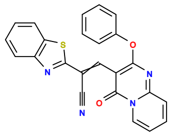

127E09

v>2.0 s=5.4

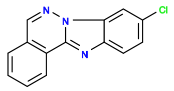

167G05

v>2.2 s=4.5

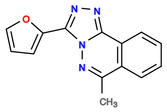

405D09

v>2.2 s=3.5

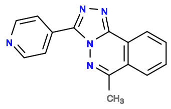

405G09

v>2.1 s=2.8

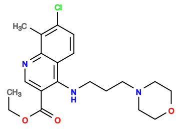

86B04

v>1.7 s=2.4

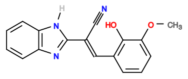

8F06

v>1.5 s=2.2

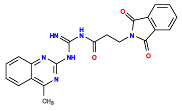

125F11

v>1.5 s=2.2

512299935 GCTGTC ACTGGA TGCATGACCATATTTGGTGTGCTGAGCTGC TCCGGT CTGATGA GTCTT TGGC AGGAC GAAACAGC

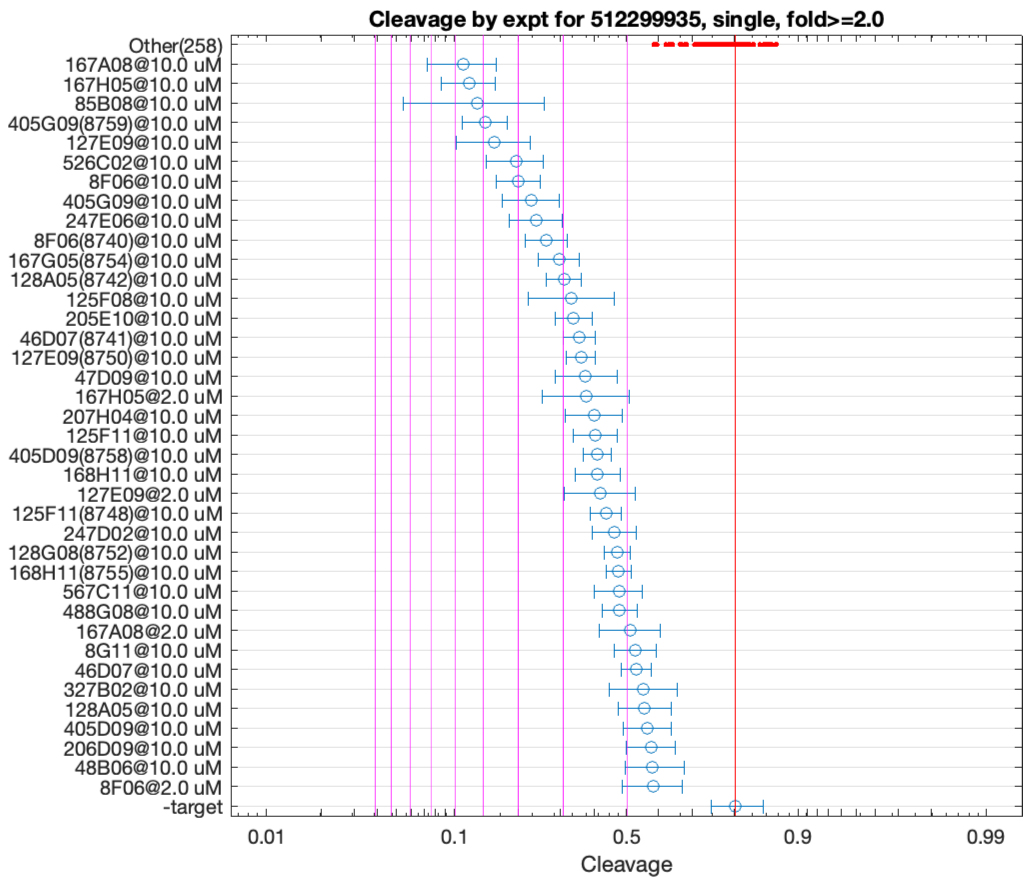

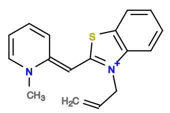

167A08

v>3.1 s=5.8

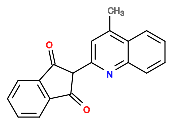

167H05

v>2.8 s=5.5

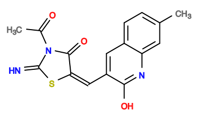

85B08

v>3.7 s=5.2

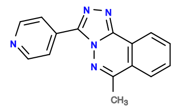

405G09

v>3.5 s=5.0

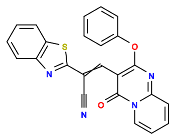

127E09

v>2.8 s=4.6

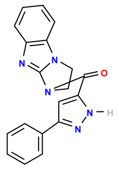

526C02

v>3.7 s=4.1

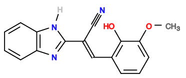

8F06

v>2.5 s=4.0

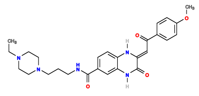

247E06

v>2.8 s=3.5

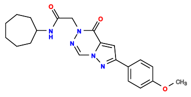

447E10

v>3.3

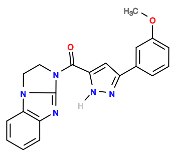

525D11

v>3.3

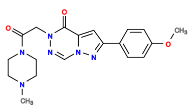

447E09

v>3.3

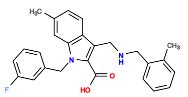

527H03

v>3.3

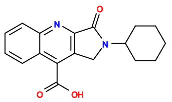

208A06

v>3.3

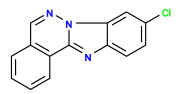

167G05

v>2.9 s=3.1

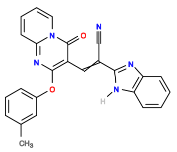

128A05

v>2.4 s=3.0

512299937 GCTGTC ACTGG ATGCATGACCATATTTTGGTGTGCCGAGCGGCA CCAGT CTGATGA GTTC GTGGCG GGAC GAAACAGC

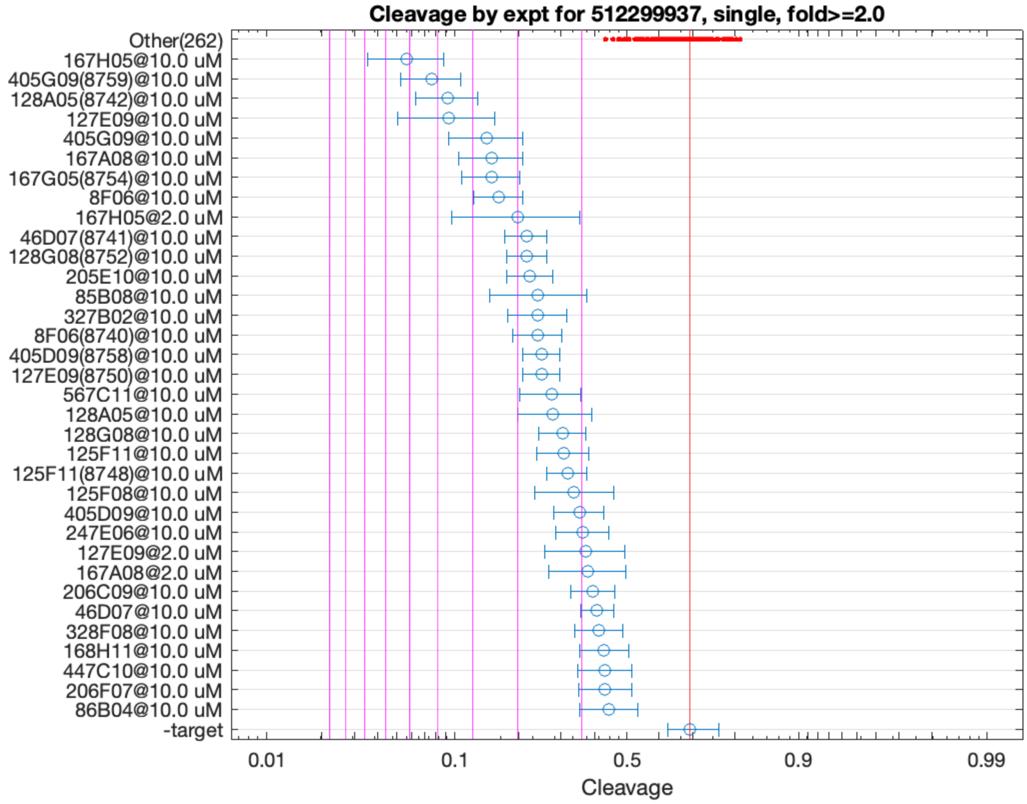

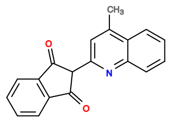

167H05

v>3.1 s=6.0

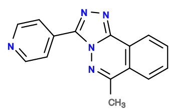

405G09

v>3.1 s=5.2

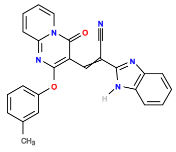

128A05

v>2.6 s=4.7

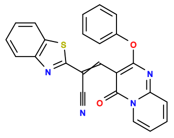

127E09

v>2.9 s=4.7

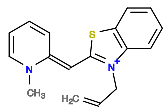

167A08

v>2.3 s=3.5

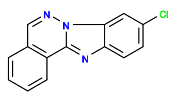

167G05

v>2.6 s=3.5

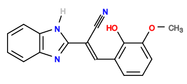

8F06

v>2.0 s=3.4

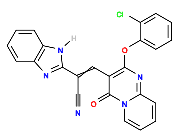

128G08

v>2.8 s=2.9

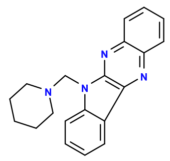

46D07

v>2.1 s=2.8

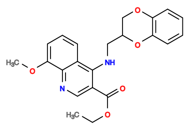

205E10

v>2.4 s=2.8

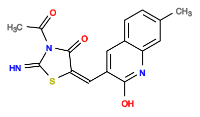

85B08

v>2.3 s=2.7

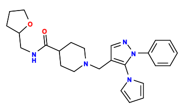

446C03

v>2.7

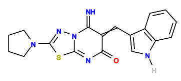

327B02

v>2.7 s=2.7

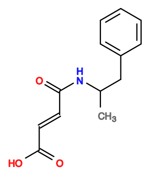

6E06

v>2.6

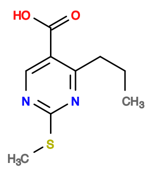

488B10

v>2.6

512329455 GCTGTC ACTGGA GTTAAGAGTGGTTGTTGCTACTGTGCCGATCTAA TCTGGT CTGACGA GTCC GTGGCG GGAC GAAACAGC

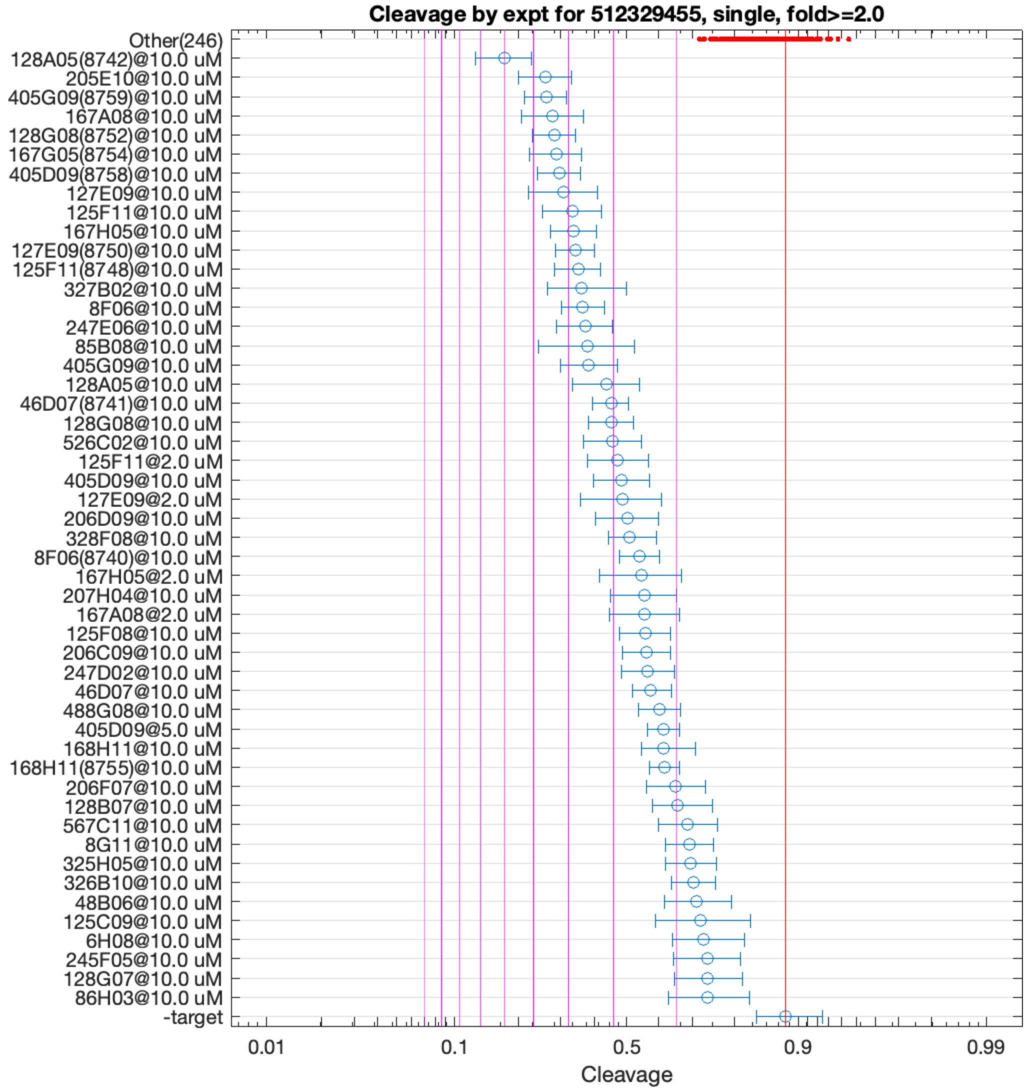

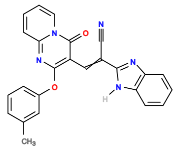

128A05

v>2.9 s=6.0

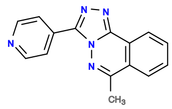

405G09

v>3.1 s=4.7

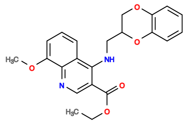

205E10

v>3.5 s=4.5

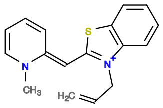

167A08

v>2.8 s=4.4

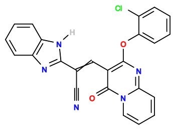

128G08

v>3.4 s=4.4

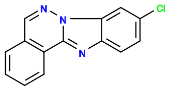

167G05

v>3.0 s=4.2

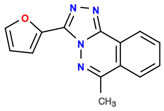

405D09

v>2.7 s=4.2

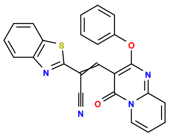

127E09

v>2.9 s=4.1

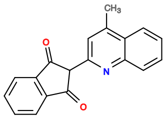

167H05

v>2.9 s=3.9

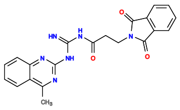

125F11

v>2.5 s=3.9

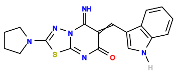

327B02

v>3.2 s=3.7

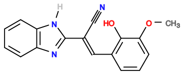

8F06

v>2.5 s=3.7

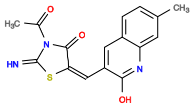

85B08

v>2.8 s=3.6

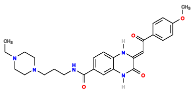

247E06

v>2.5 s=3.6

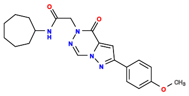

447E10

v>3.5

513125001 GCTGTC ACTGGA CATTTCTGGGCATTTGGGTTGAATTGGCGAA TCCGGT CTGACGA GTCCTGTA GTCGGTTACCGTGAAGCTCGGG TGTGGGAC GAAACAGC

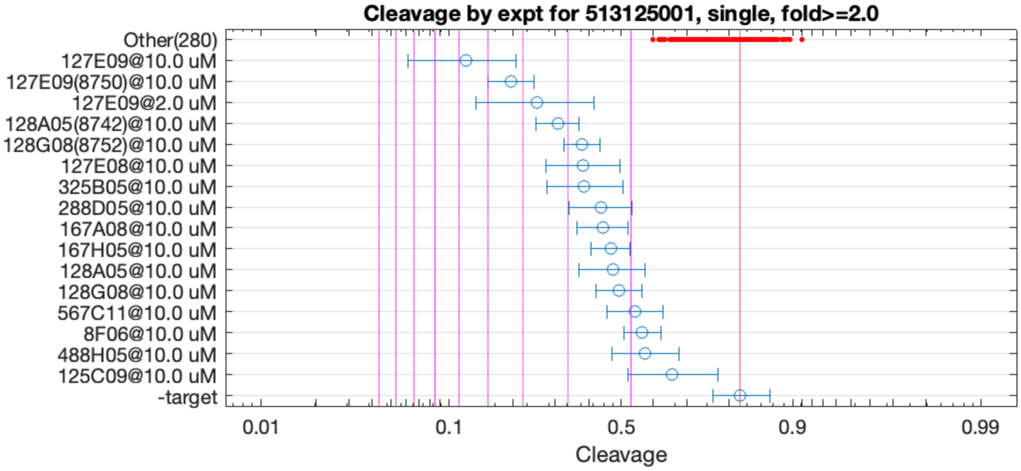

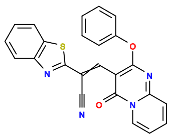

127E09

v>3.3 s=5.8

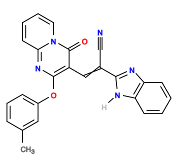

128A05

v>1.7 s=3.2

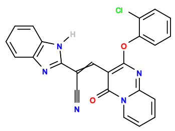

128G08

v>1.9 s=2.8

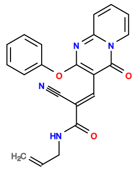

127E08

v>1.7 s=2.7

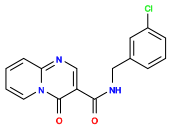

325B05

v>1.4 s=2.7

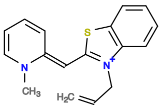

167A08

v>1.4 s=2.5

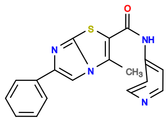

288D05

v>1.8 s=2.4

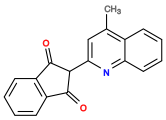

167H05

v>1.3 s=2.2

513135887 GCTGTC ACTGGA AACCTGCGGTTGGCAAAAGAGAGACACCTC TCCGGT CTGACGA GTCC GATGTGTCTGCGAATGTCTGCGTGTTGGTG GGAC GAAACAGC

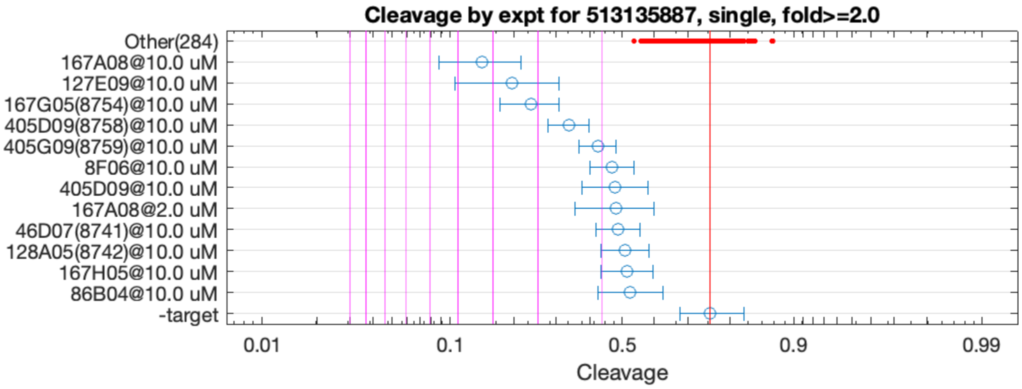

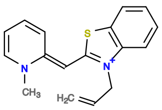

167A08

v>2.1 s=4.2

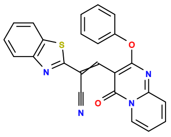

127E09

v>1.8 s=3.5

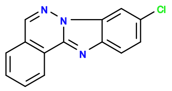

167G05

v>1.8 s=3.2

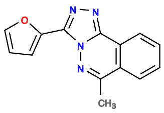

405D09

v>1.8 s=2.5

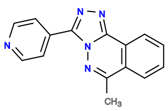

405G09

v>1.7 s=2.0

513140430 GCTGTC ACTGGAG CCTAGCGGCACTACAACCAACGAACACCT CTCCGGT CTGACGA GTCC GATGTGTCTGCGTGTGGTG GGAC GAAACAGC

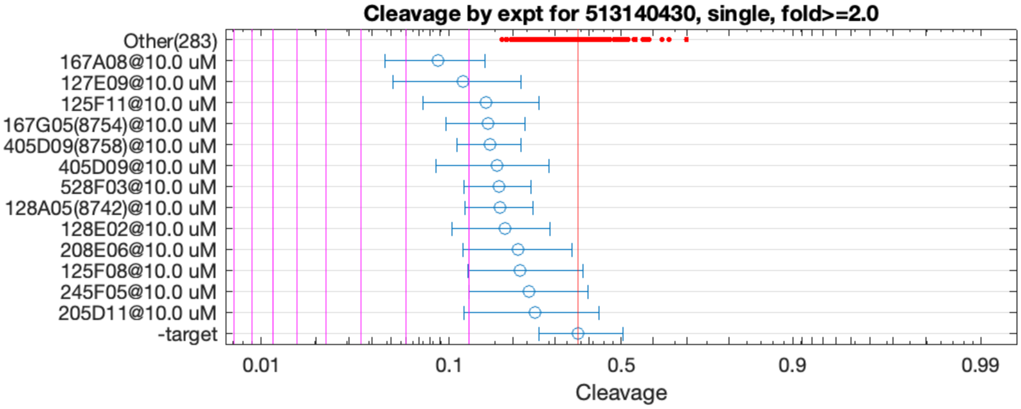

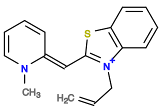

167A08

v>1.4 s=2.4

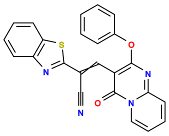

127E09

v>1.2 s=2.0

513339614 GCTGTC ACTGGA TTTGGGAAACAACGAAACGCCTCAATACAT TCCGGT CTGACGA GTC GTTT GAC GAAACAGC GCGGGACGAAACAGC

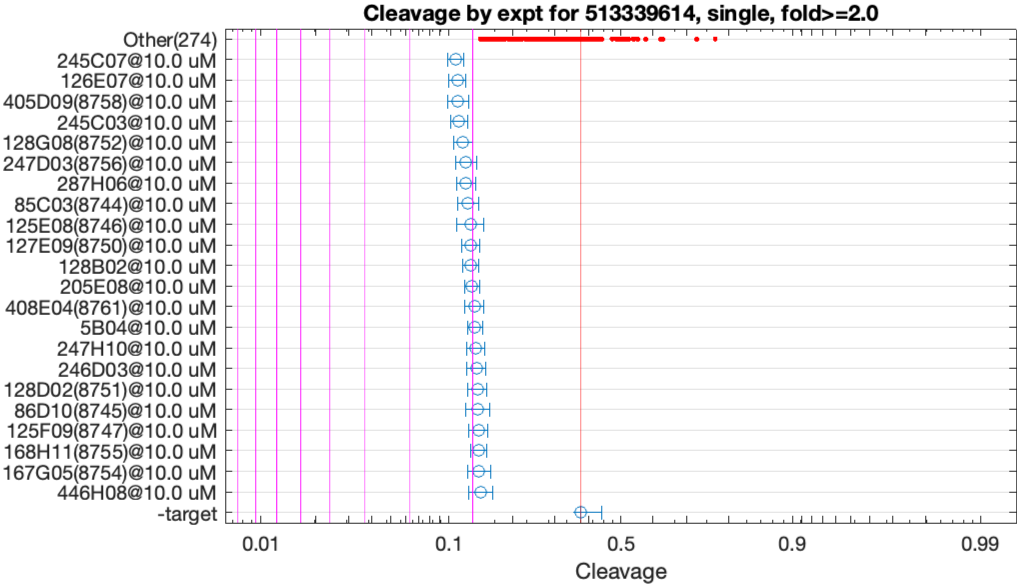

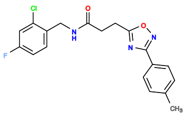

245C07

v>1.0 s=2.2

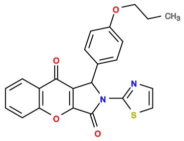

126E07

v>1.1 s=2.2

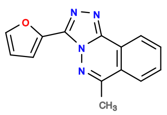

405D09

v>1.0 s=2.2

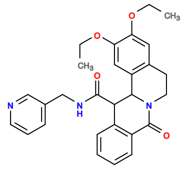

245C03

v>1.1 s=2.2

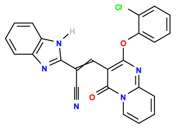

128G08

v>1.1 s=2.1

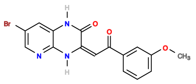

247D03

v>1.0 s=2.1

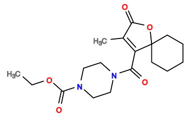

287H06

v>1.0 s=2.1

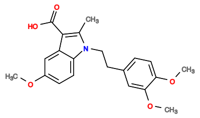

85C03

v>0.9 s=2.0

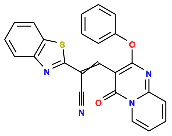

127E09

v>1.0 s=2.0

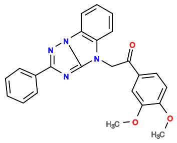

125E08

v>1.1 s=2.0

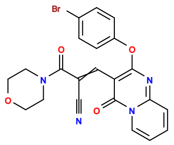

128B02

v>1.0 s=2.0

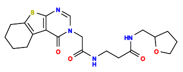

205E08

v>1.0 s=2.0

513400071 GCTGTC ACTGGA TGGAGGCT TTCAGT CTGACGA GTCCTG CAACAACCCAATTGCACGCAAGCC CGGGAC GAAACAGC

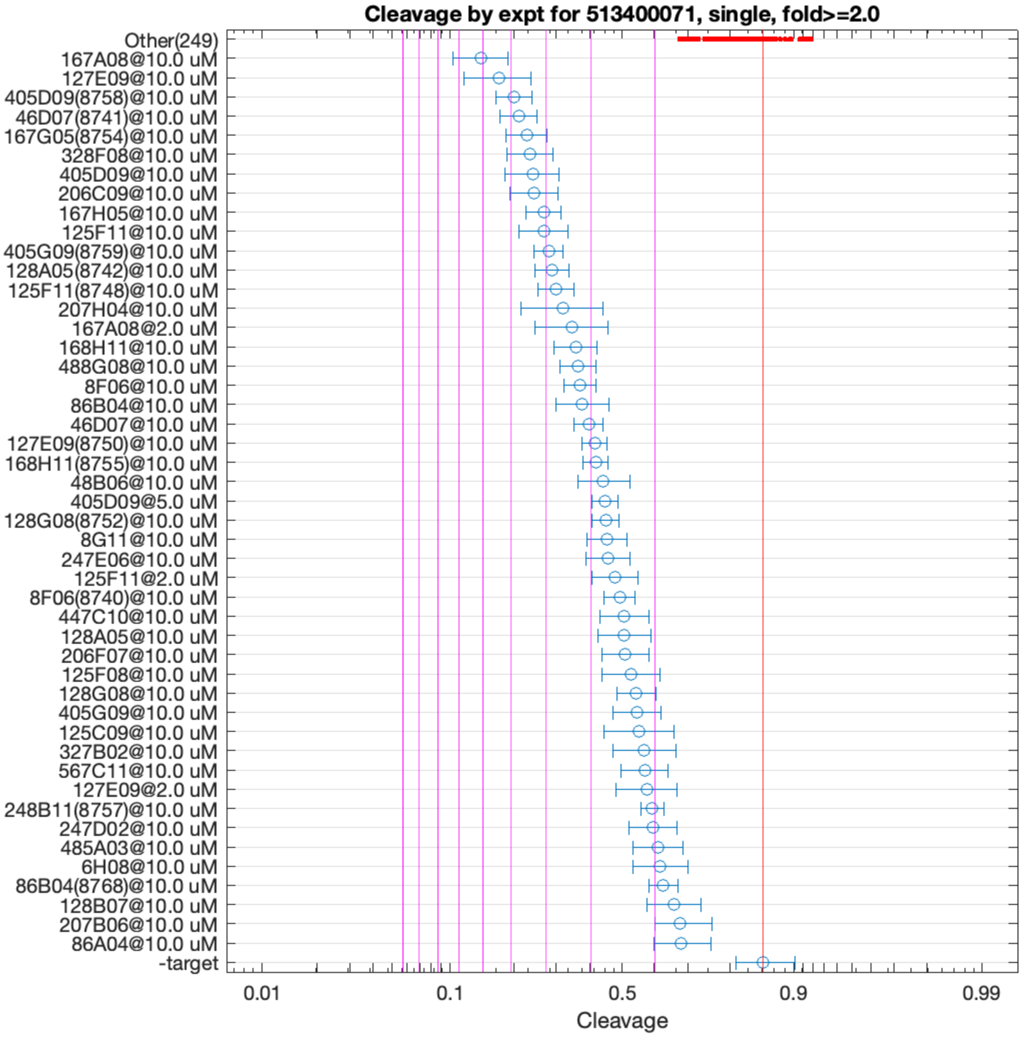

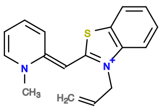

167A08

v>3.9 s=6.2

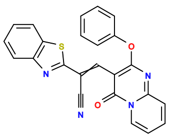

127E09

v>3.3 s=5.6

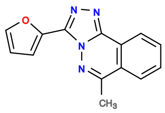

405D09

v>3.6 s=4.9

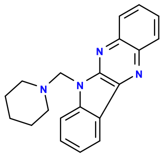

46D07

v>3.4 s=4.8

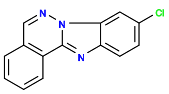

167G05

v>3.5 s=4.5

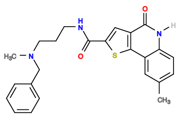

328F08

v>3.5 s=4.5

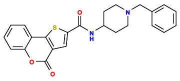

206C09

v>3.6 s=4.4

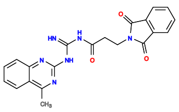

125F11

v>2.8 s=4.1

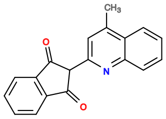

167H05

v>3.1 s=4.1

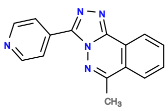

405G09

v>3.4 s=3.9

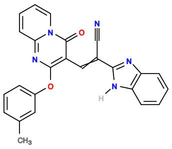

128A05

v>3.0 s=3.9

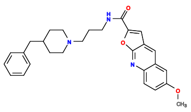

207H04

v>3.2 s=3.7

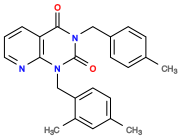

328B03

v>3.6

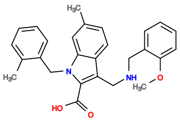

526C11

v>3.6

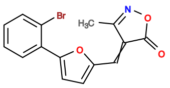

126B06

v>3.6

514037045 GCTGTC ACTGGA AGCATGACCATATTTGGTGTGCCGAGCGGC TCCGGT CTGATGA GTCTT TGGC AGGAC GAAACAGC

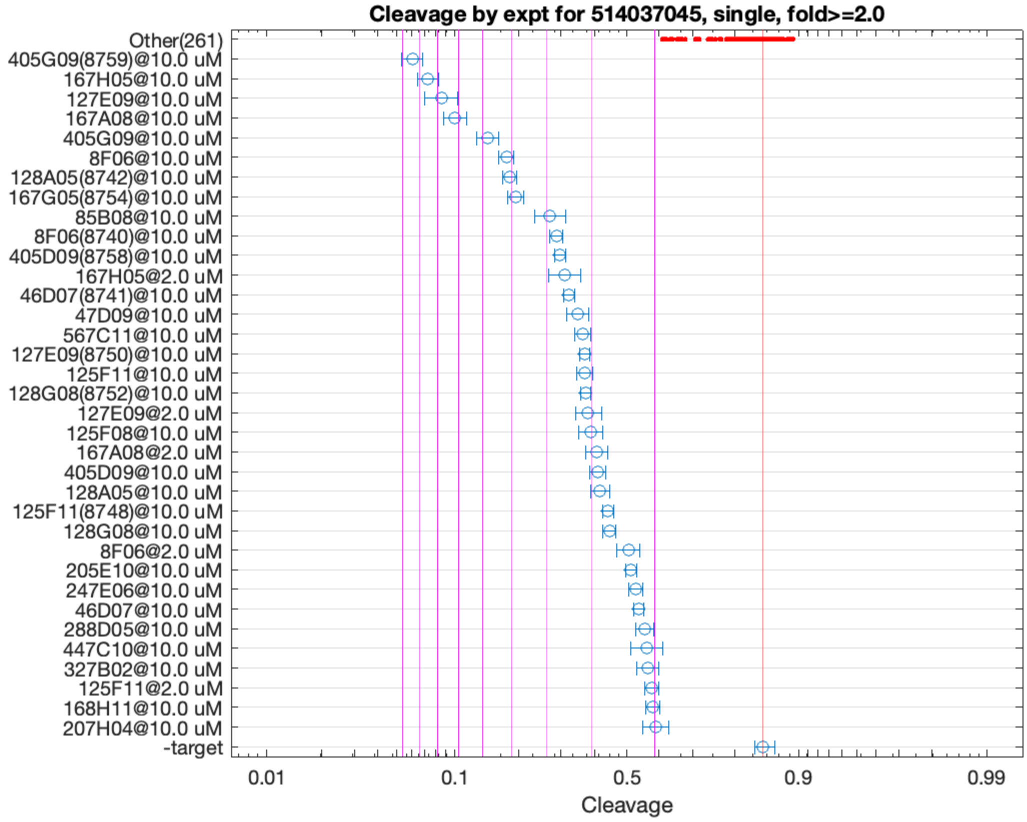

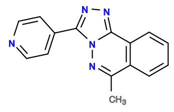

405G09

v>5.8 s=9.4

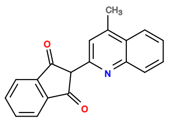

167H05

v>3.2 s=8.5

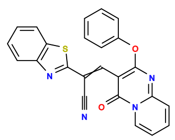

127E09

v>3.8 s=7.8

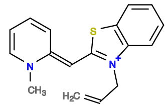

167A08

v>3.5 s=7.2

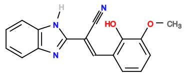

8F06

v>2.9 s=5.2

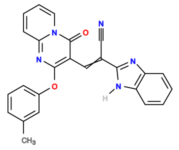

128A05

v>3.4 s=5.1

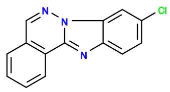

167G05

v>3.6 s=4.8

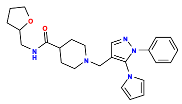

446C03

v>3.9

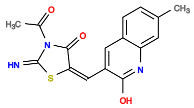

85B08

v>3.7 s=3.9

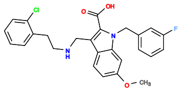

526H09

v>3.8

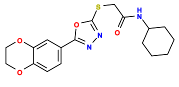

246A07

v>3.8

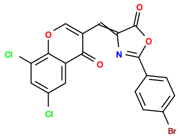

564H11

v>3.8

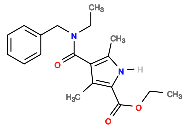

325F11

v>3.7

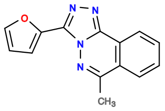

405D09

v>3.2 s=3.7

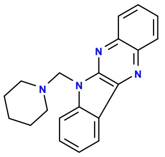

46D07

v>3.5 s=3.5

514294722 GCTGTC ACTGGA AAGTTGCGTGAGTAAGGTGCGGTAAGATCA TCCGGT CTGATGA GTCC GTTGACCCGTGCTTGAGTAGCGTCGACTGGG GGAC GAAACAGC

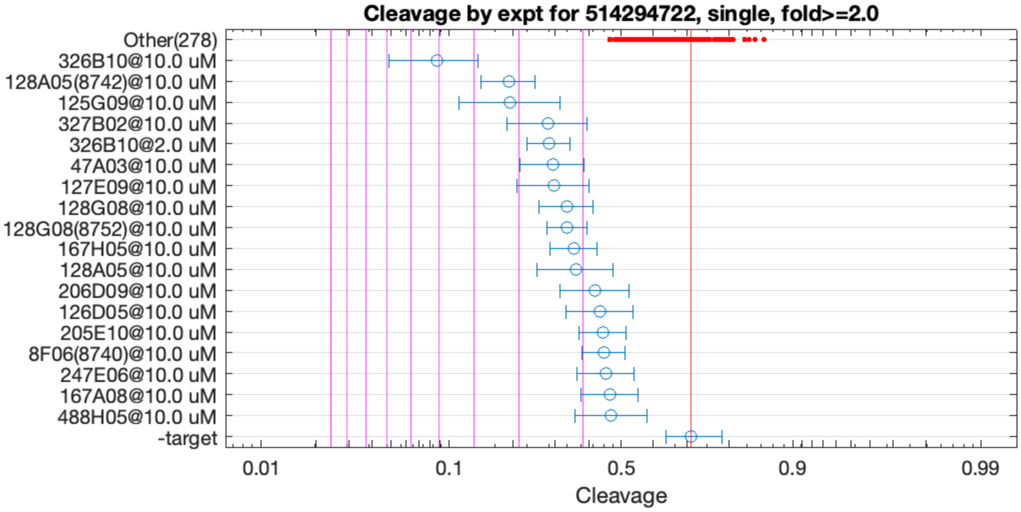

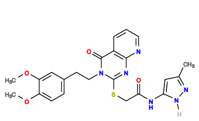

326B10

v>2.2 s=5.2

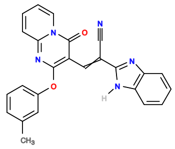

128A05

v>1.6 s=3.2

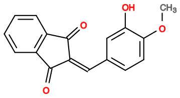

125G09

v>1.5 s=3.2

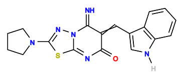

327B02

v>1.8 s=2.5

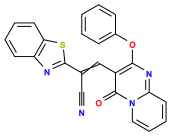

127E09

v>1.3 s=2.5

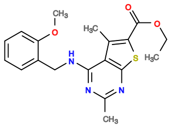

47A03

v>1.4 s=2.4

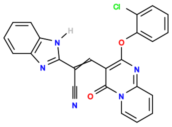

128G08

v>1.9 s=2.2

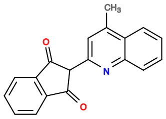

167H05

v>1.6 s=2.1

514868644 GCTGTC ACTGGA TGCATGATCATATTTGGTGTGCTGAGCTGC TCTAGT CTGACGA GTCC GTGGCG GGAC GAAACAGC

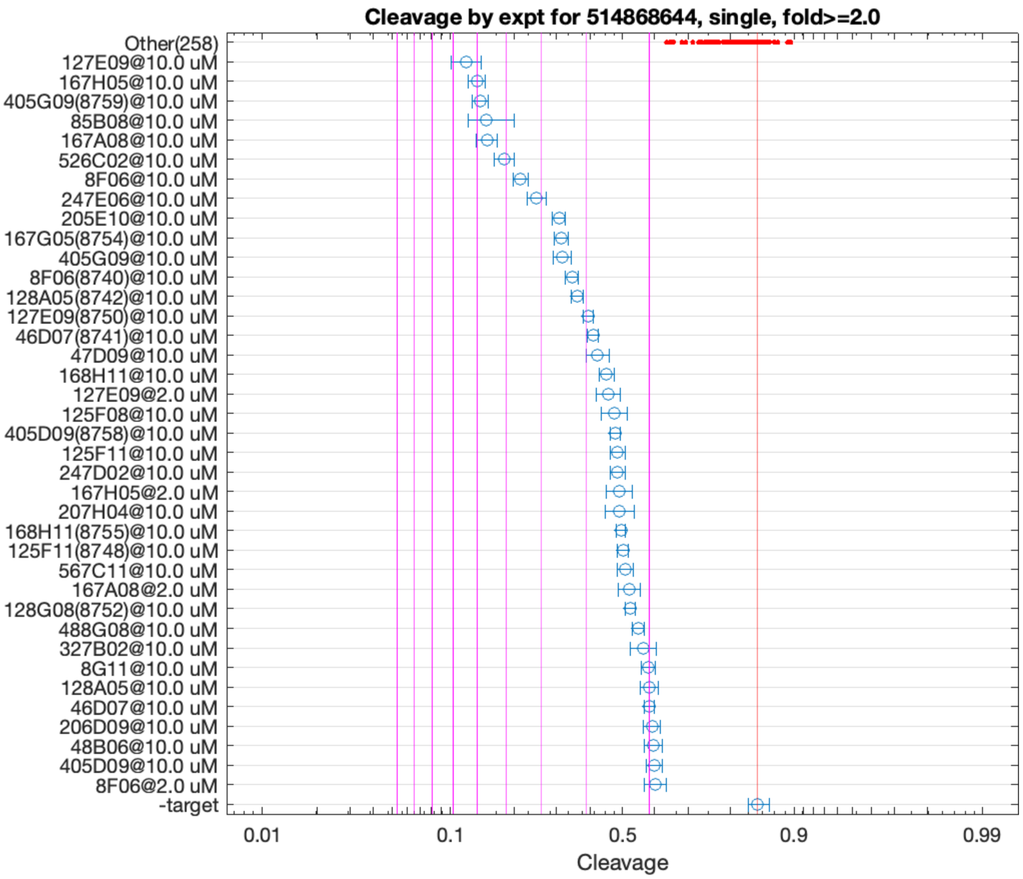

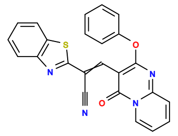

127E09

v>3.4 s=6.5

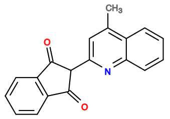

167H05

v>3.5 s=6.0

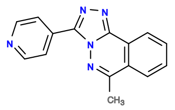

405G09

v>4.3 s=5.9

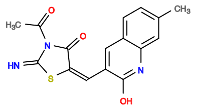

85B08

v>4.6 s=5.7

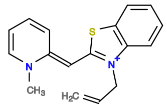

167A08

v>3.4 s=5.6

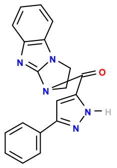

526C02

v>4.8 s=5.0

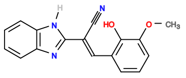

8F06

v>2.8 s=4.6

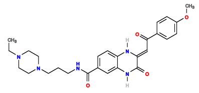

247E06

v>3.3 s=4.1

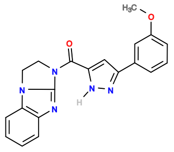

525D11

v>4.0

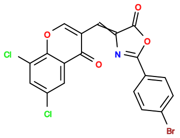

564H11

v>4.0

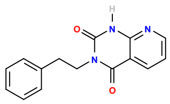

328H04

v>3.9

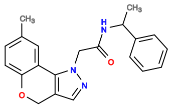

207F08

v>3.9

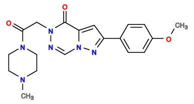

447E09

v>3.9

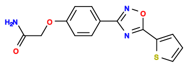

125B06

v>3.8

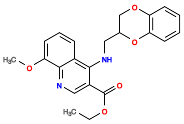

205E10

v>3.5 s=3.6

524192145 GCTGTC ACTGGA TTTCCAACTTGCGTAATTTGATGACGCCTC TCCGGT CTGATGA GTCC GATGTGTCTGCGTGTTGGTG GGAC GAAACAGC

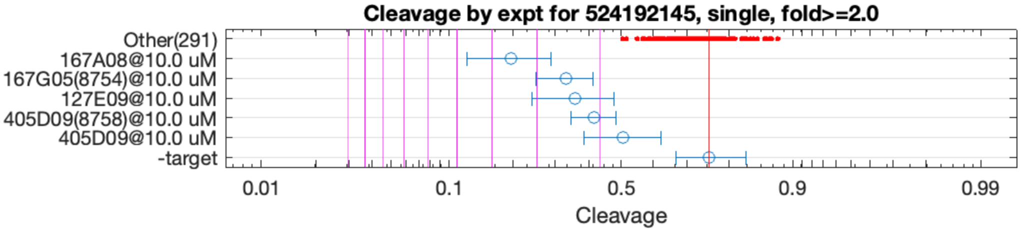

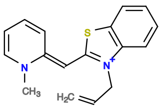

167A08

v>1.4 s=3.6

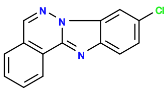

167G05

v>1.3 s=2.5

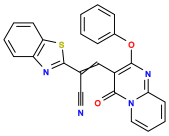

127E09

v>1.3 s=2.3

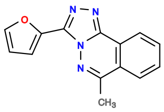

405D09

v>1.3 s=2.1

533994655 GCTGTC ACTGGA TGGAGGCT TTCAGT CTGACGA GTCCTG CAACAACCAATTGCACGCAAGCC CGGGAC GAAACAGC

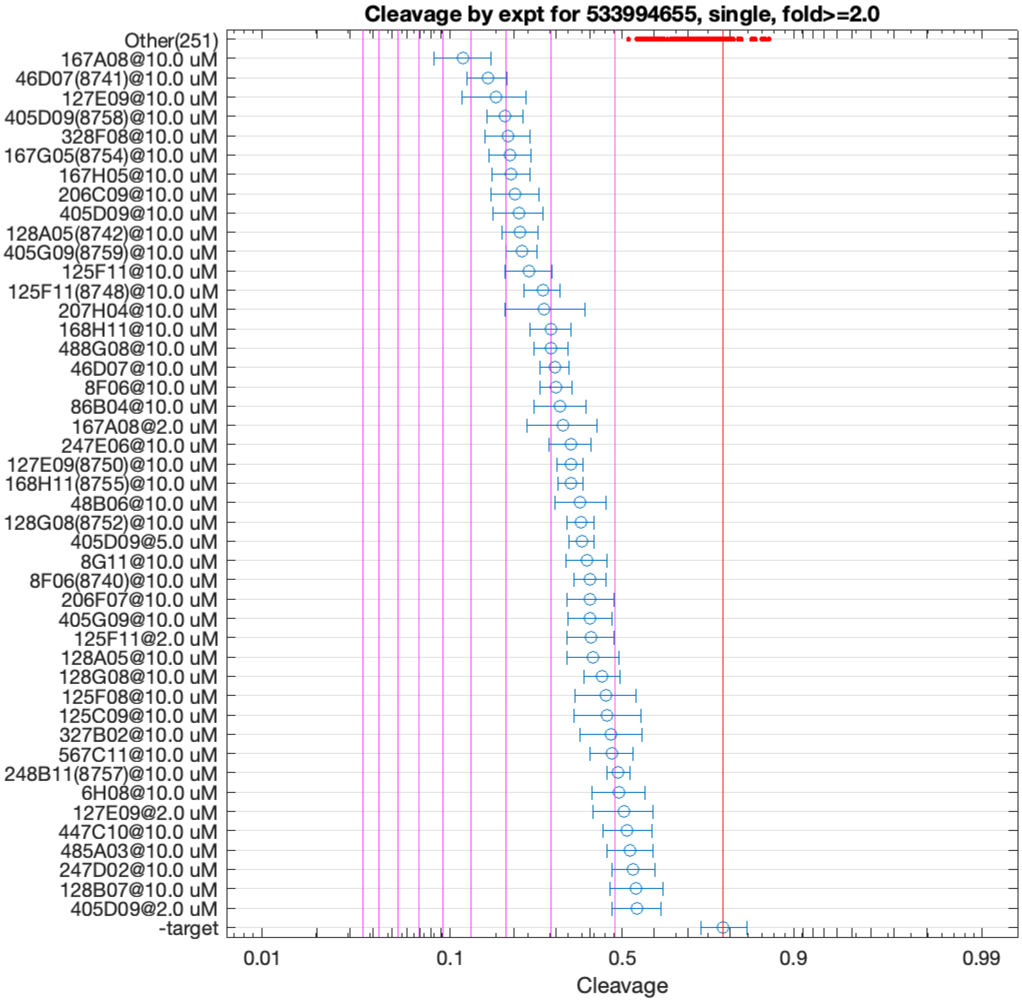

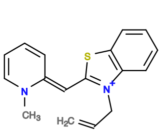

167A08

v>3.4 s=5.3

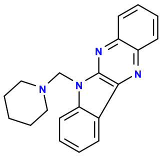

46D07

v>3.2 s=4.5

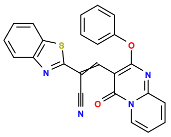

127E09

v>3.0 s=4.3

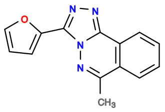

405D09

v>3.3 s=4.1

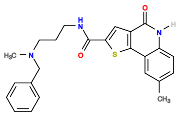

328F08

v>3.2 s=3.9

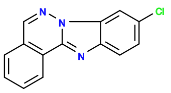

167G05

v>3.1 s=3.9

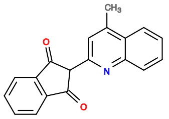

167H05

v>2.8 s=3.8

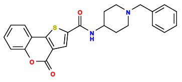

206C09

v>3.0 s=3.8

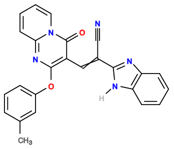

128A05

v>2.6 s=3.6

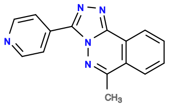

405G09

v>3.0 s=3.6

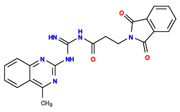

125F11

v>2.6 s=3.5

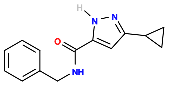

525B07

v>3.3

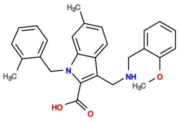

526C11

v>3.3

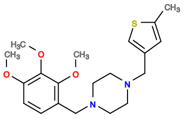

486G03

v>3.3

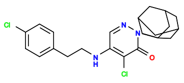

247D09

v>3.2

535993570 GCTGTC AC TGGATTGGCGTAAATCC GT CTGATGA GTCC TGCGATGCCTAATGCACGTGCCTCTCCGCC GGAC GAAACAGC

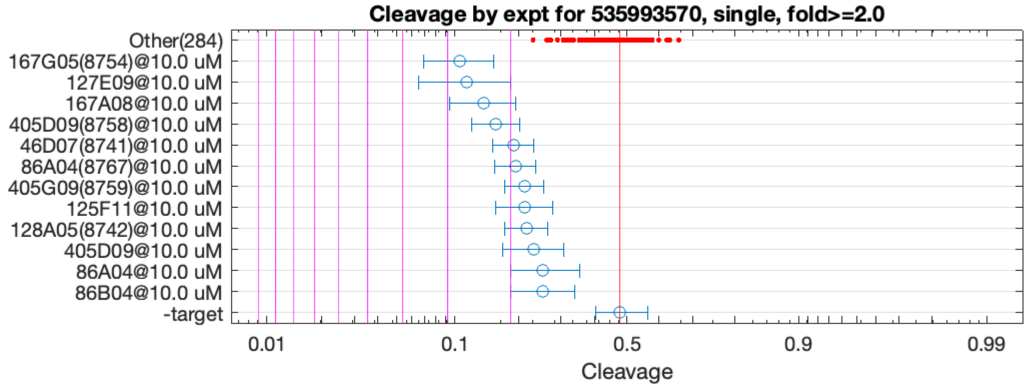

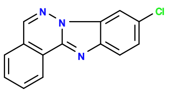

167G05

v>1.7 s=2.8

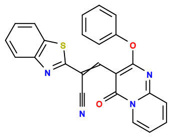

127E09

v>1.6 s=2.6

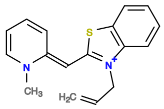

167A08

v>1.7 s=2.4

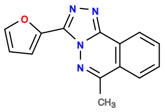

405D09

v>1.6 s=2.2

536070526 GCTGTC ACTGGA GTAACCGATCTTTCGGCGAACTTACCGATA TCCGGT CTGACGA GTCT GTGGTG GGAC GAAACAGC

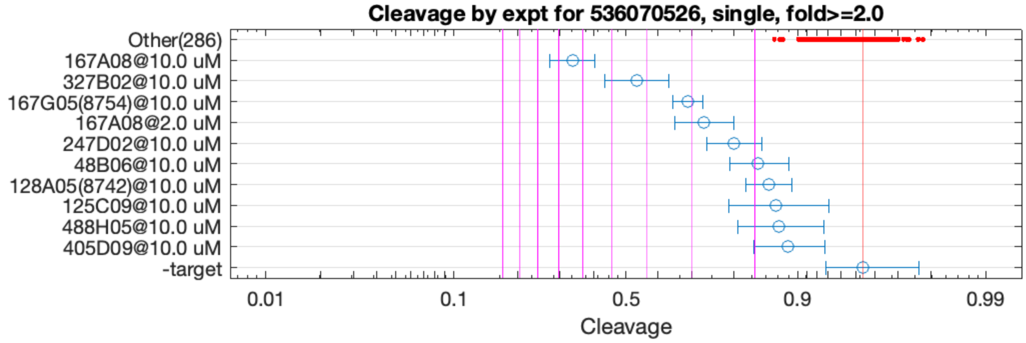

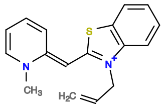

167A08

v>1.5 s=6.4

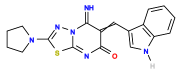

327B02

v>1.3 s=4.2

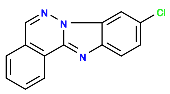

167G05

v>1.1 s=3.1

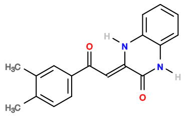

247D02

v>1.1 s=2.3

536384056 GCTGTC AC TGGAATGCGCGAACT GT CTGATGA GTCC AAATCTGCTCGATTTCGTGTGGGTGCGCG GGAC GAAACAGC

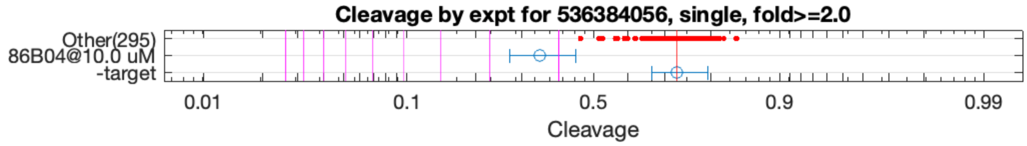

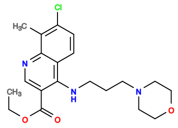

86B04

v>1.1 s=2.3

565352371 GCTGTC ACTG TATTTTCCATCACTGGATTTGAT TGGT CTGATGA GTGCGTGTATGCC GAAACAGC TCGTGATGCCCCGGGACGAAACAGC

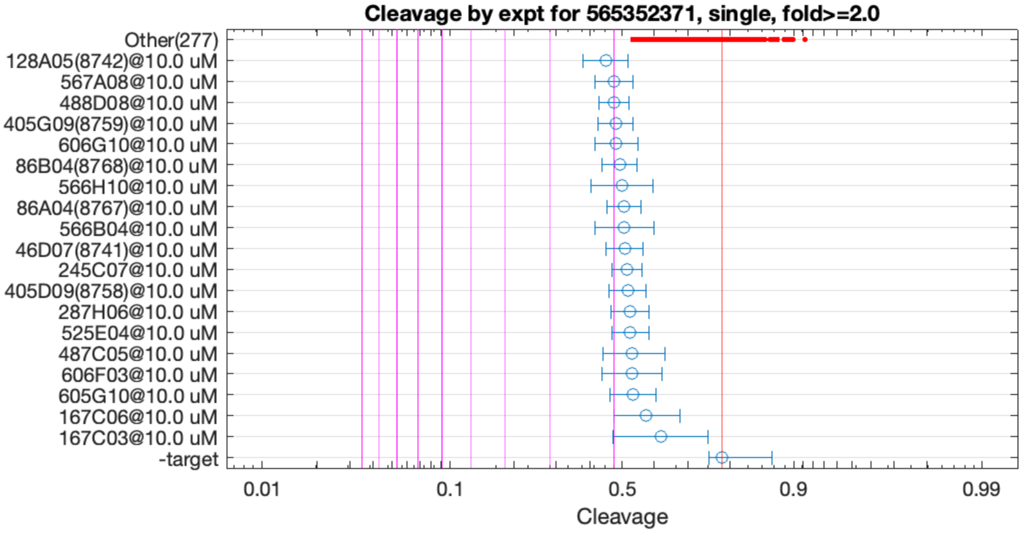

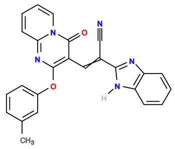

128A05

v>0.8 s=2.1

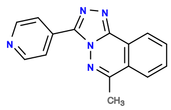

405G09

v>0.8 s=2.0

565352652 GCTGTC ACTGGA AAGGTGGTAGGAAGAGTGCTCGTCGTAACA TCCGGT CTGATGA GTCC GATAAAACGTCATGTAGAAGCAGTACCACG GGAC GAAACAGC

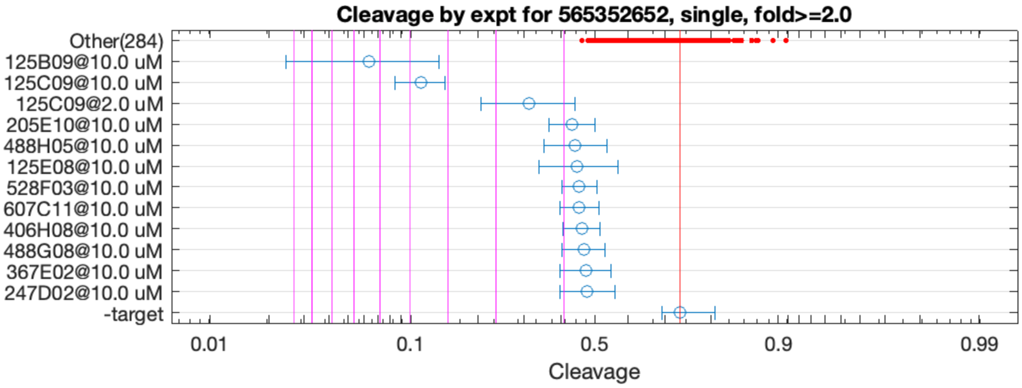

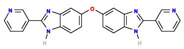

125B09

v>4.0 s=6.4

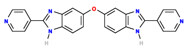

125C09

v>4.4 s=4.7

565352773 GCTGTC ACTGGAA CGGGACA TTCTGGT CTGATGA GTCT ACTGCCTCTGCACTATCTGCAAGCGTACCG GGAC GAAACAGC

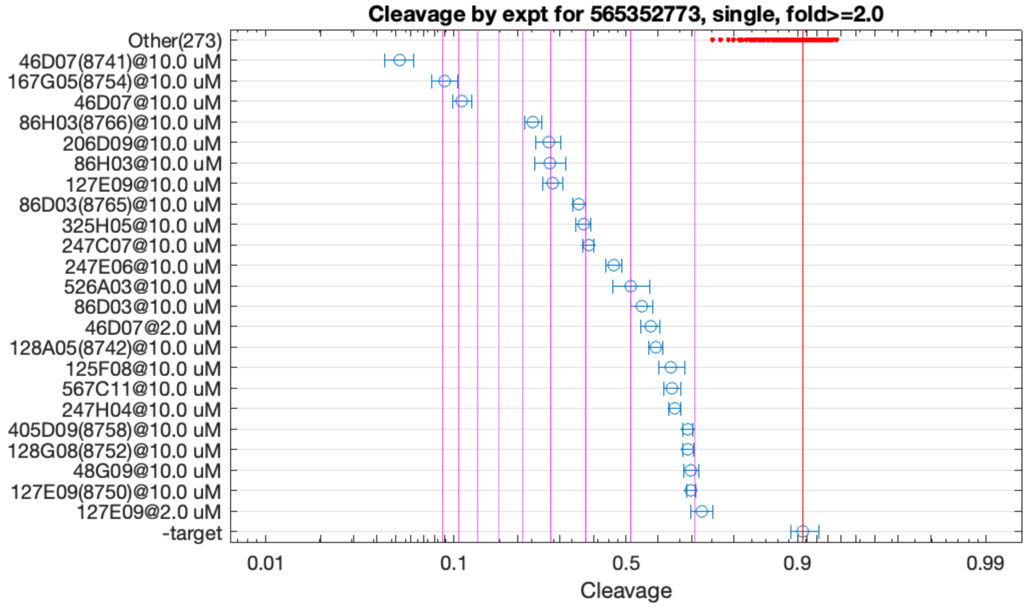

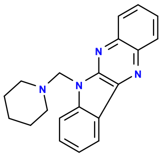

46D07

v>7.7 s=13.2

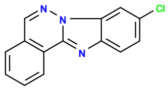

167G05

v>4.6 s=9.8

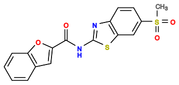

86H03

v>3.4 s=5.6

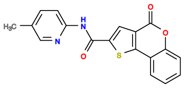

206D09

v>4.6 s=5.1

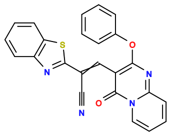

127E09

v>2.3 s=5.0

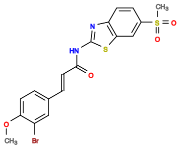

86D03

v>3.9 s=4.2

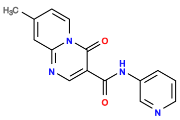

325H05

v>4.0 s=4.1

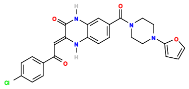

247C07

v>3.2 s=3.9

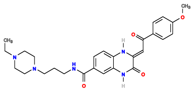

247E06

v>4.2 s=3.4

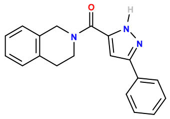

526A03

v>2.2 s=3.0

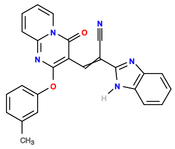

128A05

v>2.0 s=2.6

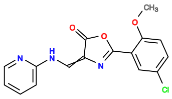

567C11

v>2.5 s=2.3

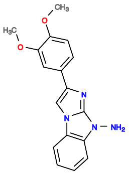

125F08

v>2.5 s=2.3

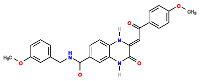

247H04

v>2.3 s=2.3

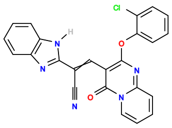

128G08

v>2.2 s=2.1

565352780 GCTGTC ACTGGAA CTAGAT TTTCGGT CTGAAGA GTCC ACGCATCTCTGTGTGTGTTGGTGGGACGAAACAGGTTGTGTTGCC GGAC GAAACAGC

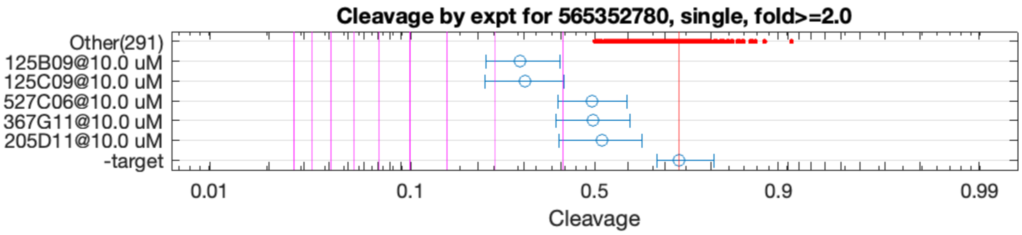

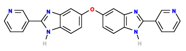

125B09

v>2.0 s=2.6

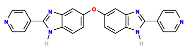

125C09

v>2.1 s=2.6

565352815 GCTGTC ACTGGA AGCGTCTACGACAGGTAGGGAGTGACACAA TCCGGT CTGATGA GCACAAC GAAACAGC ACTAACGCCGGACGAAACAGC

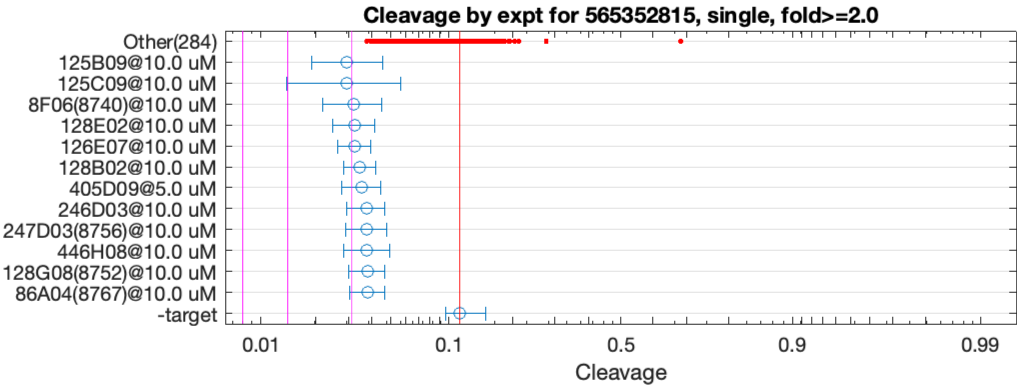

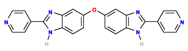

125C09

v>1.7 s=2.1

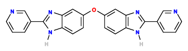

125B09

v>1.5 s=2.1

565352879 GCTGTC ACTGGA ATGCGAAAAGTGGGGAATTGAAGAGCCTCA TCCGGT CTGATGA GTG ATGCCA CAC GAAACAGC CTCTTGTGCCGGACGAAACAGC

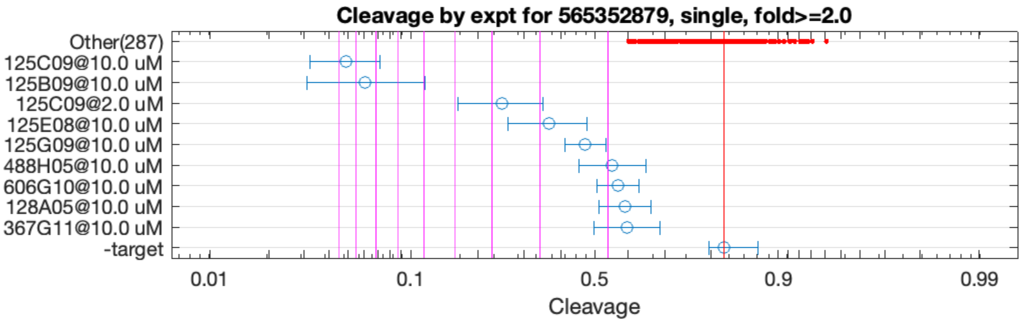

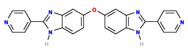

125C09

v>4.8 s=9.6

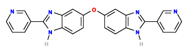

125B09

v>4.9 s=8.5

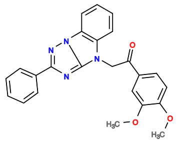

125E08

v>1.1 s=2.9

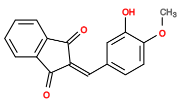

125G09

v>1.5 s=2.3

565353287 GCTGTC ACTGGA GACCTACGCGGTCAGCGCAAACCTCGCATA TCCGGT CTGACGA GTCTC ATAGACGAAACAGCGACGCCCC GGGAC GAAACAGC

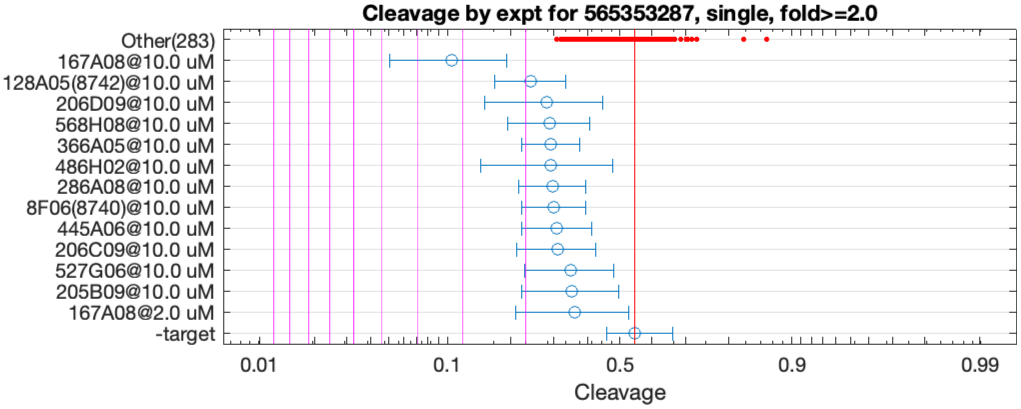

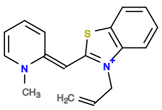

167A08

v>1.2 s=3.2

565353501 GCTGTC ACTGGA TAAGAAGAGCTACTAGGTAGGGAAGAGGCT TCCGGT CTGATGA GACCTTGAATGGC GAAACAGC ACTACGTTGCCCGGGACGAAACAGC

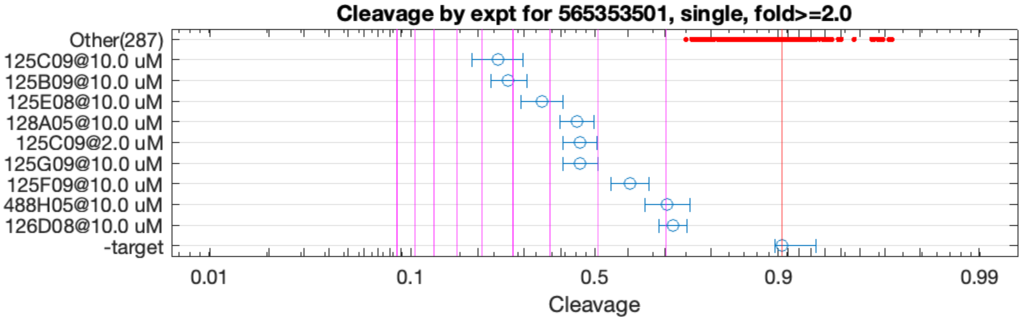

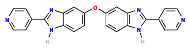

125C09

v>4.3 s=5.4

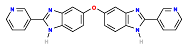

125B09

v>4.1 s=5.1

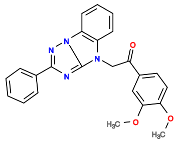

125E08

v>1.8 s=4.2

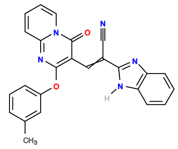

128A05

v>1.2 s=3.4

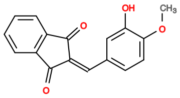

125G09

v>2.5 s=3.3

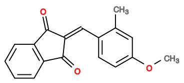

125F09

v>1.9 s=2.5

565354562 GCTGTC ACTG GATTTTCAGTTCGACGAACACTGGATACCGTAAA CAGT CTGAAGA GTCC AAATCTGCTCGATCGCGTGTGGGTGCGGG GGAC GAAACAGC

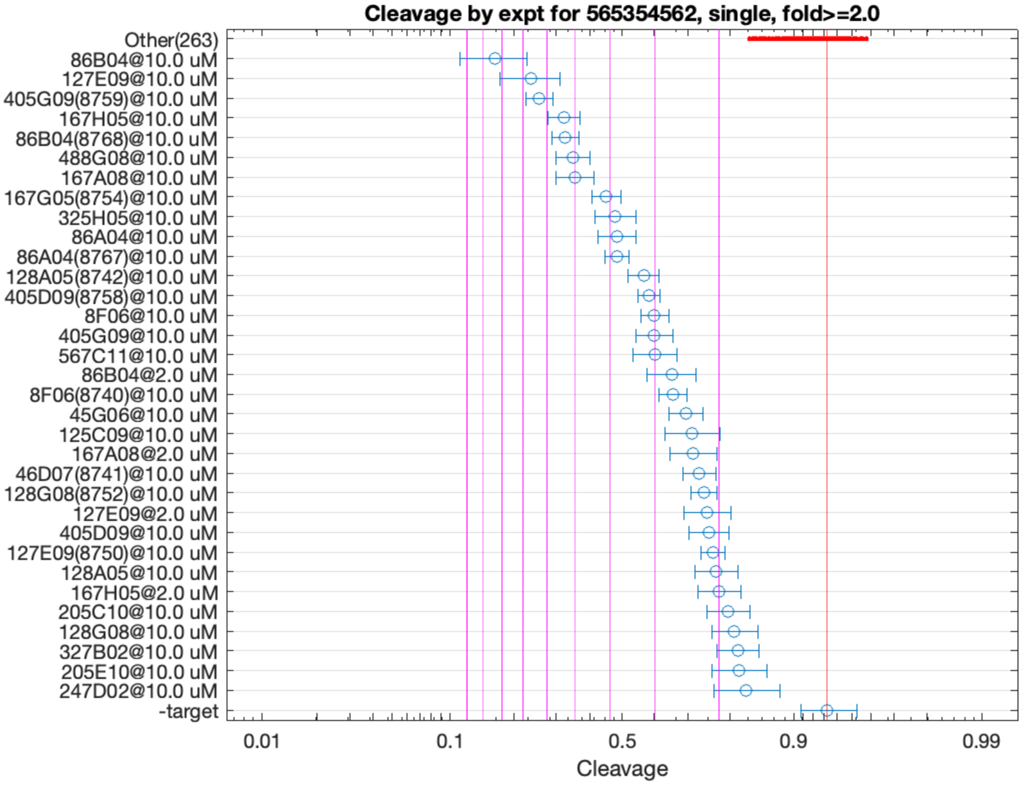

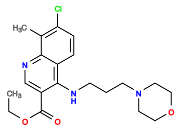

86B04

v>3.5 s=8.4

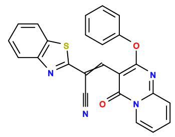

127E09

v>2.6 s=6.5

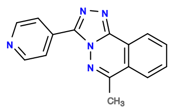

405G09

v>3.4 s=6.3

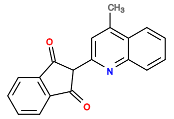

167H05

v>2.6 s=5.4

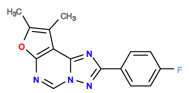

488G08

v>2.7 s=5.1

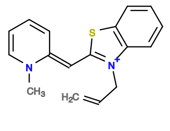

167A08

v>2.3 s=5.0

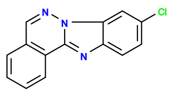

167G05

v>2.8 s=4.1

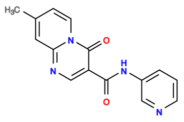

325H05

v>3.6 s=3.9

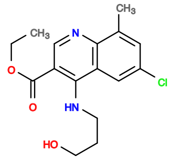

86A04

v>2.8 s=3.8

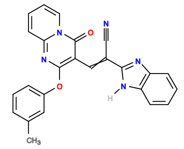

128A05

v>2.1 s=3.2

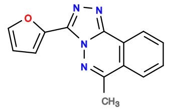

405D09

v>2.4 s=3.1

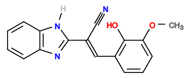

8F06

v>2.1 s=3.0

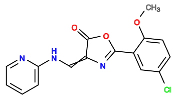

567C11

v>2.5 s=2.9

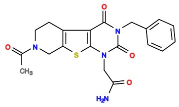

327A11

v>2.9

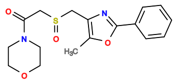

285E02

v>2.8

565354742 GCTGTC ACTGG ATTTTCCAGGATCACTGGATGCATGACCATATCTTGGTGTGCTGAGCGGCA CCAGT CTGATGA GTTC GTGGCG GGAC GAAACAGC

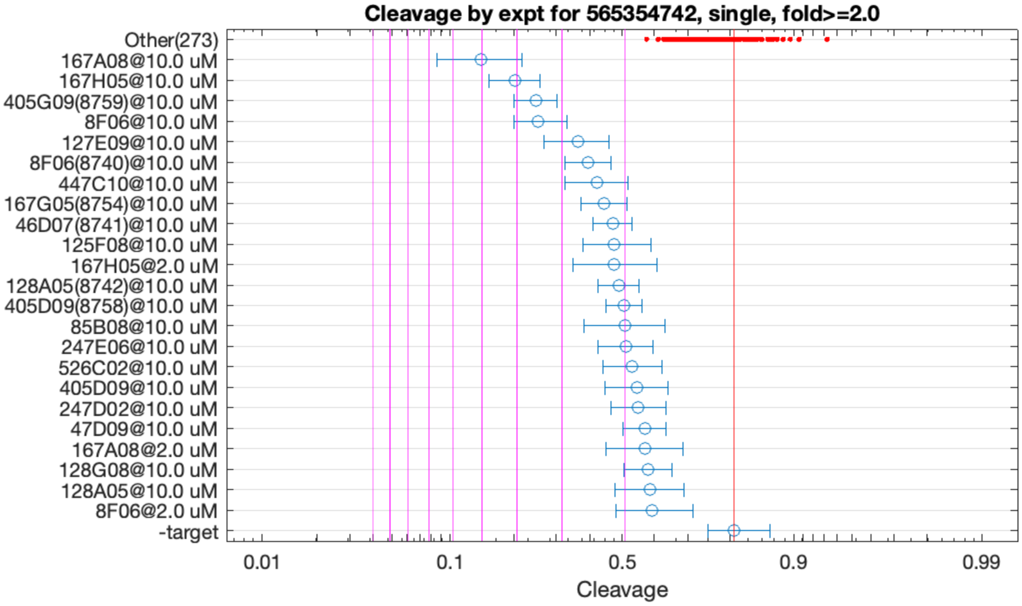

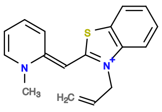

167A08

v>2.5 s=5.0

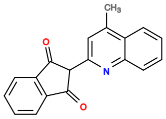

167H05

v>2.5 s=4.0

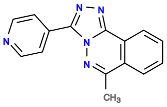

405G09

v>2.4 s=3.5

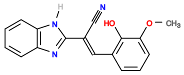

8F06

v>1.9 s=3.5

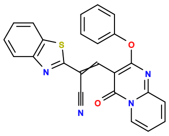

127E09

v>1.9 s=2.7

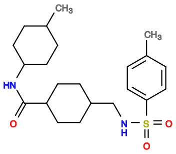

605E10

v>2.4

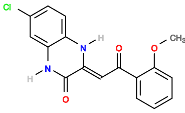

247C02

v>2.3

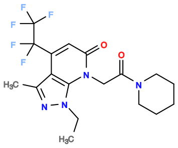

488F10

v>2.3

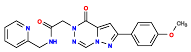

447C10

v>2.2 s=2.3

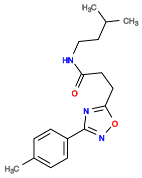

245A06

v>2.3

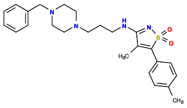

446E08

v>2.3

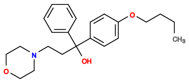

125E11

v>2.3

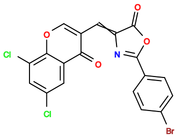

564H11

v>2.3

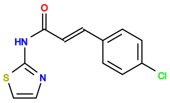

6D10

v>2.3

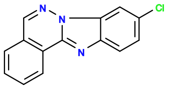

167G05

v>2.2 s=2.3

565355200 GCTGTC ACTGG CCTTCCTTTTTCACTGGAACTGGATGCGAT CTAGT CTGACGA CTCCTGCGATGGAC GAAACAGC TTGGCCCGGGACGAAACAGC

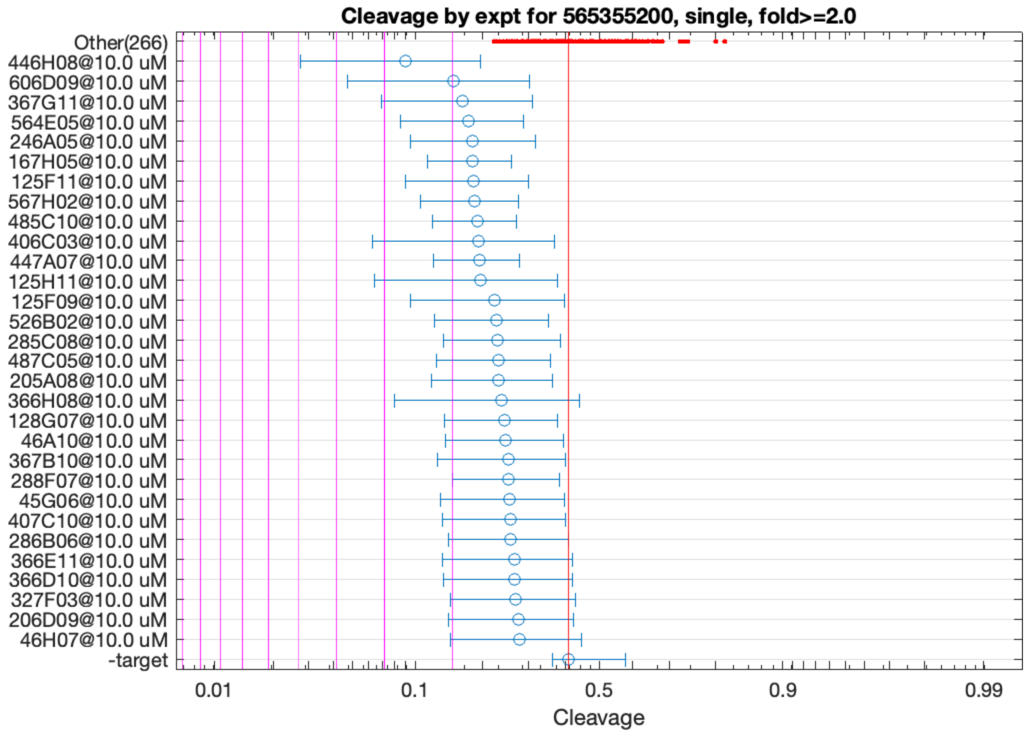

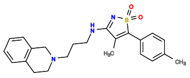

446H08

v>0.7 s=2.6

565358693 GCTGTC ACTGGA AACTCTCTGAATGAAGCGGGTATCGCAATG TCCGGT CTGATGA GTCC GATGCGGTACAGGTCAGGGTTGAATGCG GGAC GAAACAGC

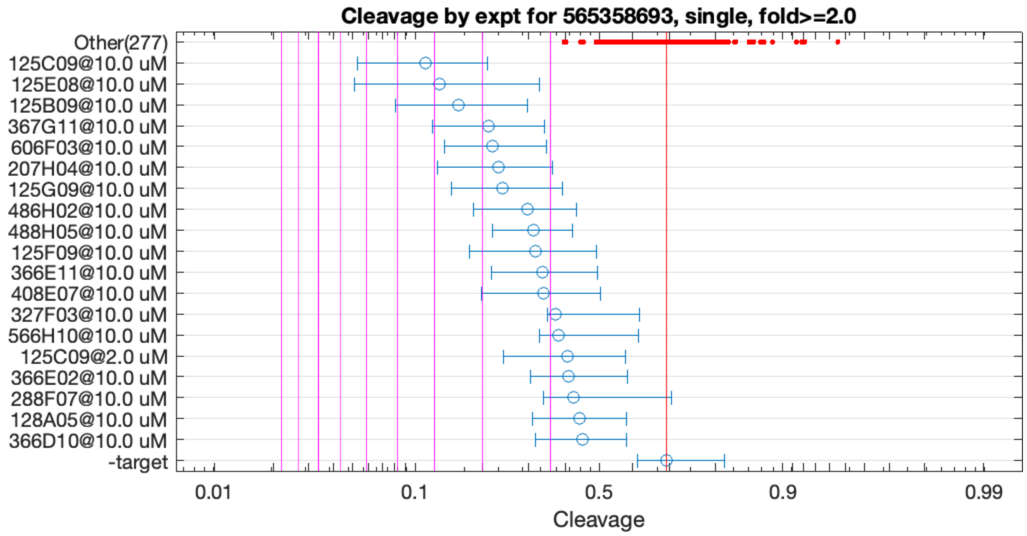

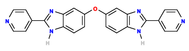

125C09

v>2.6 s=4.1

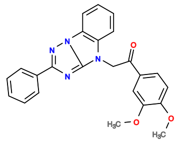

125E08

v>1.1 s=4.1

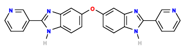

125B09

v>2.5 s=3.5

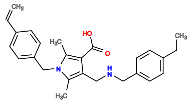

367G11

v>0.9 s=2.9

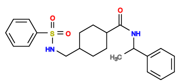

606F03

v>0.6 s=2.8

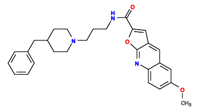

207H04

v>0.8 s=2.7

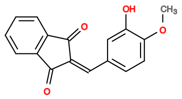

125G09

v>1.2 s=2.7

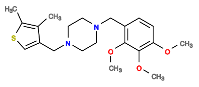

486H02

v>0.8 s=2.3

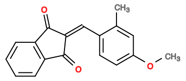

125F09

v>1.1 s=2.2

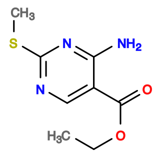

488H05

v>0.8 s=2.2

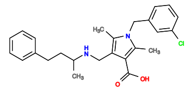

366E11

v>0.7 s=2.1

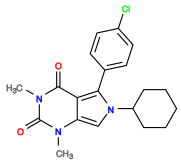

408E07

v>0.8 s=2.1

565359918 GCTGTC ACTGGA ACTGGATGCATGACCATATTTGGTGTGCCGAGCTGCC TCTAGT CTGACGA GTCC GTGGCG GGAC GAAACAGC

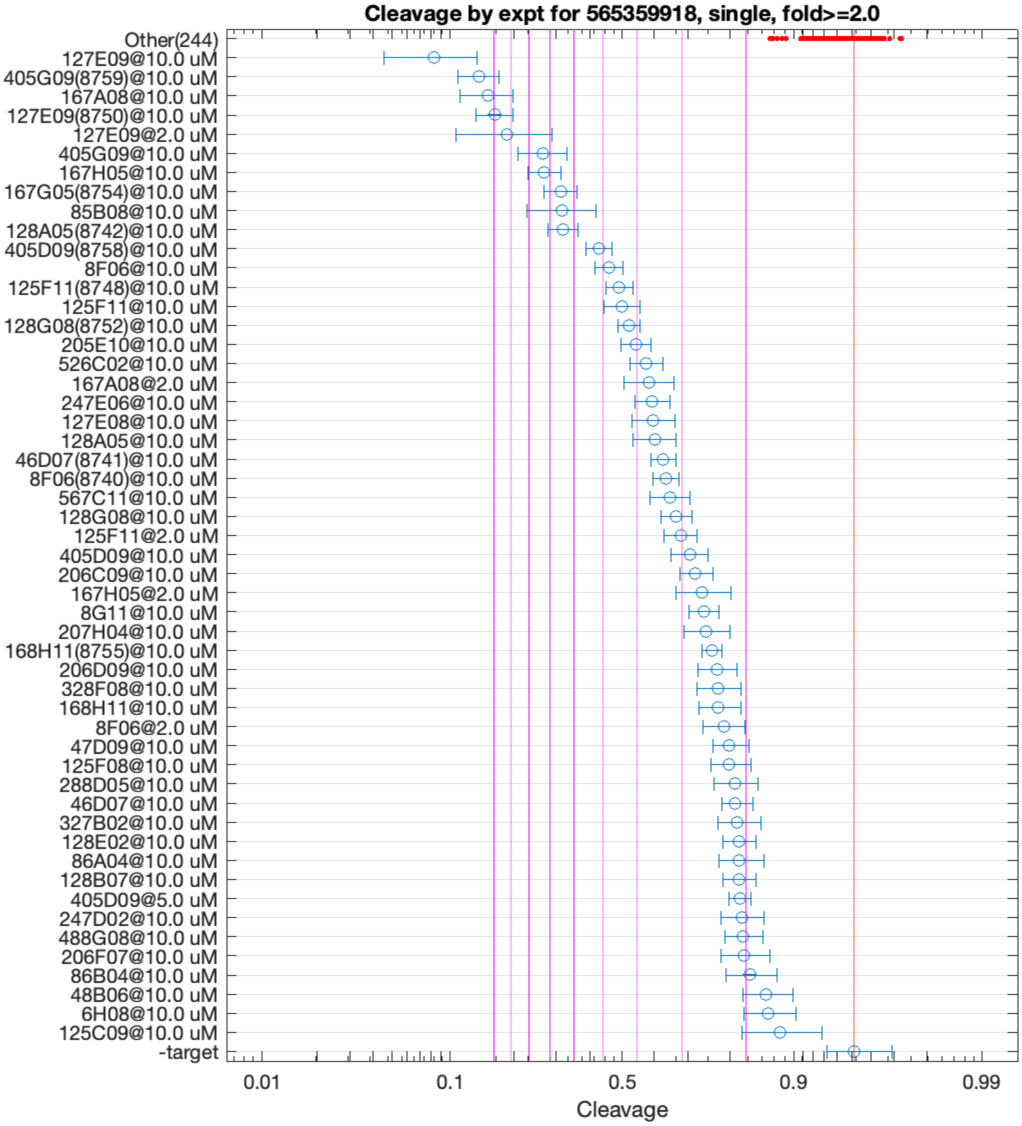

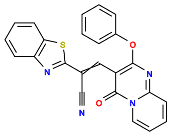

127E09

v>6.0 s=14.4

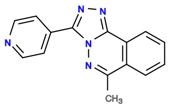

405G09

v>6.6 s=10.9

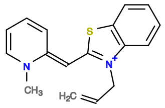

167A08

v>4.5 s=10.6

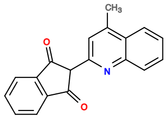

167H05

v>4.1 s=7.2

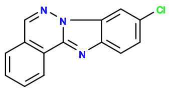

167G05

v>4.0 s=6.5

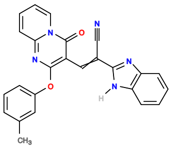

128A05

v>3.8 s=6.5

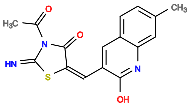

85B08

v>4.1 s=6.4

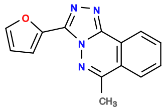

405D09

v>3.6 s=5.1

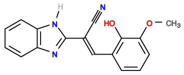

8F06

v>3.2 s=4.9

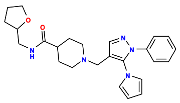

446C03

v>4.5

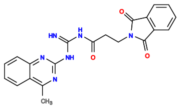

125F11

v>3.0 s=4.4

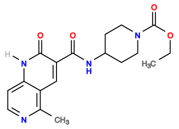

608B07

v>4.4

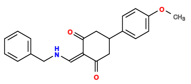

48D04

v>4.3

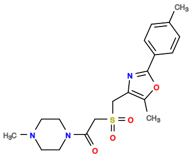

285A10

v>4.3

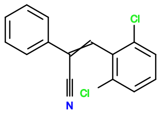

8G09

v>4.3

565360115 GCTGTC ACTGGAA GAGCGCTTAACGTAAGTCACTGGATGA TTCCGGT CTGACGA GTCC AATGTCGGAGTGATTATCGTTGGCAGAGTG GGAC GAAACAGC

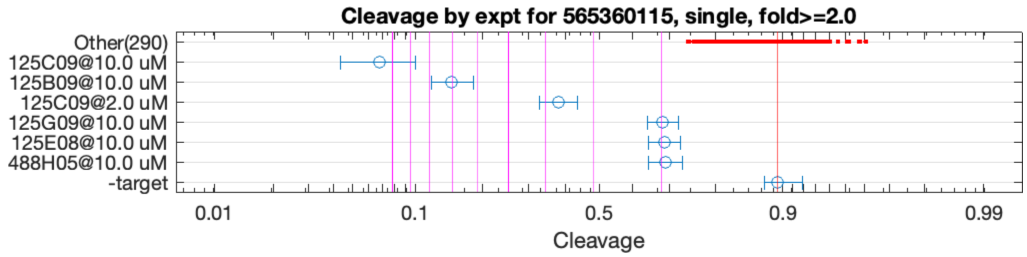

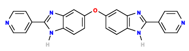

125C09

v>8.2 s=10.7

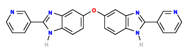

125B09

v>6.5 s=7.0

565360436 GCTGTC ACTGGA AGGCATGTCTTTGAGTAGCCAAAACTGGAA TCCGGT CTGACGA GTCC GATACGTTGGTGAGTAGATGCATGTGCCTG GGAC GAAACAGC

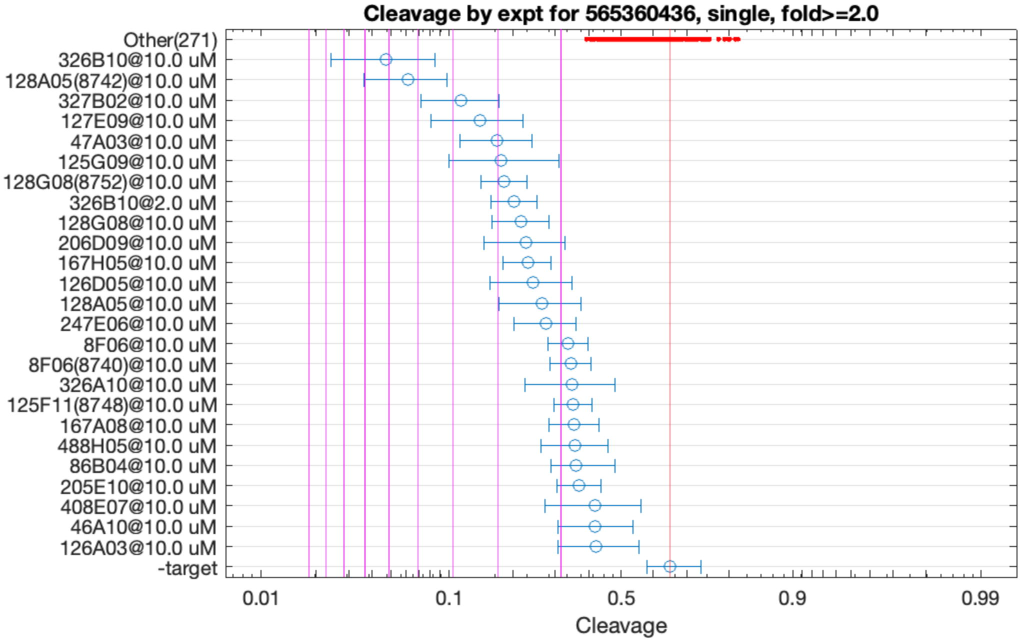

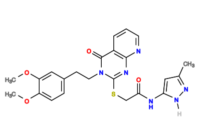

326B10

v>3.0 s=6.1

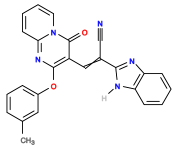

128A05

v>2.2 s=5.4

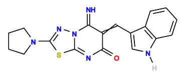

327B02

v>2.2 s=3.8

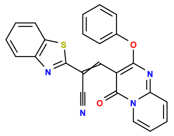

127E09

v>1.8 s=3.3

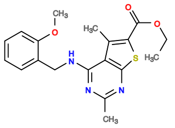

47A03

v>1.8 s=3.0

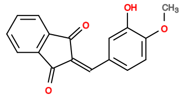

125G09

v>1.8 s=2.9

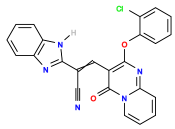

128G08

v>2.4 s=2.9

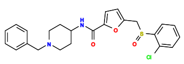

487E02

v>2.6

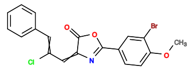

567G08

v>2.5

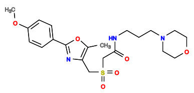

285B11

v>2.5

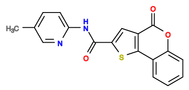

206D09

v>2.6 s=2.5

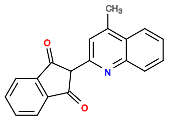

167H05

v>1.9 s=2.5

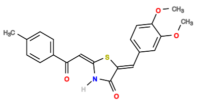

126D05

v>2.5 s=2.4

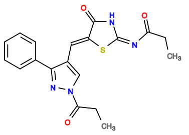

47A11

v>2.4

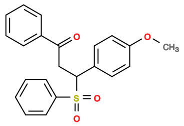

47A09

v>2.4

565360460 GCTGTC ACTGGA AGGTACGTCTTTGAGTAGCCAAAACTGGAA TCCGGT CTGACGA GTCC GATACGTTGGTGAGTAGATGCATGTGCCTG GGAC GAAACAGC

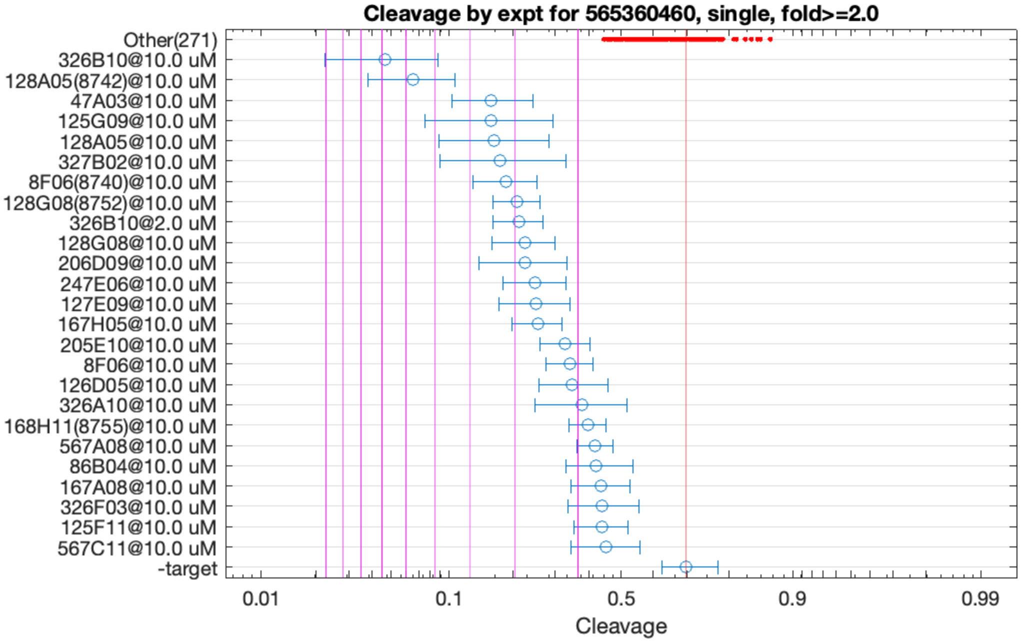

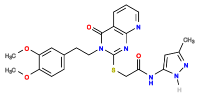

326B10

v>3.2 s=6.7

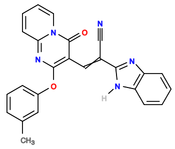

128A05

v>2.1 s=5.6

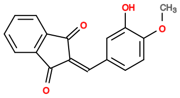

125G09

v>2.0 s=3.5

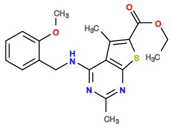

47A03

v>1.9 s=3.4

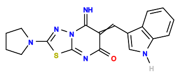

327B02

v>2.3 s=3.3

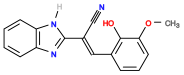

8F06

v>1.8 s=3.1

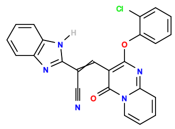

128G08

v>2.5 s=3.0

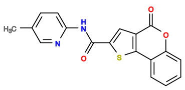

206D09

v>2.3 s=2.8

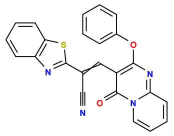

127E09

v>1.8 s=2.6

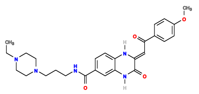

247E06

v>2.1 s=2.6

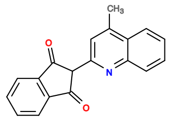

167H05

v>2.0 s=2.6

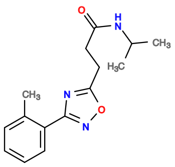

405C07

v>2.5

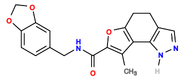

446E04

v>2.5

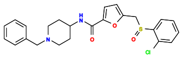

487E02

v>2.5

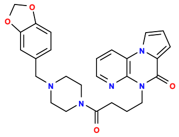

485A07

v>2.4

565366116 GCTGTC ACTGGA GGAAGAATTCAAGTGTGAAAAGGTTGATCA TCCGGT CTGACGA GTCC GCAAACCTCAACGCATAACACTTGTGACTG GGAC GAAACAGC

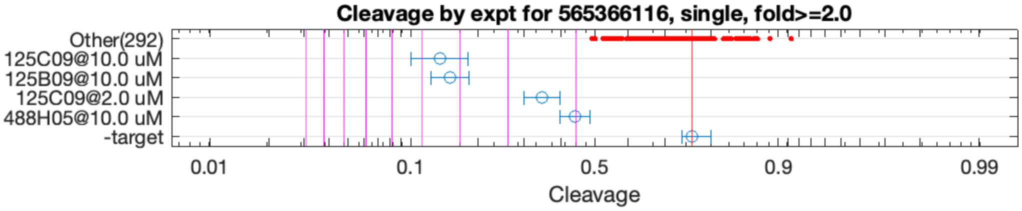

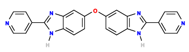

125C09

v>4.9 s=4.5

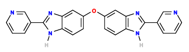

125B09

v>4.3 s=4.2

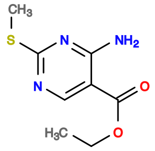

488H05

v>1.5 s=2.0

565370758 GCTGTC AC TGGATTCTGGTTAGCTGCAACTGGAAATTCT GT CTGAAGA GTCC TTGGAAAGGTGGACGAAACAGCTGCGTGCC GGAC GAAACAGC

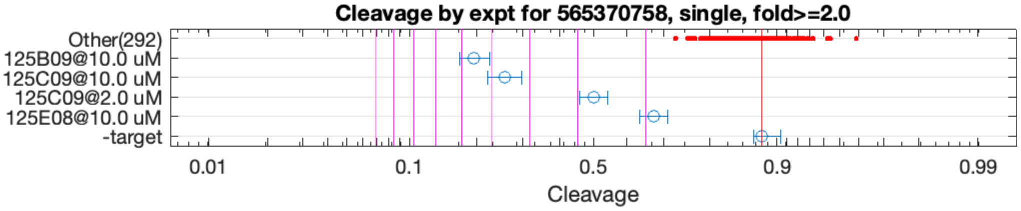

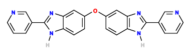

125B09

v>4.4 s=5.6

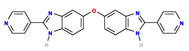

125C09

v>4.3 s=4.6

565372067 GCTGTC ACTGG ATTTACTGGATTTTCGTG TCAGT CTGACGA GTCT TTGT GGAC GAAACAGC

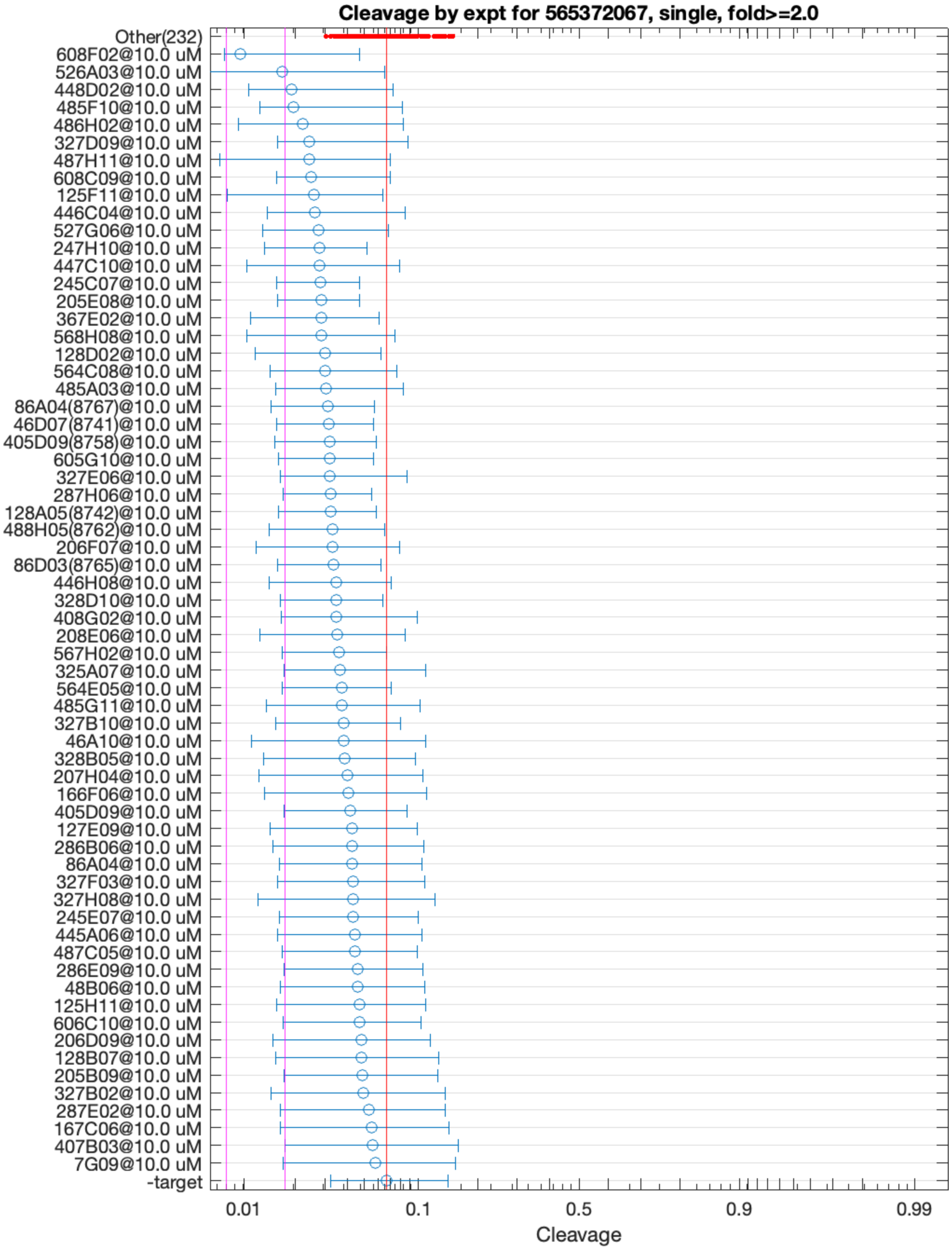

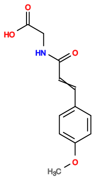

608F02

v>0.7 s=2.7

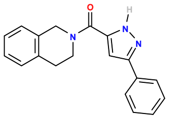

526A03

v>0.6 s=2.0

565378920 GCTGTC ACTG GATTTTCAGTTCGACGAACACTGGATACCGTAAA CAGT CTGAAGA GTCC AAATCTGCCGATTTTCGCGTGGGTGCGGG GGAC GAAACAGC

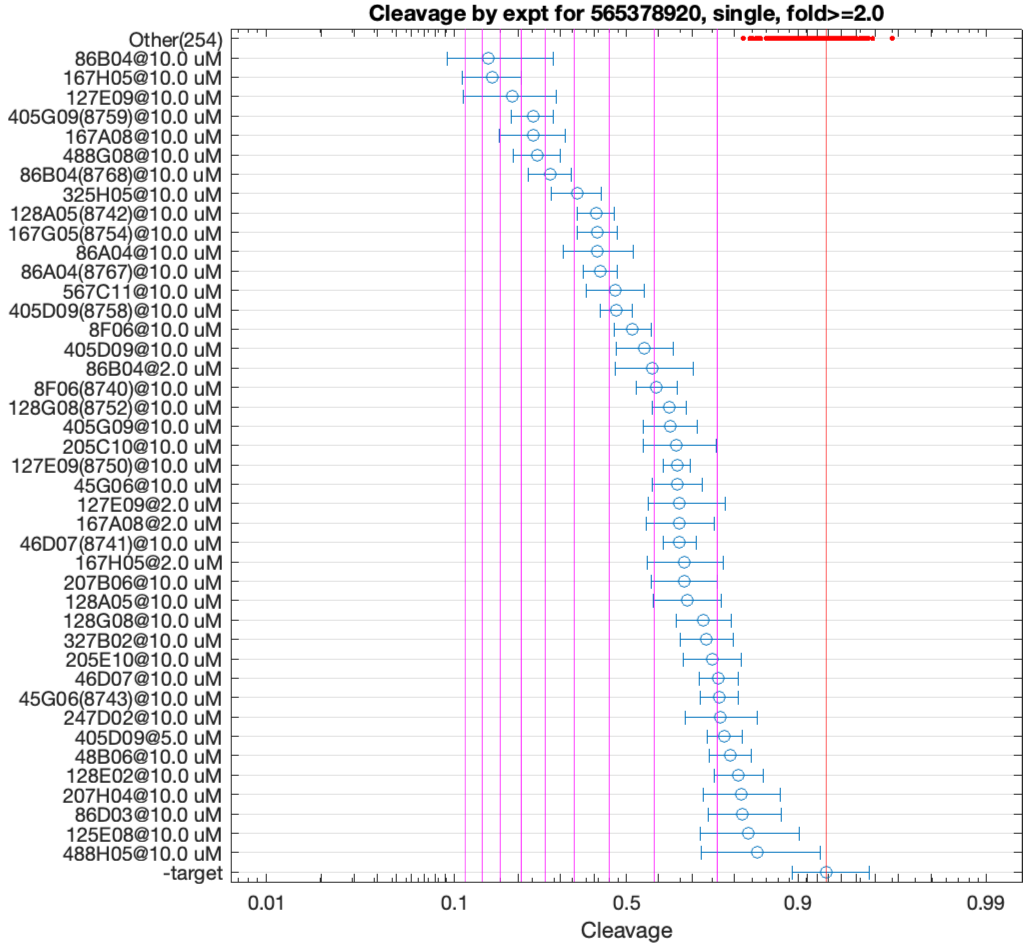

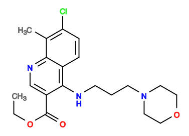

86B04

v>4.0 s=8.6

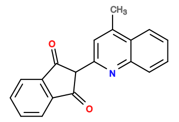

167H05

v>3.2 s=8.3

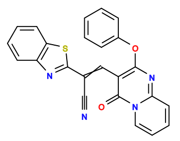

127E09

v>3.0 s=7.3

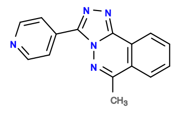

405G09

v>3.7 s=6.7

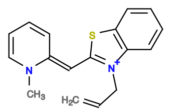

167A08

v>2.4 s=6.5

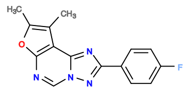

488G08

v>3.2 s=6.2

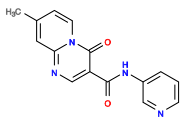

325H05

v>4.3 s=5.0

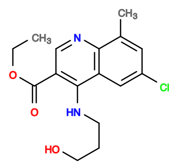

86A04

v>2.9 s=4.4

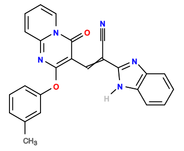

128A05

v>2.5 s=4.4

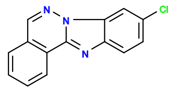

167G05

v>2.9 s=4.3

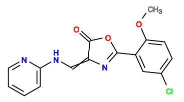

567C11

v>2.7 s=3.8

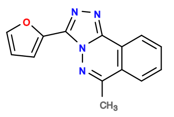

405D09

v>2.6 s=3.8

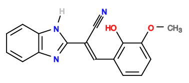

8F06

v>2.3 s=3.4

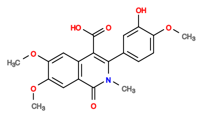

206H06

v>3.2

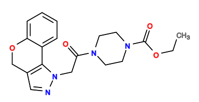

328C11

v>3.1

565380623 GCTGTC ACTGG ATTTTCCAGGATCACTGGATGCATGACCATATTTGGTGTGCCGAGCGGCA CCAGT CTGACGA GTCT GTGGCG GGAC GAAACAGC

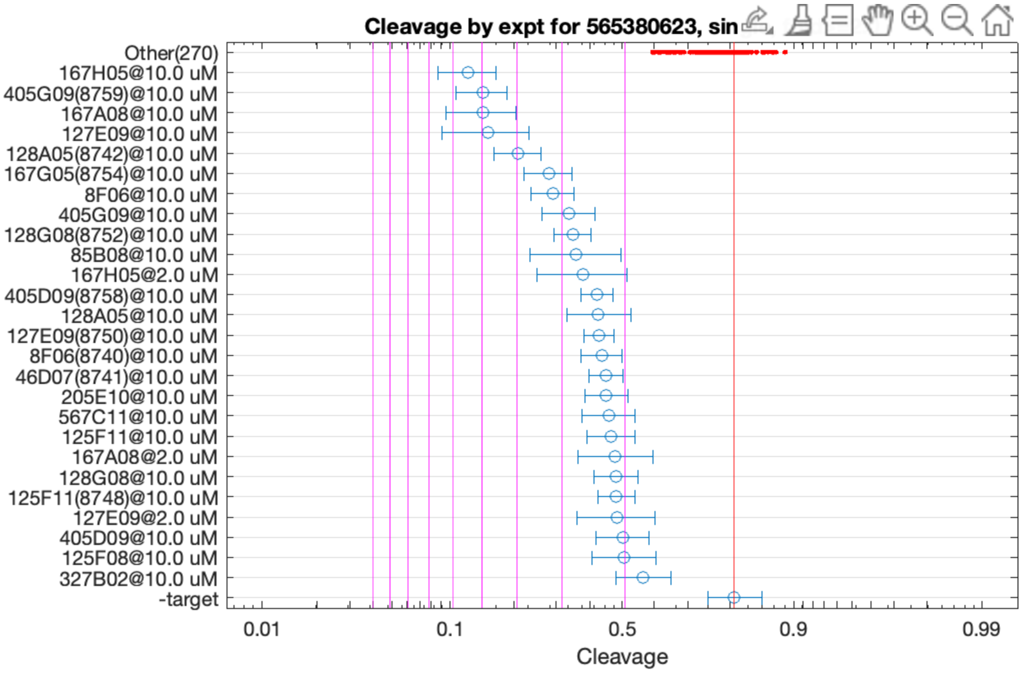

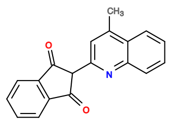
167H05

v>2.4 s=5.5

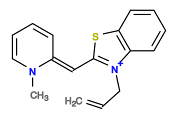
167A08

v>2.5 s=5.0

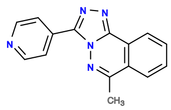
405G09

v>3.2 s=4.9

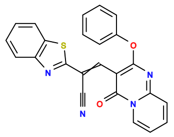
127E09

v>2.6 s=4.8

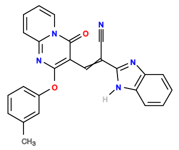
128A05

v>2.4 s=4.0

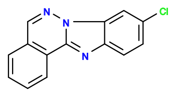
167G05

v>2.5 s=3.3

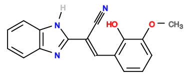
8F06

v>2.0 s=3.2

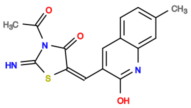
85B08

v>2.4 s=2.8

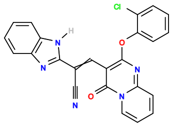
128G08

v>2.7 s=2.8

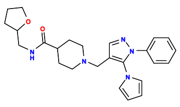
446C03

v>2.6

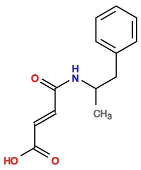
6E06

v>2.6

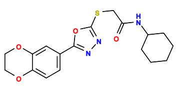
246A07

v>2.5

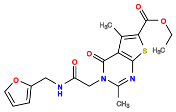
206G08

v>2.5

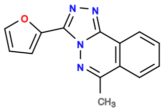
405D09

v>2.2 s=2.4

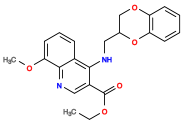
205E10

v>2.1 s=2.2

565385129 GCTGTC ACTG GATTTTGGTGGATGACGAAACACTGGATACCGTAAA CAGT CTGAAGA GTCC AAATCTGCTCGATGTCGTGTGGGTGCGGG GGAC GAAACAGC

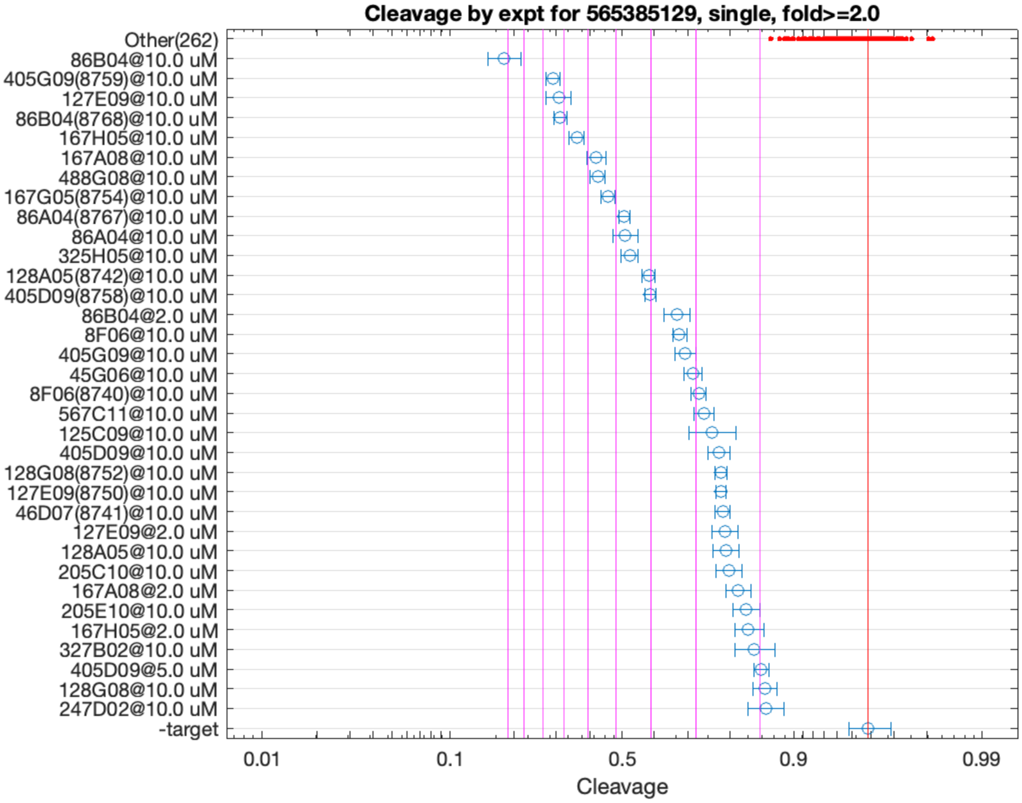

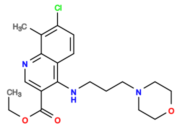

86B04

v>4.4 s=10.2

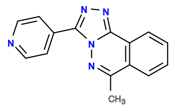

405G09

v>3.7 s=7.4

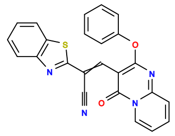

127E09

v>2.9 s=7.2

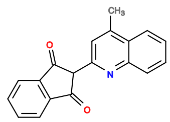

167H05

v>3.1 s=6.5

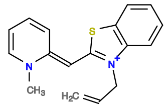

167A08

v>2.4 s=5.7

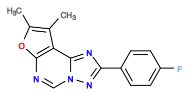

488G08

v>2.9 s=5.6

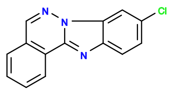

167G05

v>3.2 s=5.3

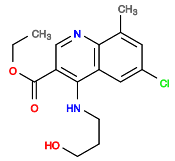

86A04

v>3.3 s=4.8

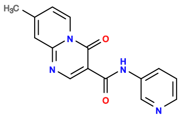

325H05

v>4.4 s=4.6

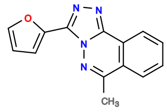

405D09

v>2.7 s=4.0

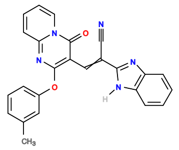

128A05

v>2.4 s=4.0

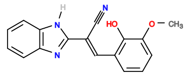

8F06

v>2.2 s=3.3

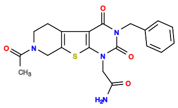

327A11

v>3.2

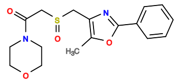

285E02

v>3.2

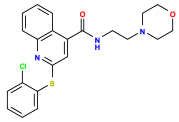

208H11

v>3.1

565388300 GCTGTC ACTGGA ATGTGGCGTGAGTAAGGTGCGGTAAGATCA TCTGGT CTGATGA GTCC GTTGAACCGTGCTTGAGTAGCGTCGACTGGG GGAC GAAACAGC

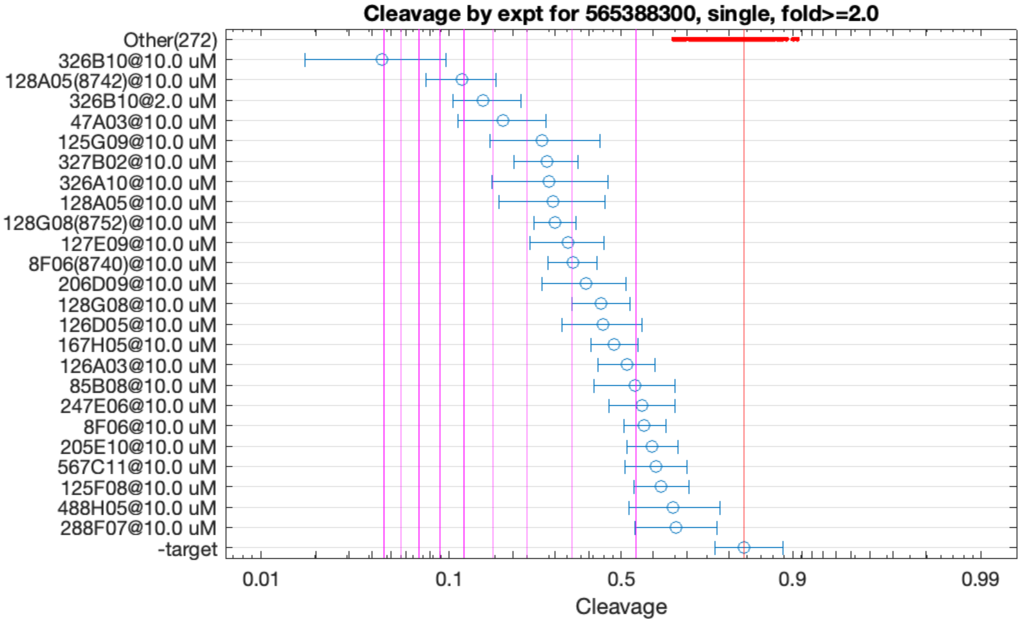

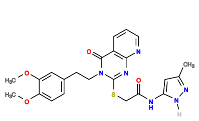

326B10

v>3.9 s=10.4

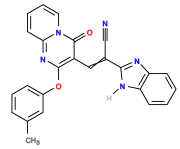

128A05

v>2.4 s=6.2

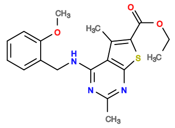

47A03

v>2.1 s=4.7

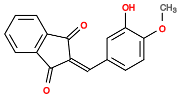

125G09

v>2.1 s=3.7

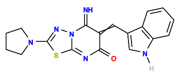

327B02

v>2.3 s=3.5

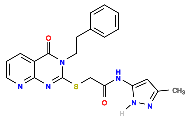

326A10

v>3.0 s=3.4

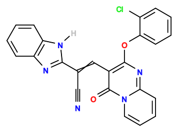

128G08

v>2.5 s=3.3

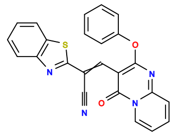

127E09

v>1.6 s=3.1

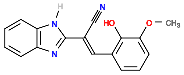

8F06

v>1.8 s=3.0

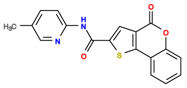

206D09

v>3.3 s=2.7

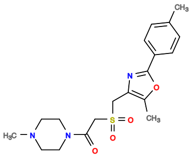

285A10

v>2.7

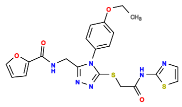

205E07

v>2.6

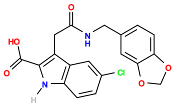

245E04

v>2.6

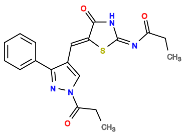

47A11

v>2.6

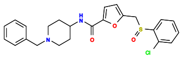

487E02

v>2.6

565411466 GCTGTC ACTGGA GACTGGTG TCCAGT CCGATGA GTCT ACTGCCTCTGCACTATCTGCAAGCGTACCG GGAC GAAACAGC

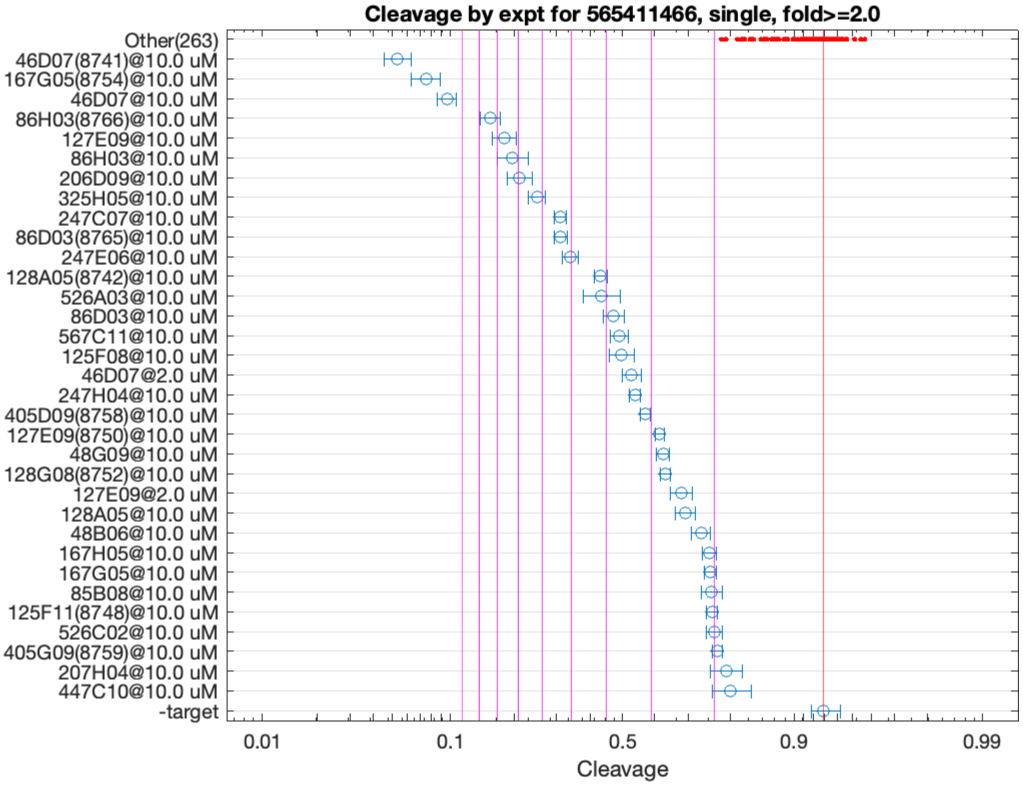

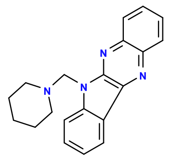

46D07

v>8.9 s=15.1

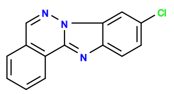

167G05

v>5.7 s=12.5

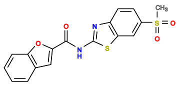

86H03

v>4.6 s=8.4

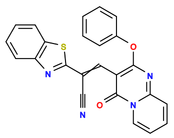

127E09

v>2.3 s=7.7

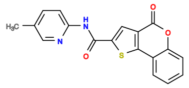

206D09

v>6.0 s=6.9

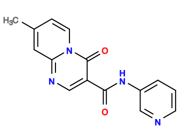

325H05

v>5.3 s=6.2

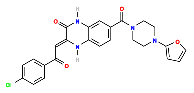

247C07

v>4.4 s=5.3

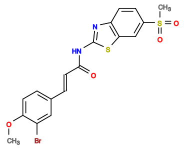

86D03

v>5.0 s=5.3

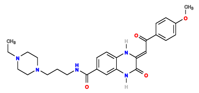

247E06

v>5.5 s=5.0

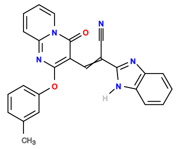

128A05

v>2.4 s=4.1

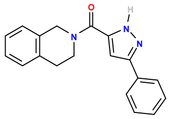

526A03

v>2.3 s=4.1

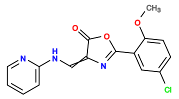

567C11

v>3.1 s=3.7

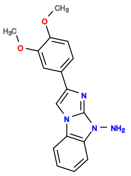

125F08

v>3.1 s=3.6

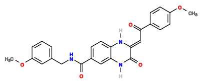

247H04

v>2.9 s=3.3

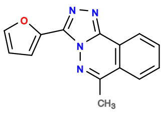

405D09

v>2.6 s=3.1

565476652 GCTGTC ACTGGA GGAAGAGG TCCGGT CTGATGA GTCC GAAAACAAGTCAGCGCTAGGGAGATCGGTG GGAC GAAACAGC

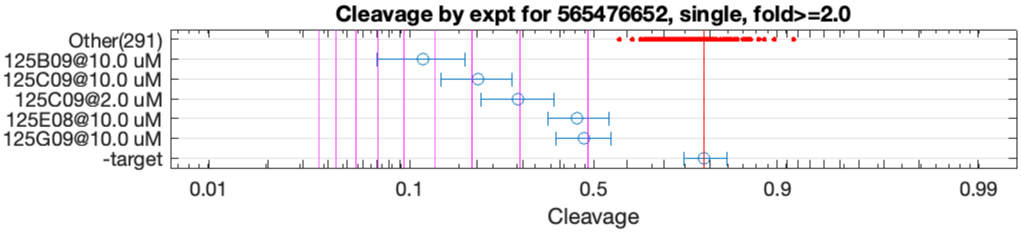

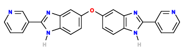

125B09

v>4.3 s=5.4

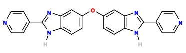

125C09

v>4.2 s=3.8

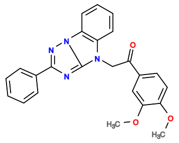

125E08

v>1.4 s=2.1

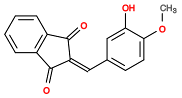

125G09

v>1.6 s=2.1

565491775 GCTGTC ACTGGA AACTCTCTGAATGAAGCGGGTATCGCAATG TCTGGT CTGATGA GTCC GATGCGGTACAGGTCAGGGTTGGATGTG GGAC GAAACAGC

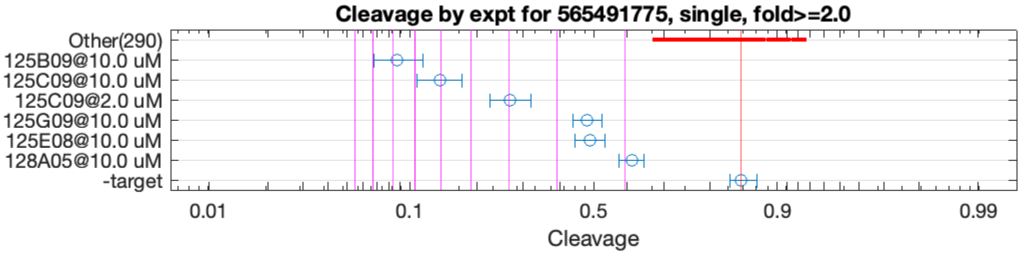

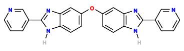

125B09

v>6.7 s=7.8

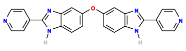

125C09

v>6.5 s=6.0

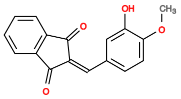

125G09

v>1.7 s=2.5

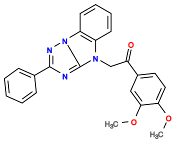

125E08

v>1.3 s=2.5

565493161 GCTGTC ACTGGAA ATTGCAACCAAATGCAGAAAACCTCAGA TTCCGGT CCGATGA GTCCT TGGGGT GGGAC GAAACAGC

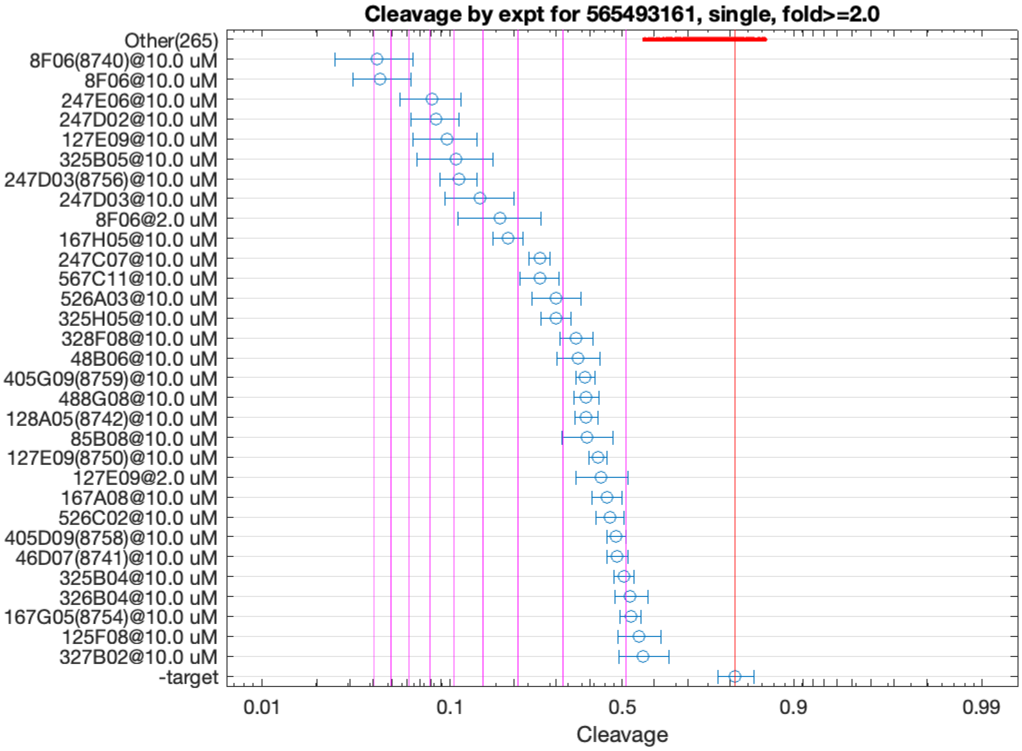

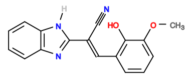

8F06

v>4.1 s=10.0

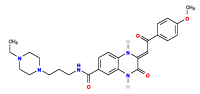

247E06

v>4.1 s=7.0

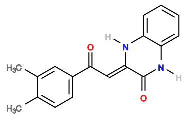

247D02

v>5.1 s=6.7

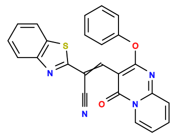

127E09

v>3.1 s=6.4

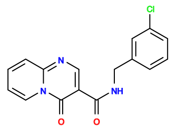

325B05

v>4.0 s=6.0

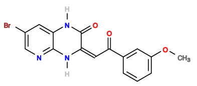

247D03

v>4.1 s=5.9

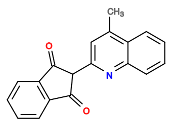

167H05

v>3.1 s=4.3

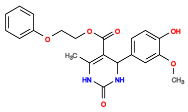

5A04

v>4.2

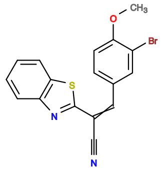

8C05

v>4.1

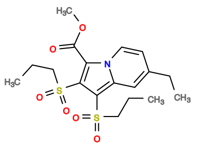

85G06

v>4.1

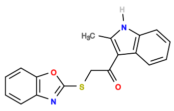

126E02

v>4.0

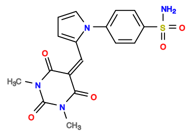

46A07

v>4.0

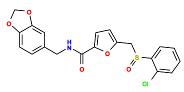

486G11

v>3.9

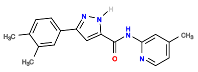

526H04

v>3.9

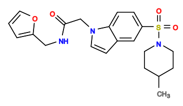

528C07

v>3.9

565513272 GCTGTC ACTGG AAAAACACTGGATGCATGACCATATTTGGTGTGCCGAGCTGCA CCAGT CTGATGA GTTC GTGGCG GGAC GAAACAGC

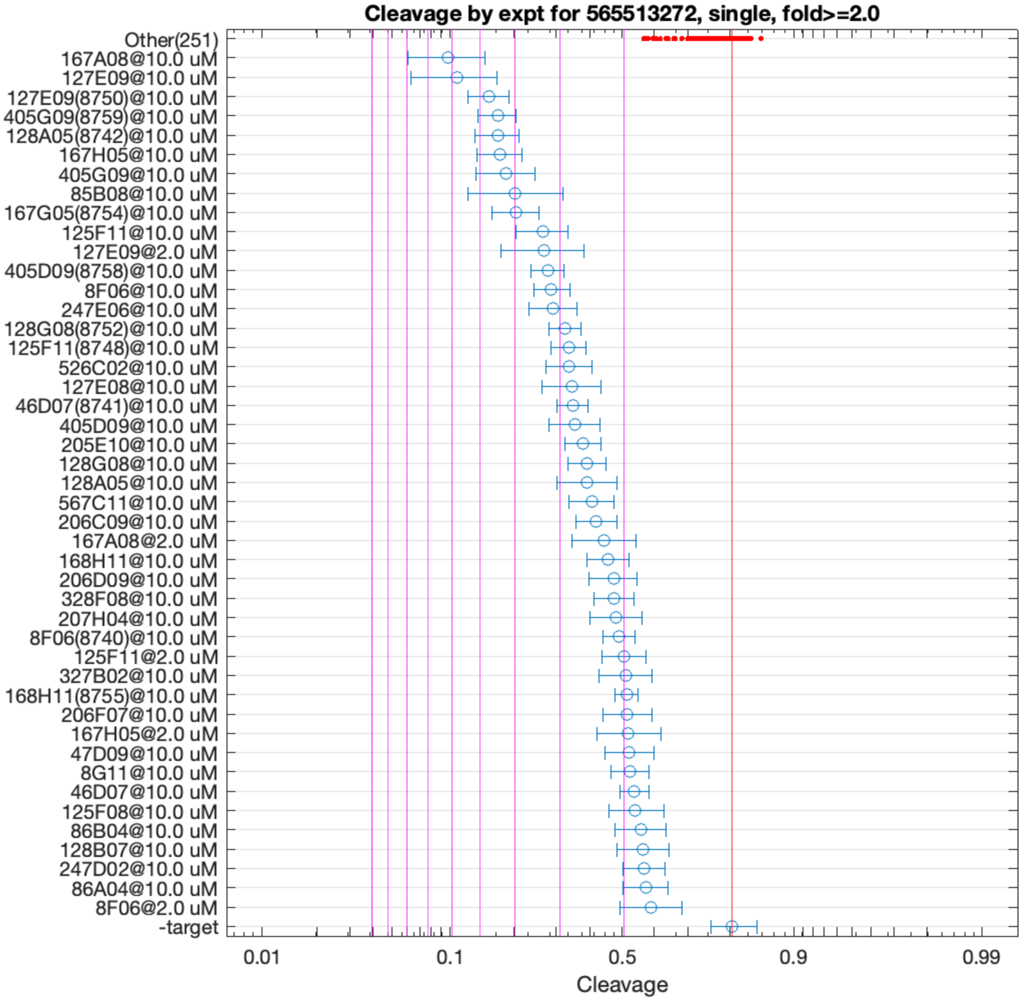

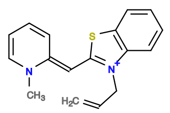

167A08

v>3.1 s=6.1

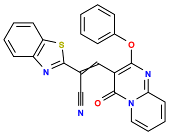

127E09

v>3.4 s=5.9

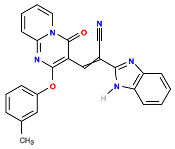

128A05

v>2.6 s=4.5

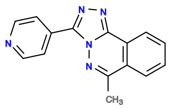

405G09

v>3.6 s=4.5

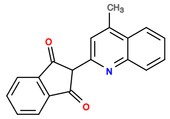

167H05

v>2.8 s=4.3

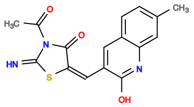

85B08

v>2.6 s=4.0

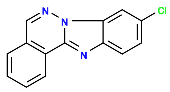

167G05

v>2.9 s=3.9

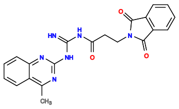

125F11

v>2.3 s=3.4

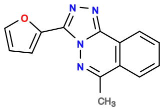

405D09

v>2.5 s=3.3

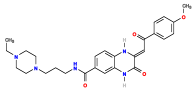

247E06

v>2.5 s=3.2

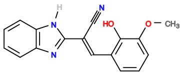

8F06

v>2.3 s=3.2

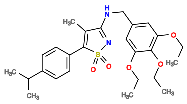

447C04

v>3.0

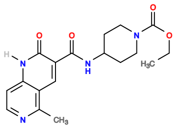

608B07

v>3.0

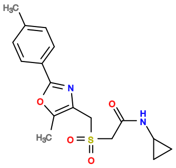

285D06

v>3.0

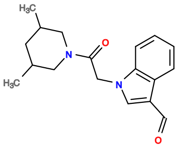

606E08

v>2.9

565547677 GCTGTC ACTGGA GGGGACGA TCCGGT CTGATGA GTCC GTATCGAGGAAGAATTCGCGATATCCACAG GGAC GAAACAGC

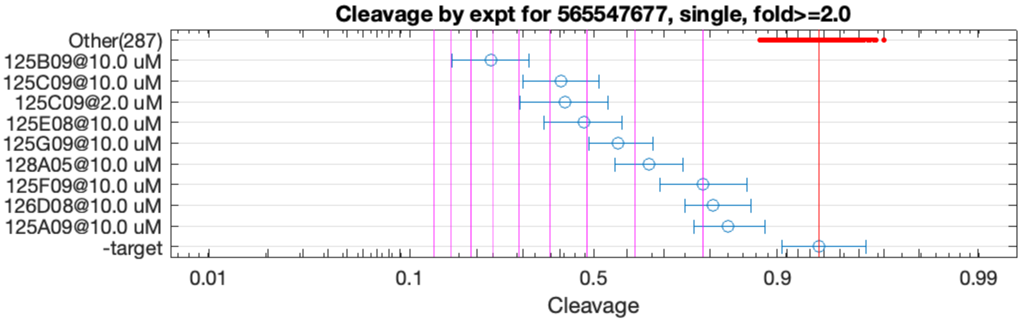

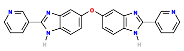
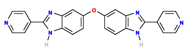
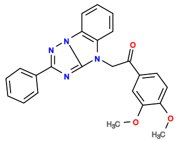
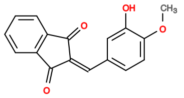
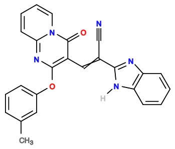

125B09 125C09 125E08 125G09 128A05

v>6.4 s=7.1 v>5.9 s=4.7 v>1.7 s=4.0 v>2.6 s=3.3 v>1.0 s=2.7

565587233 GCTGTC ACTGGA TTTTCCTGCAATGATCTGTGGGGAAACGGC TCCGGT CTGATGA GTTC GTGGCG GGAC GAAACAGC

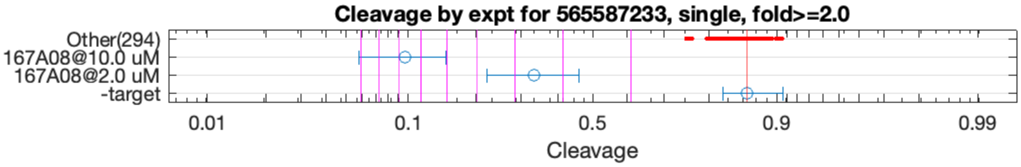

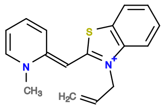

167A08

v>2.4 s=7.8

565658419 GCTGTC ACTGGA CATTCTGAGCATTTGGGTTGAATTGGCGAA TCCGGT CTGACGA GTCCTGTA GTCGGTTACCGTGAAGCTCGGG TGTGGGAC GAAACAGC

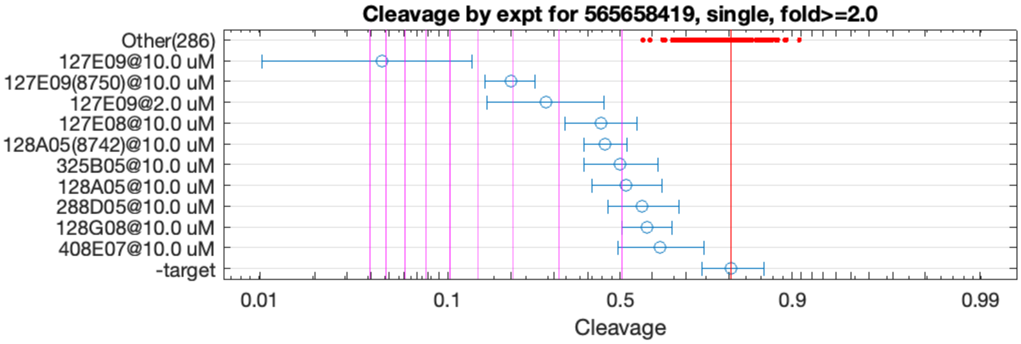

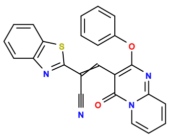
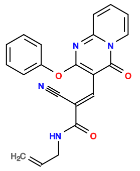
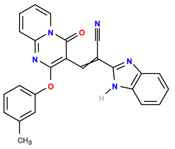
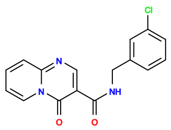

127E09 127E08 128A05 325B05

v>2.6 s=9.0 v>1.4 s=2.3 v>1.4 s=2.2 v>1.2 s=2.0

565658927 GCTGTC ACTGGA ATTTTCTGGTTCAACTGGAATTATGCCTTCAAAGGGTGCCGATCTAA TCTGGT CTGATGA GTCT GTGGCG GGAC GAAACAGC

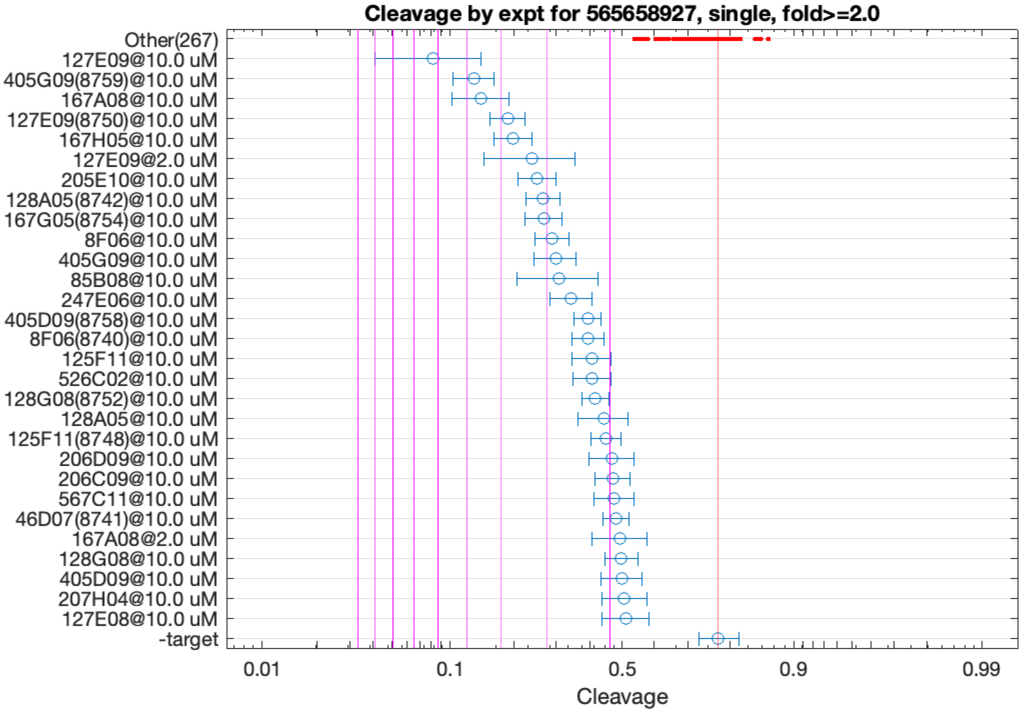

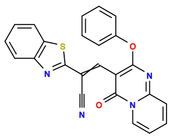

127E09

v>2.7 s=6.2

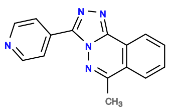

405G09

v>2.9 s=4.8

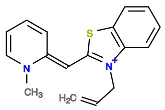

167A08

v>2.2 s=4.5

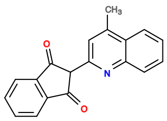

167H05

v>2.1 s=3.7

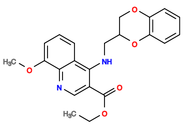

205E10

v>2.3 s=3.1

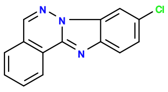

167G05

v>2.2 s=3.1

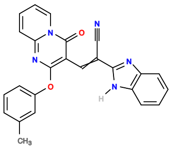

128A05

v>2.0 s=3.0

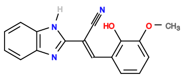

8F06

v>1.7 s=2.9

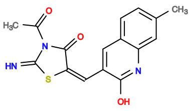

85B08

v>2.1 s=2.8

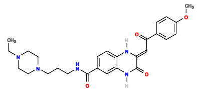

247E06

v>2.1 s=2.6

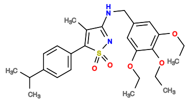

447C04

v>2.3

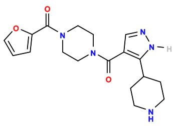

526D09

v>2.3

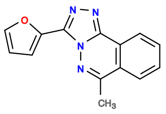

405D09

v>2.0 s=2.3

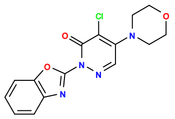

248D09

v>2.3

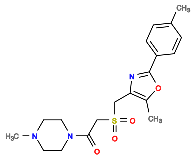

285A10

v>2.3

565658937 GCTGTC ACTGGA ACTGGATCCAATCGCATGAGTGTGAATGGGTGTGCTGAGCTGC TCTAGT CTGACGA GTCC GTGGCG GGAC GAAACAGC

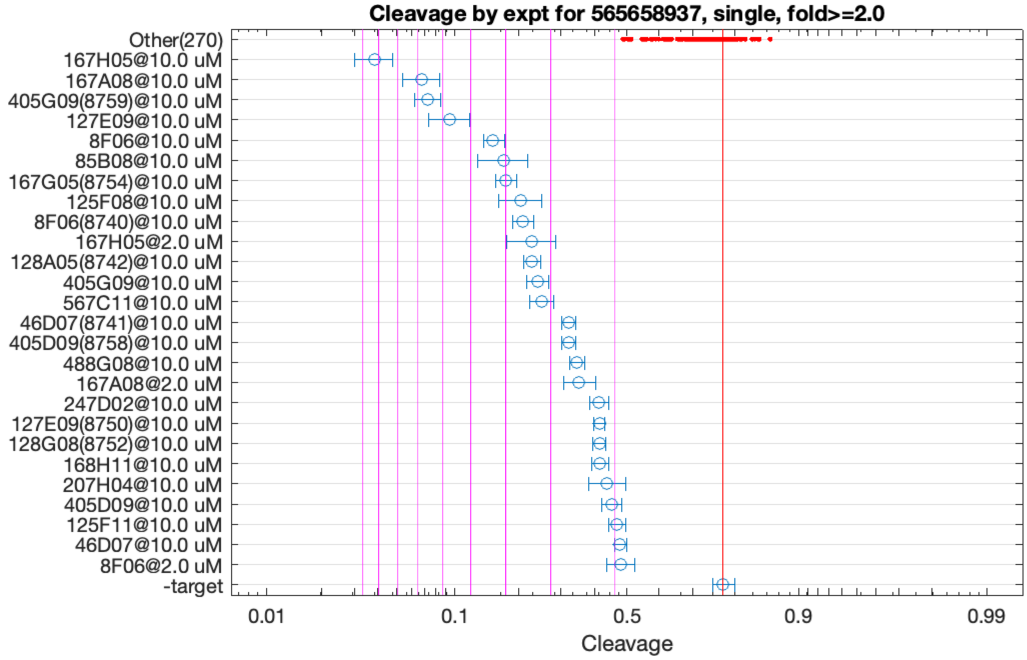

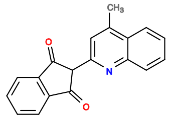

167H05

v>2.9 s=9.3

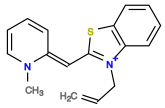

167A08

v>2.9 s=6.8

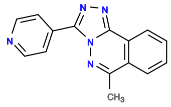

405G09

v>3.9 s=6.6

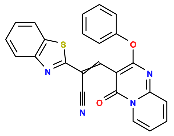

127E09

v>2.9 s=5.8

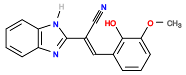

8F06

v>2.6 s=4.4

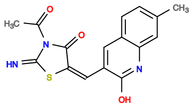

85B08

v>3.1 s=4.0

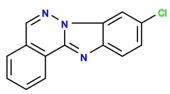

167G05

v>3.1 s=4.0

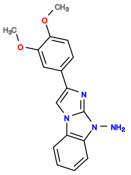

125F08

v>3.4 s=3.6

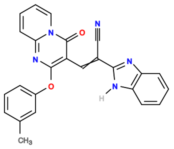

128A05

v>2.4 s=3.4

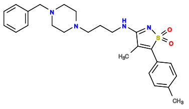

446E08

v>3.3

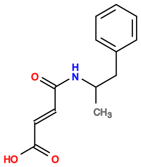

6E06

v>3.3

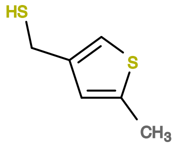

488C07

v>3.2

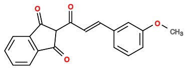

6C06

v>3.2

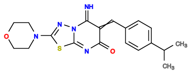

327H05

v>3.2

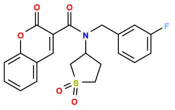

408E03

v>3.2

565662496 GCTGTC ACTGGAC GTGGCGA GTTCGGT CTGACGA GTCC ATACATGTACTGTAAAGACAGGAGGTGGTG GGAC GAAACAGC

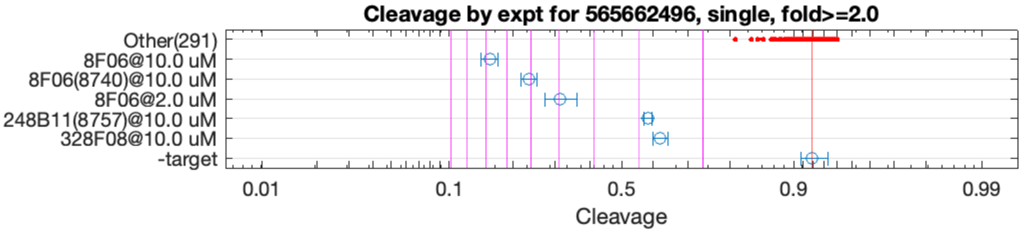

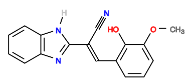

8F06

v>3.1 s=7.8

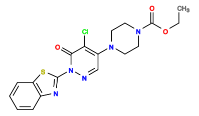

248B11

v>1.1 s=2.8

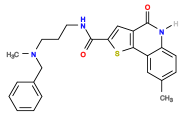

328F08

v>1.4 s=2.6

565663129 GCTGTC ACTGGA ATTTTCTGGTTCAACTGGAATTATGCCTTTCGAGGGTTGCCGATCTAA TCTGGT CTGACGA GTCT GTGGCG GGAC GAAACAGC

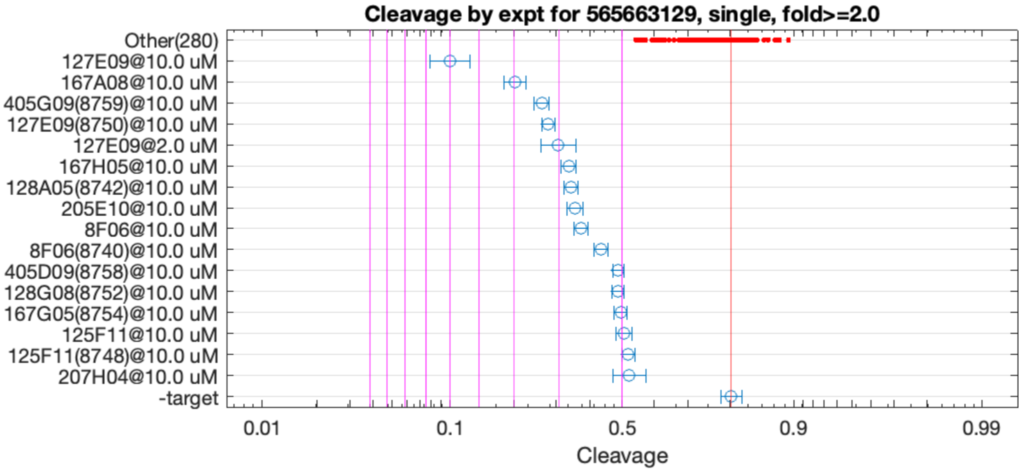

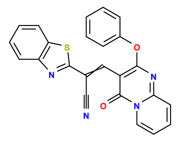

127E09

v>2.5 s=6.0

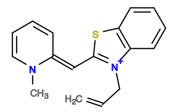

167A08

v>2.0 s=4.0

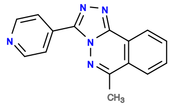

405G09

v>2.4 s=3.3

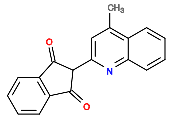

167H05

v>2.1 s=2.8

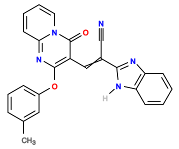

128A05

v>1.9 s=2.8

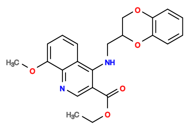

205E10

v>1.8 s=2.7

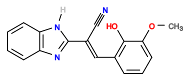

8F06

v>1.8 s=2.6

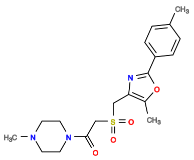

285A10

v>2.3

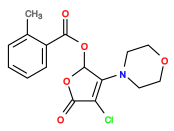

88E07

v>2.3

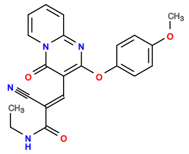

128G03

v>2.2

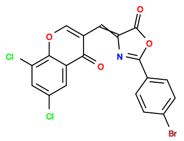

564H11

v>2.2

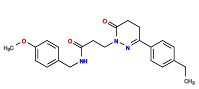

607A08

v>2.2

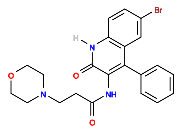

125A10

v>2.2

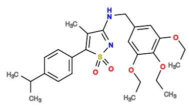

447C04

v>2.2

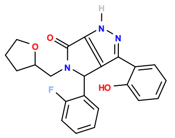

405B11

v>2.2

565675752 GCTGTC ACTG GAATGCGTAAA CAGT CTGACGA GTCC AAATCTGCTCGATGTCGTGTAGGTGCGTG GGAC GAAACAGC

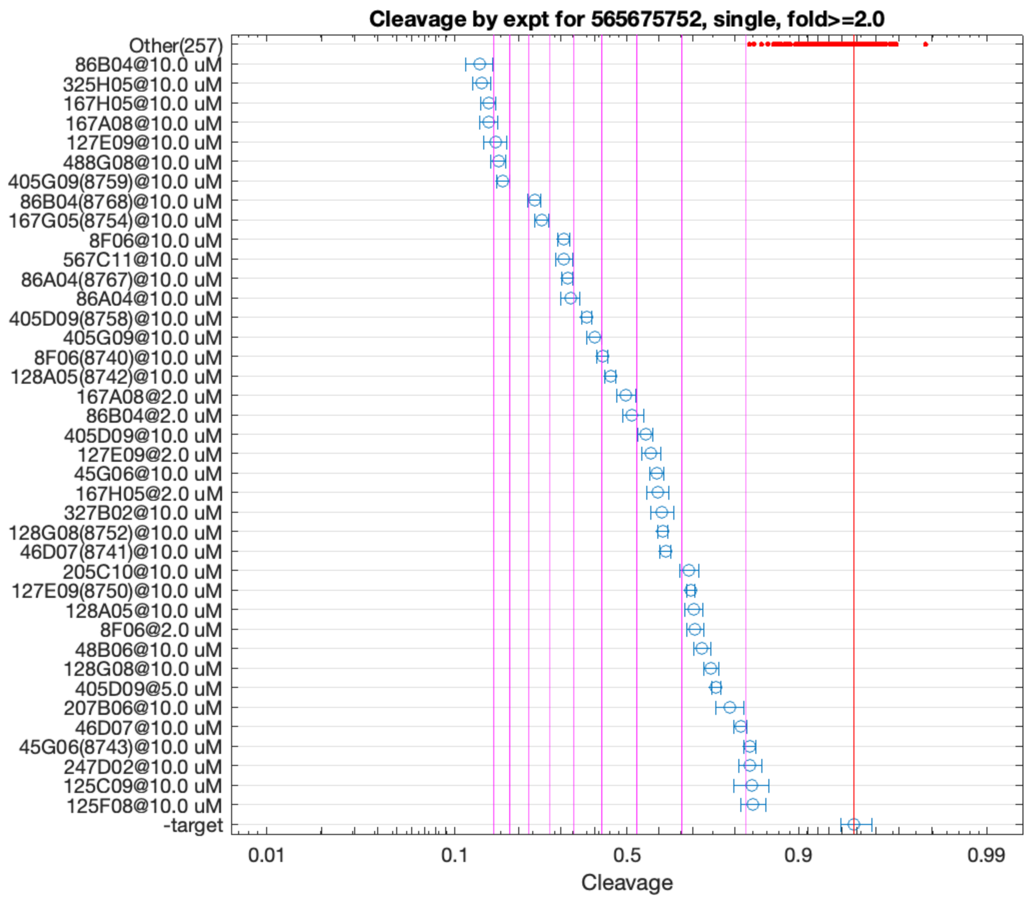

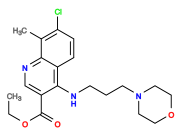

86B04

v>6.4 s=11.0

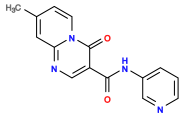

325H05

v>11.5 s=10.8

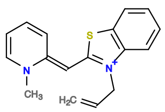

167A08

v>5.2 s=10.4

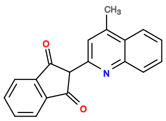

167H05

v>5.8 s=10.4

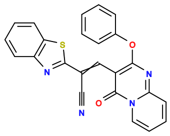

127E09

v>4.5 s=10.0

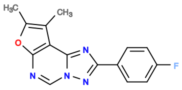

488G08

v>5.3 s=9.7

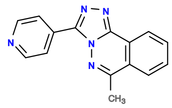

405G09

v>6.6 s=9.4

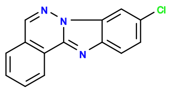

167G05

v>5.4 s=7.4

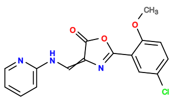

567C11

v>6.4 s=6.4

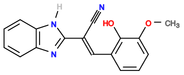

8F06

v>4.0 s=6.4

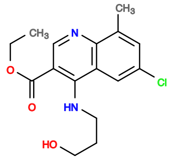

86A04

v>5.1 s=6.3

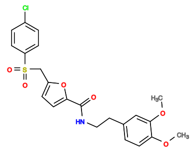

605E11

v>5.9

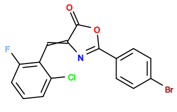

564H10

v>5.6

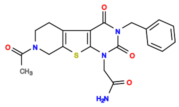

327A11

v>5.6

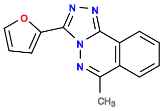

405D09

v>4.6 s=5.5

565703067 GCTGTC ACTG GAATCCGTTAA CAGT CTGACGA GTCC AAATCTGCTCGATTTCGCGTGGGTGCGGG GGAC GAAACAGC

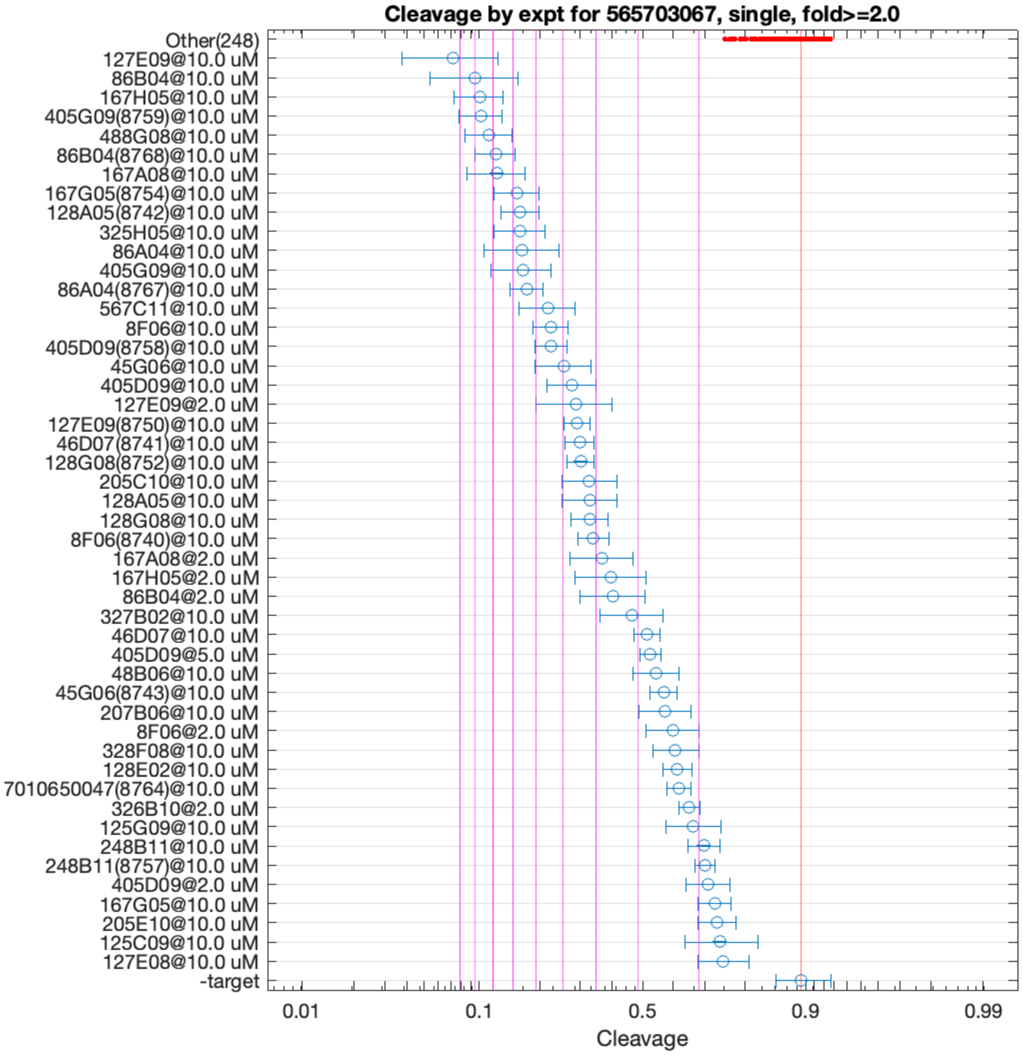

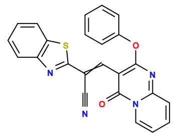

127E09

v>4.9 s=10.3

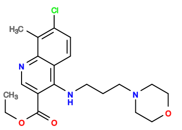

86B04

v>6.7 s=9.0

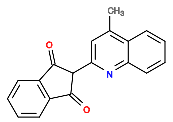

167H05

v>4.8 s=8.8

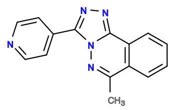

405G09

v>6.4 s=8.6

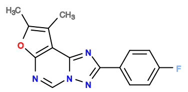

488G08

v>5.1 s=8.2

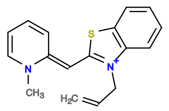

167A08

v>4.6 s=7.7

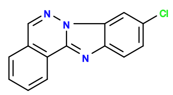

167G05

v>5.5 s=6.8

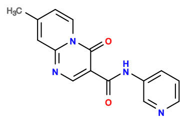

325H05

v>6.4 s=6.7

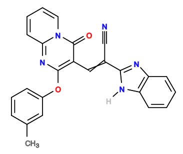

128A05

v>4.6 s=6.6

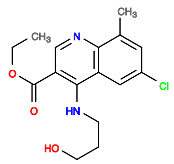

86A04

v>5.1 s=6.5

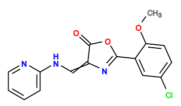

567C11

v>4.9 s=5.6

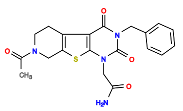

327A11

v>5.4

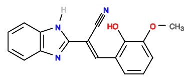

8F06

v>3.8 s=5.4

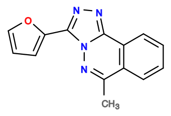

405D09

v>4.7 s=5.4

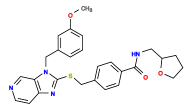

365A02

v>5.2

565703478 GCTGTC ACTGGAA AACATTTTGCGCCGTTCTACTAGAACAC TTCCGGT CCGATGA GTCCTT GCGC GGGGAC GAAACAGC

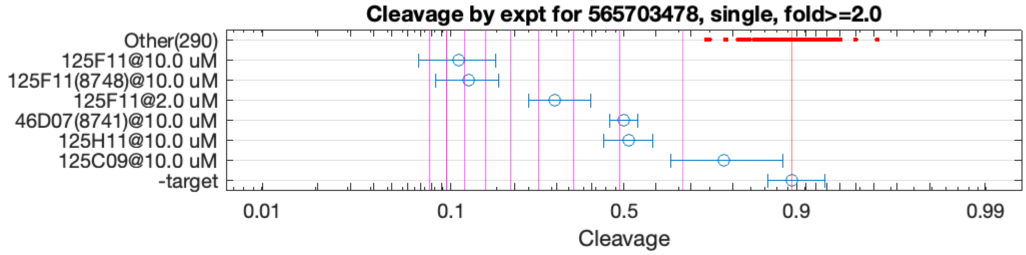

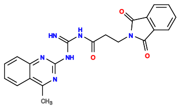

125F11

v>1.7 s=8.2

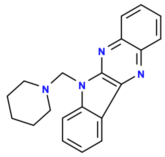

46D07

v>1.0 s=2.9

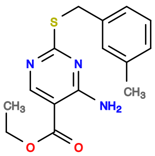

125H11

v>1.4 s=2.8

565707921 GCTGTC ACTGG ATTTTCGACGAATGATGTTGAAAGGATGCATGACCATATTTGGTGTGCCGAGCTGCA CCAGT CTGATGA GTTC GTGGCG GGAC GAAACAGC

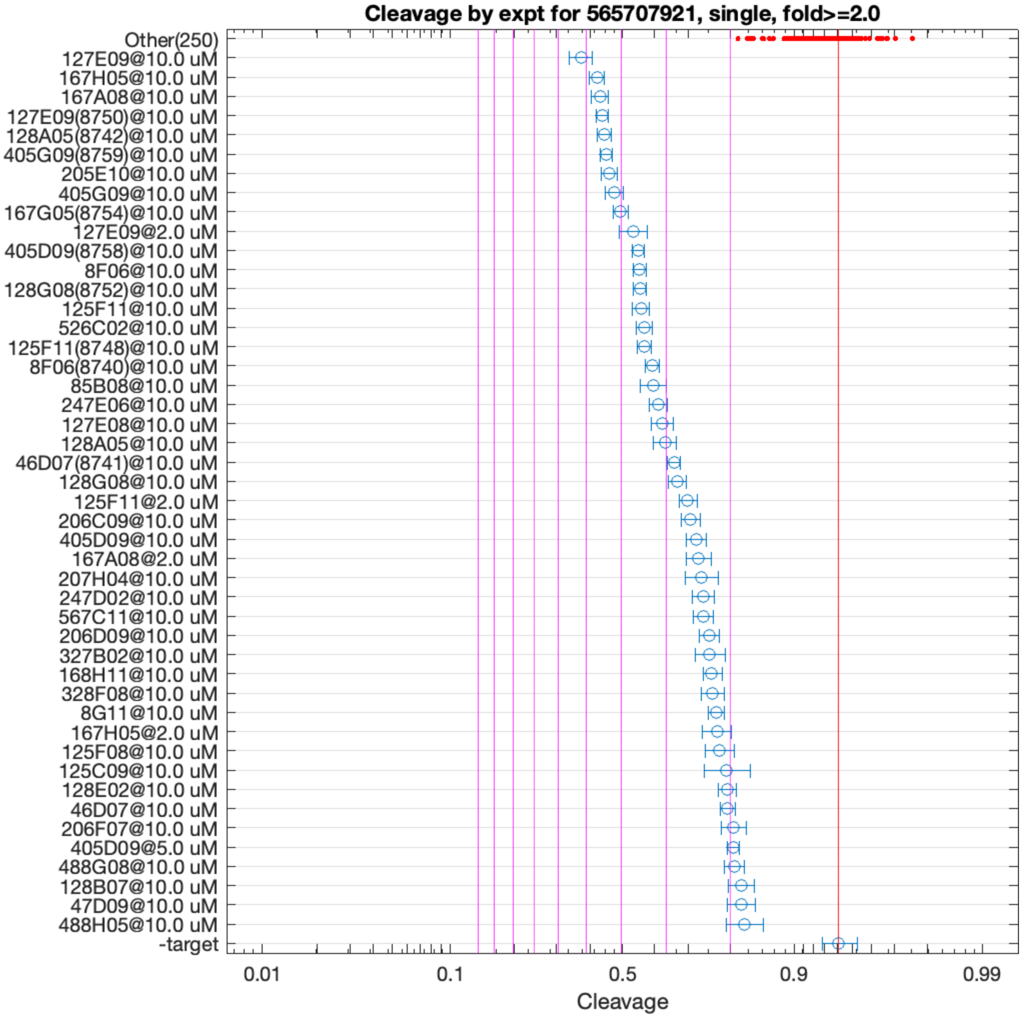

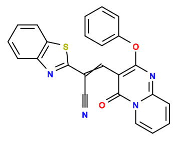

127E09

v>4.2 s=5.2

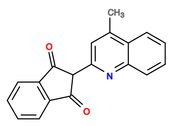

167H05

v>3.7 s=4.7

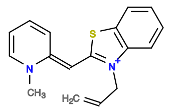

167A08

v>3.5 s=4.6

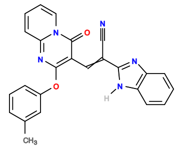

128A05

v>3.4 s=4.5

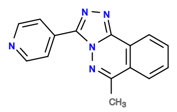

405G09

v>4.4 s=4.4

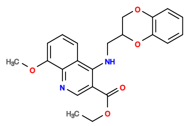

205E10

v>3.9 s=4.3

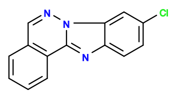

167G05

v>3.6 s=4.0

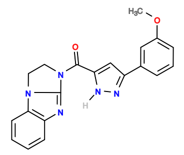

525D11

v>3.9

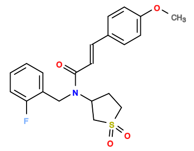

407E11

v>3.9

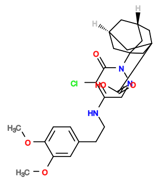

247F08

v>3.9

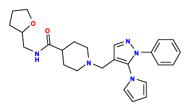

446C03

v>3.8

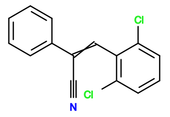

8G09

v>3.8

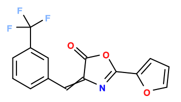

564A05

v>3.8

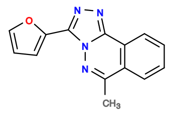

405D09

v>3.4 s=3.6

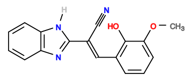

8F06

v>3.0 s=3.6

565730172 GCTGTC ACTGGAA AAAGG TTTCGGT CTGATGA GTTC GATTGATGACGAAACAGCATTGTGCAGCCCG GGAC GAAACAGC

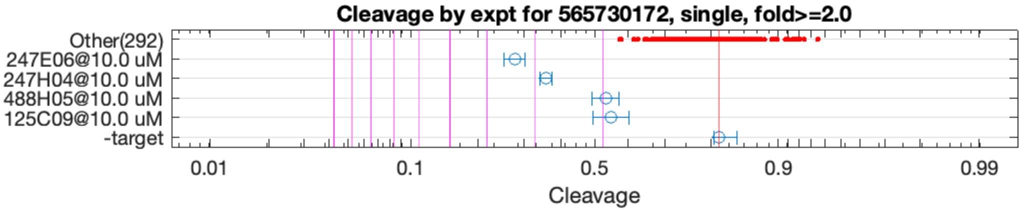

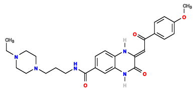

247E06

v>3.6 s=3.4

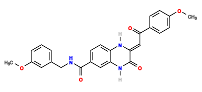

247H04

v>1.3 s=2.8

565736657 GCTGTC ACTGGA AAAAGGACG TCTAGT CTGATGA GTCT ACTGCCTCTGCCCTATCTGCAAGCGTACCG GGAC GAAACAGC

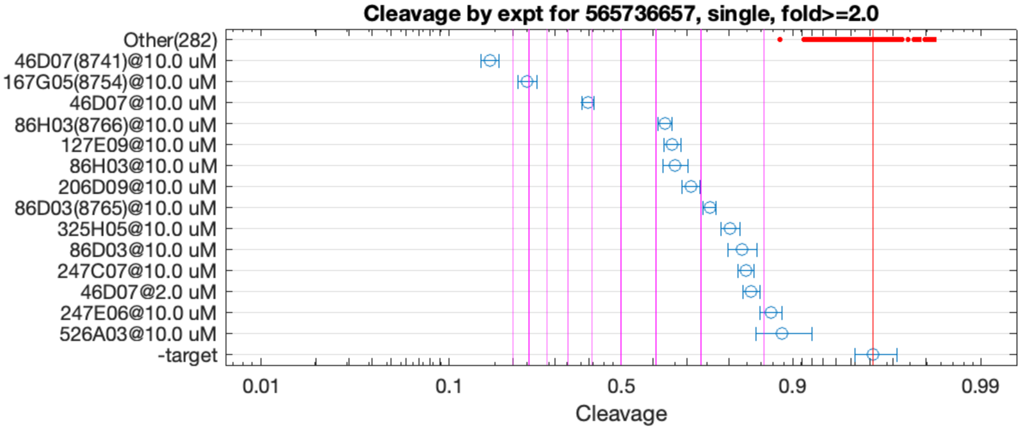

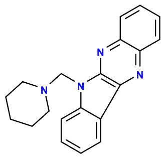

46D07

v>5.0 s=11.6

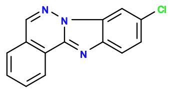

167G05

v>2.4 s=9.0

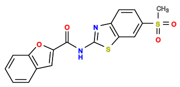

86H03

v>2.0 s=3.8

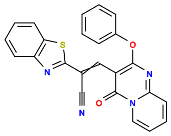

127E09

v>1.5 s=3.6

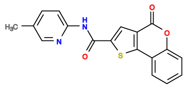

206D09

v>2.6 s=3.2

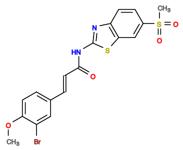

86D03

v>2.9 s=2.8

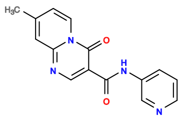

325H05

v>2.2 s=2.5

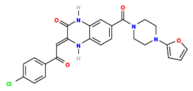

247C07

v>1.9 s=2.2

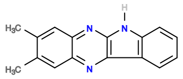

568D02

v>2.0

565770089 GCTGTC ACTGGAT GGT GTCCGGT CCGATGA GTCC GTGTCGAAAAAGTTGATTGCTTCACACCCA GGAC GAAACAGC

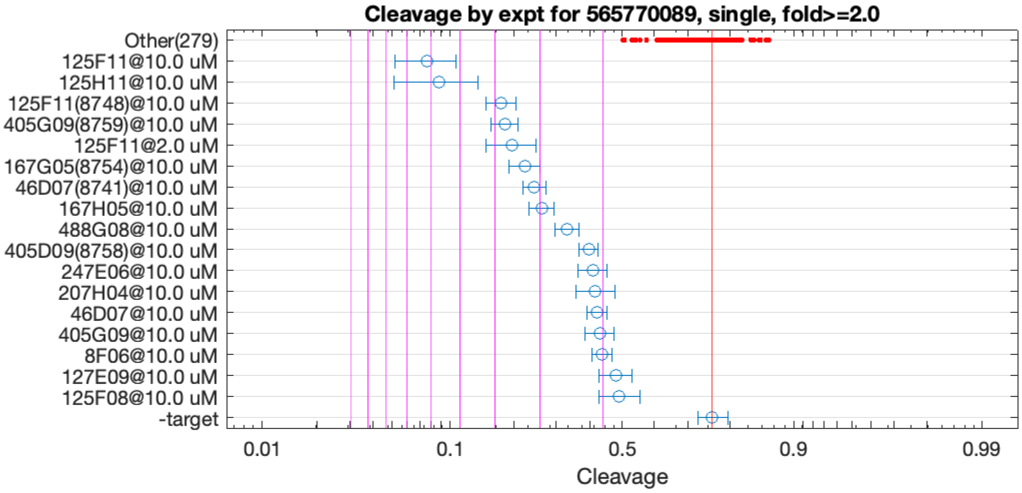

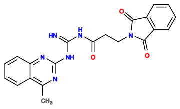

125F11

v>4.2 s=6.2

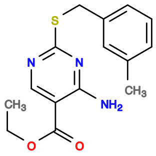

125H11

v>3.7 s=5.7

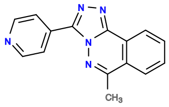

405G09

v>1.9 s=3.7

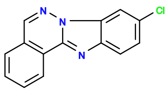

167G05

v>1.8 s=3.3

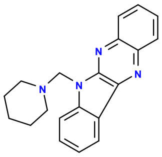

46D07

v>1.8 s=3.1

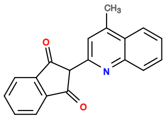

167H05

v>1.7 s=3.0

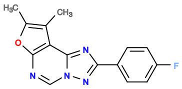

488G08

v>1.5 s=2.6

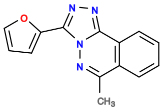

405D09

v>1.6 s=2.2

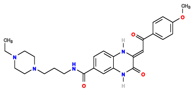

247E06

v>1.4 s=2.1

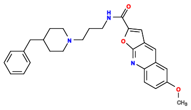

207H04

v>1.4 s=2.1

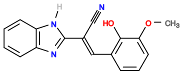

8F06

v>1.5 s=2.0

565872930 GCTGTC ACTG GAAAACGTAAA CAGT CTGAAGA GTCC AAATCTGCCGAGTTCGTGTGGGTGCGTG GGAC GAAACAGC

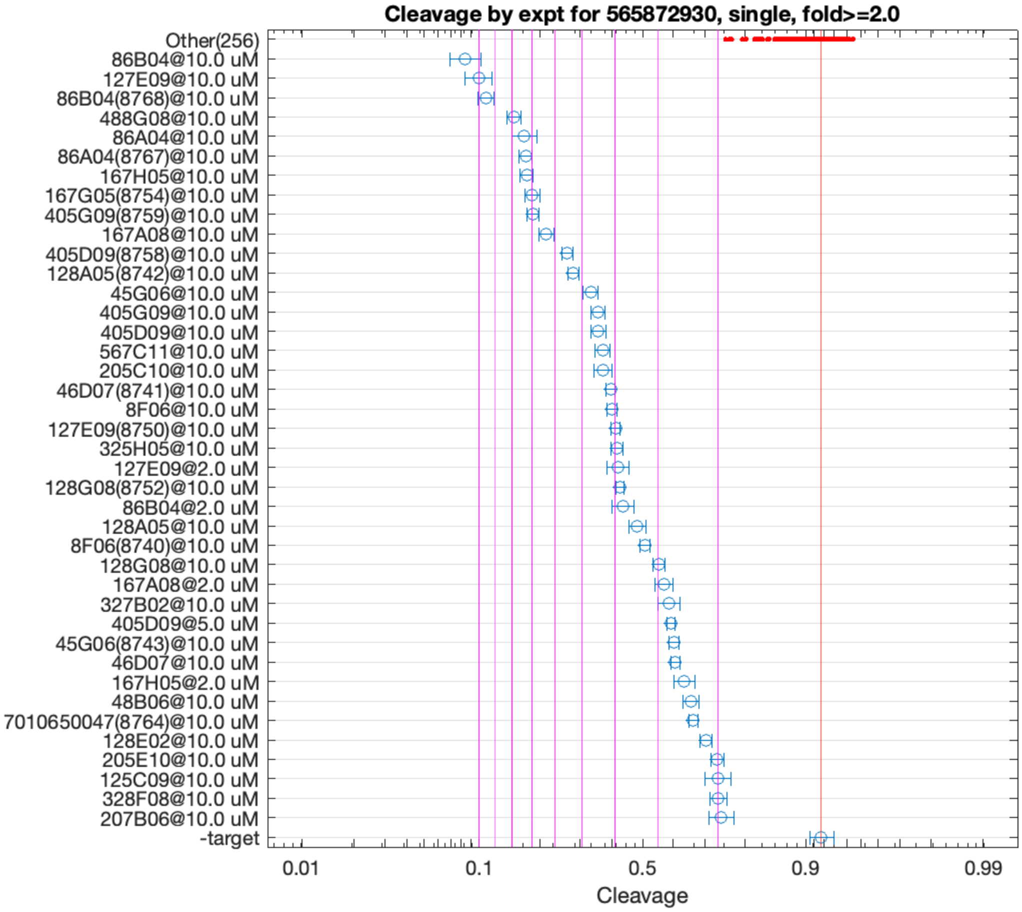

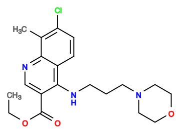

86B04

v>8.3 s=11.0

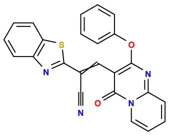

127E09

v>5.4 s=10.0

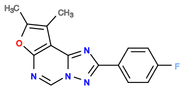

488G08

v>5.1 s=7.9

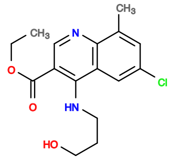

86A04

v>6.1 s=7.4

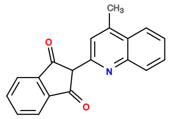

167H05

v>4.8 s=7.2

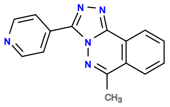

405G09

v>5.7 s=7.0

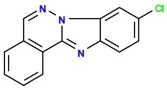

167G05

v>5.7 s=7.0

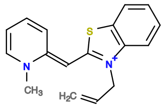

167A08

v>4.0 s=6.4

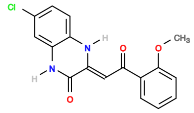

247C02

v>5.6

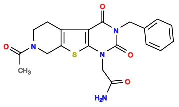

327A11

v>5.6

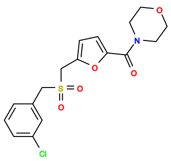

607A02

v>5.6

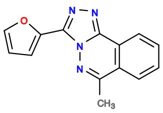

405D09

v>4.7 s=5.5

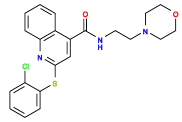

208H11

v>5.4

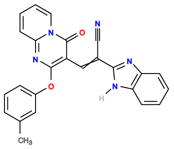

128A05

v>4.3 s=5.3

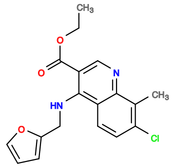

45G06

v>5.4 s=4.7

565928760 GCTGTC ACTGGA AAAAGGTTGCAATTCAAATGTCACTGGACTTC TCTGGT CTGATGA GTC AGAATGCTGACGAAACGGCTCTTGTTGTCGCG GAC GAAACAGC

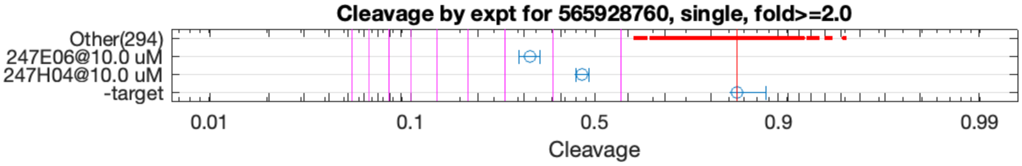

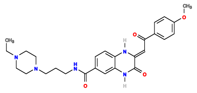

247E06

v>3.9 s=3.4

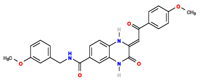

247H04

v>1.1 s=2.5

565957580 GCTGTC ACTGGAAT AAG GTTTCGGT CTGATGA GTCC GATGACGAAACAGCGAGTTGTGCGAACCCG GGAC GAAACAGC

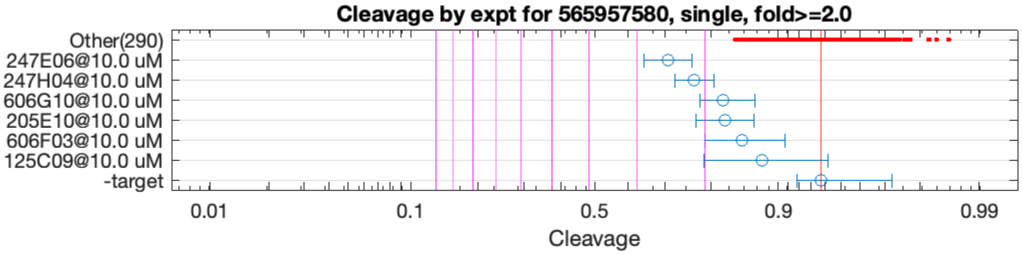

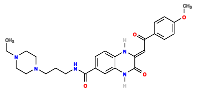

247E06

v>3.2 s=2.5

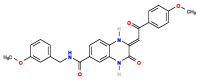

247H04

v>0.8 s=2.1

565958337 GCTGTC ACTGGA CACTACT TCCGGT CTGATGA GTCC GGGATACGACAAGTATGCCATTAGCAGGTG GGAC GAAACAGC

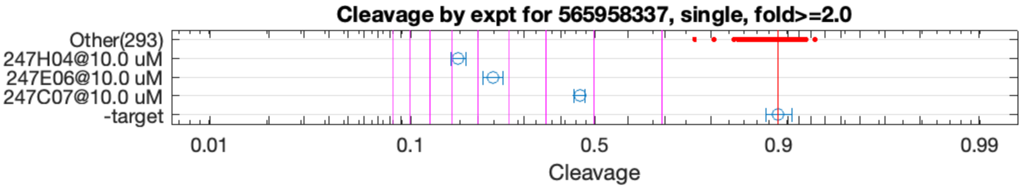

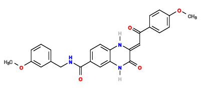

247H04

v>1.2 s=6.8

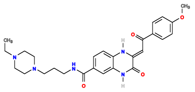

247E06

v>8.2 s=5.5

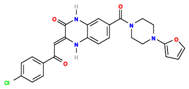

247C07

v>1.7 s=3.3

566071971 GCTGTC ACTGGAA CGTAGGTACTAGCGGAGTCAACACAATA TTCCGGT CTGACGA GTCCTG ATATCGCAAAGCTGGCACATACTACG TGGGAC GAAACAGC

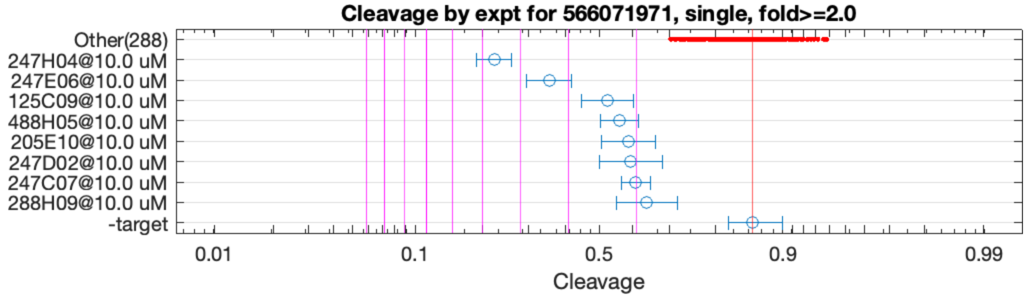

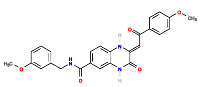

247H04

v>1.5 s=4.6

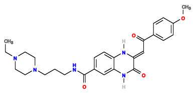

247E06

v>3.7 s=3.4

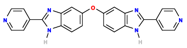

125C09

v>1.2 s=2.4

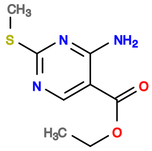

488H05

v>1.2 s=2.2

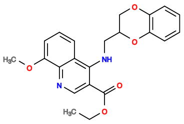

205E10

v>1.1 s=2.1

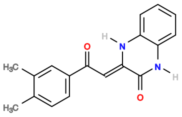

247D02

v>1.2 s=2.1

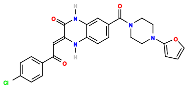

247C07

v>1.4 s=2.0

566087733 GCTGTC ACTGG ATTTATGCCGTTGAAAGGGGTGCCGATCTAAA CTAGT CTGAAGA GTCT GTGGCG GGAC GAAACAGC

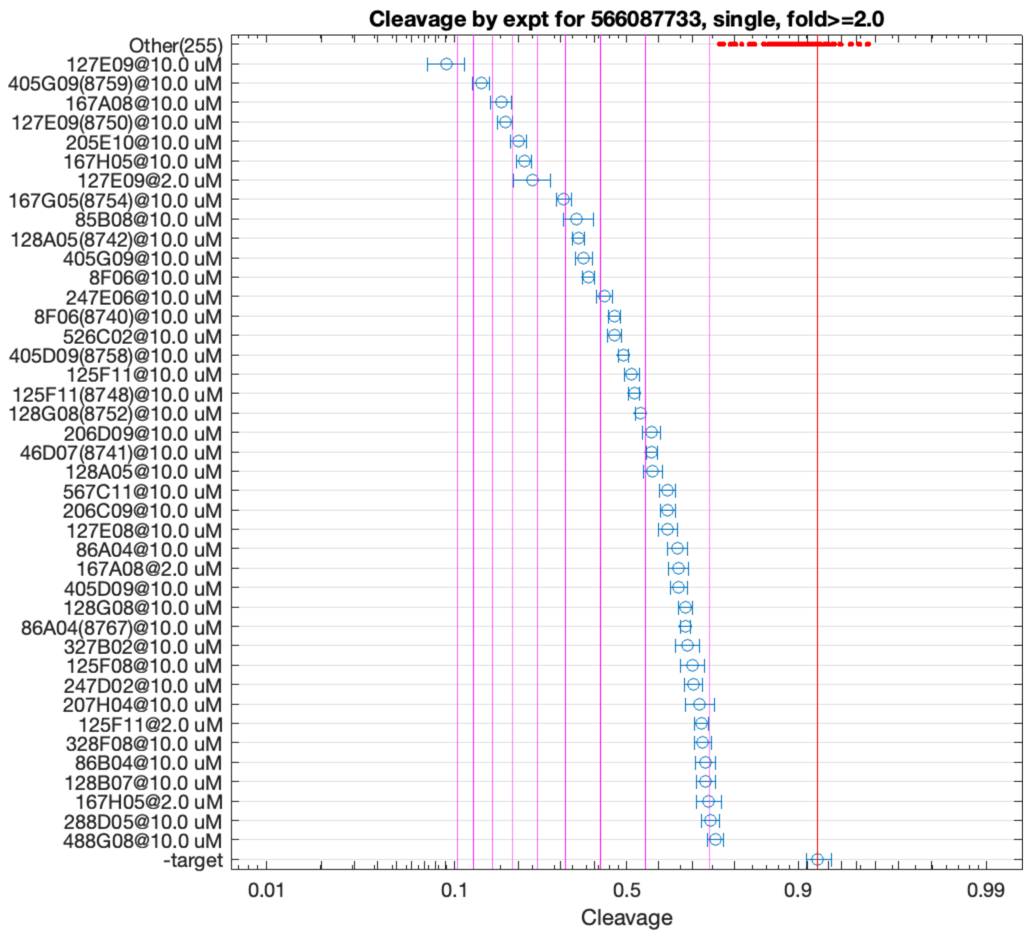

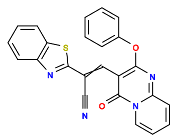

127E09

v>4.4 s=10.7

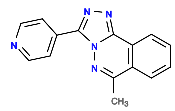

405G09

v>4.8 s=8.6

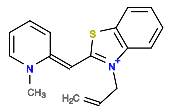

167A08

v>3.3 s=7.5

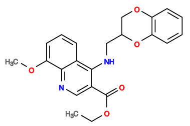

205E10

v>3.5 s=6.8

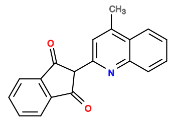

167H05

v>3.1 s=6.5

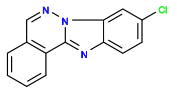

167G05

v>3.6 s=5.0

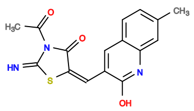

85B08

v>3.3 s=4.7

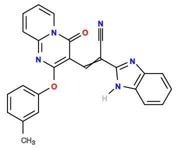

128A05

v>2.9 s=4.6

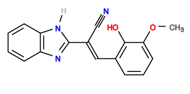

8F06

v>2.9 s=4.3

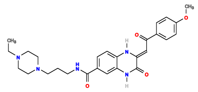

247E06

v>3.1 s=3.9

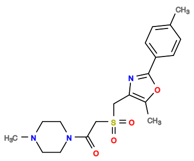

285A10

v>3.7

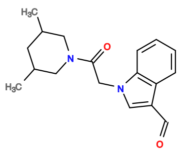

606E08

v>3.7

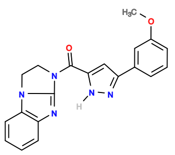

525D11

v>3.7

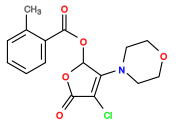

88E07

v>3.7

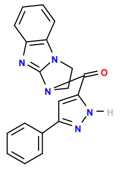

526C02

v>3.7 s=3.7

566087783 GCTGTC ACTGGA TTTTCCTGCAATGGTCTGTGGGGAAACGGC TCTGGT CTGATGA GTTC GTGGCG GGAC GAAACAGC

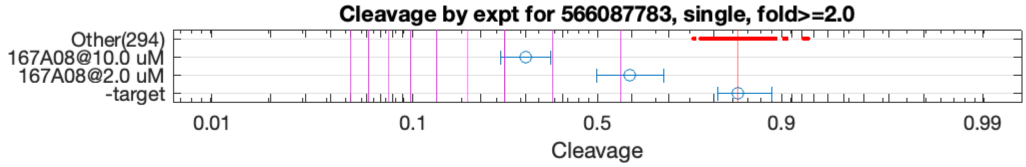

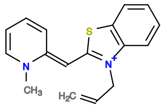

167A08

v>1.0 s=3.5

566218011 GCTGTC ACTGGA AGGGGACGA TCCGGT CTGATGA GTCC GTATCGAGGAAGAATTCGCGATATCCACAG GGAC GAAACAGC

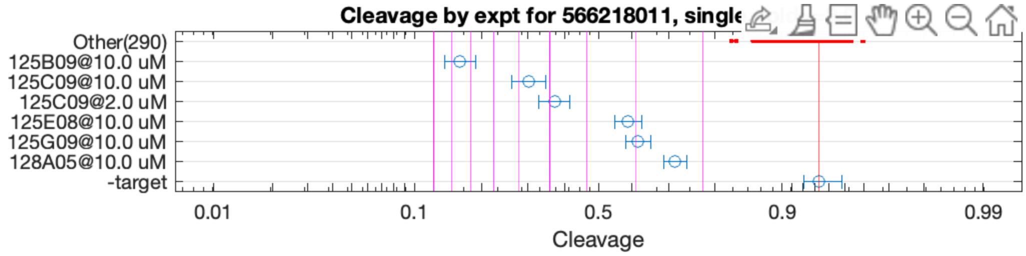

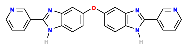

125B09

v>7.2 s=8.7

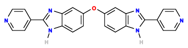

125C09

v>7.1 s=5.6

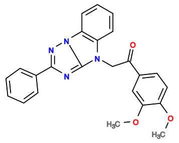

125E08

v>1.4 s=3.1

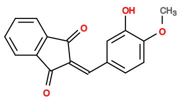

125G09

v>2.0 s=3.0

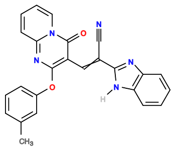

128A05

v>1.1 s=2.4

566222400 GCTGTC ACTGGA GGAAGATG TCCGGT CTGATGA GTCC GAAAATGTTCGCGTAGACAGGAGTTCGGTG GGAC GAAACAGC

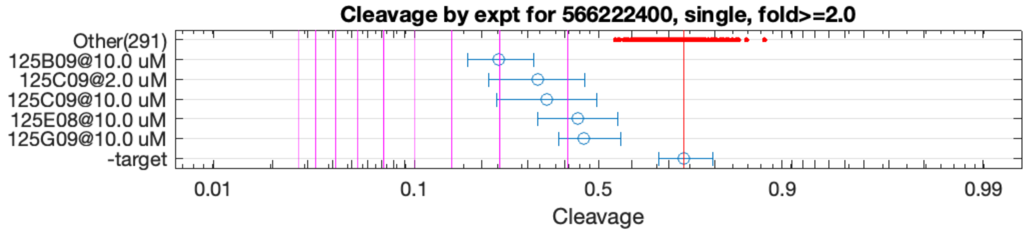

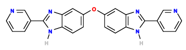

125B09

v>2.9 s=3.0

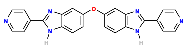

125C09

v>2.4 s=2.4

566225843 GCTGTC ACTGGA TTCTCTTTTGGTTTGCAAATGGGAACGGC TCCGGT CTGATGA GTTC GTGGCG GGAC GAAACAGC

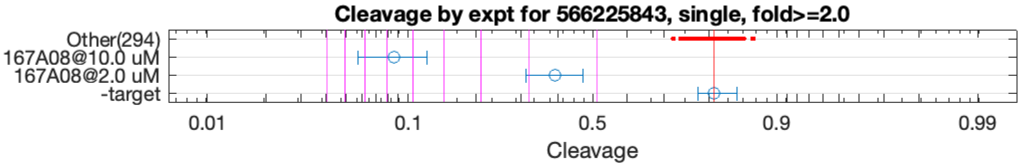

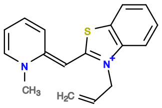

167A08

v>1.9 s=6.6

566226123 GCTGTC ACTGGA TTGTCTTTCGGAATTACTGGAAGATGACGGC TCCGGT CTGATGA GTTC GTGGCG GGAC GAAACAGC

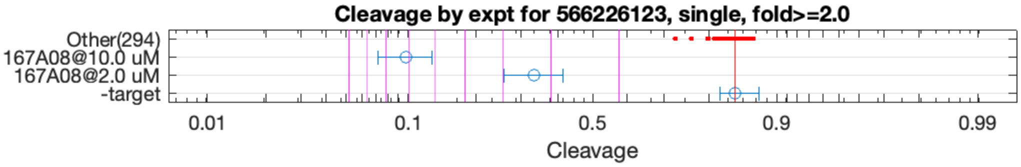

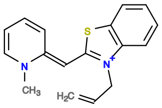

167A08

v>2.3 s=7.1

566228712 GCTGTC ACTGGA TTTCGGTTAGCAATGCTACTGGAACGGC TCCGGT CTGATGA GTTC GTGGCG GGAC GAAACAGC

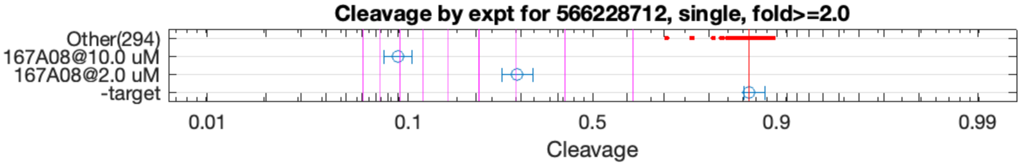

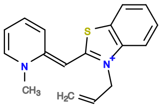

167A08

v>2.9 s=8.2

566230038 GCTGTC ACTGGA TTTCTTGTTGGTAGCAGCAGGAAACGGC TCCGGT CTGAAGA GTTC GTGGCG GGAC GAAACAGC

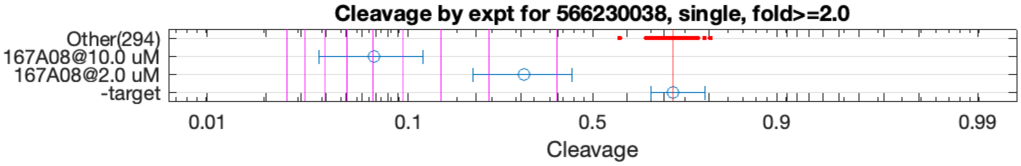

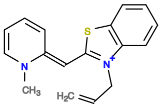

167A08

v>1.7 s=6.1

566264584 GCTGTC ACTGGA TATCACCGCAGAAATCAAGCCATTTCGAAC TCCGGT CTGATGA GTTC ACGTTCGGGATGAGCGCCTGGGGATGGGTA GGAC GAAACAGC

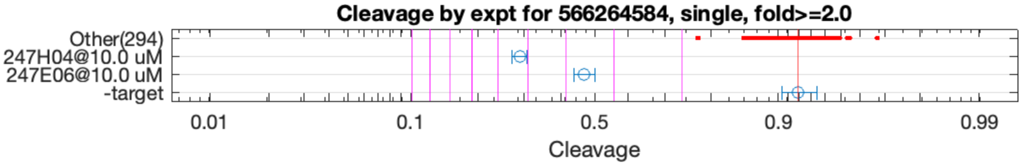

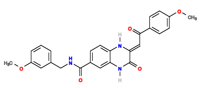

247H04

v>1.2 s=5.2

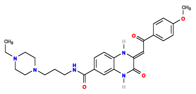

247E06

v>5.5 s=3.6

566297247 GCTGTC ACTGGA ATCCAAGCCCTATGAGCAACATGGTATACC TCCGGT CTGATGA GTTCT AGTCGCCATGTAGCACGGTAGCTGAGGA GGGAC GAAACAGC

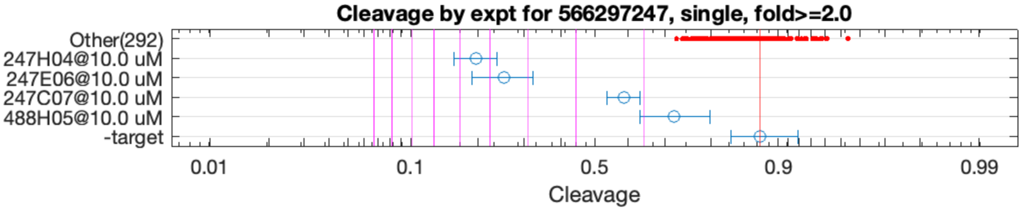

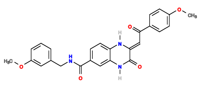

247H04

v>1.4 s=5.4

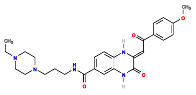

247E06

v>5.2 s=4.5

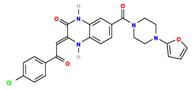

247C07

v>1.4 s=2.2

566304100 GCTGTC ACTGGAAT AAG GTTTCGGT CTGATGA GTCC GATGACGAAACAGCGAGTTGCGCGAACCCG GGAC GAAACAGC

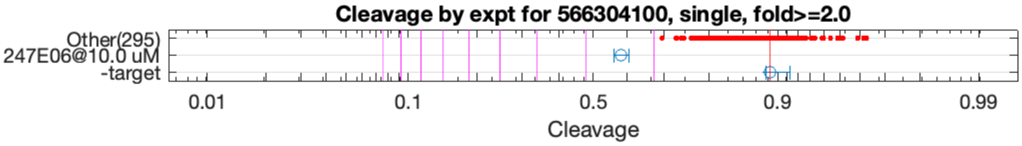

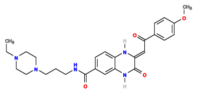

247E06

v>3.0 s=2.4

566314690 GCTGTC ACTGGAT GGT GTCCGGT CCGATGA GTCC GTGTCGAAAAAGTTGATTACTTCACACCCA GGAC GAAACAGC

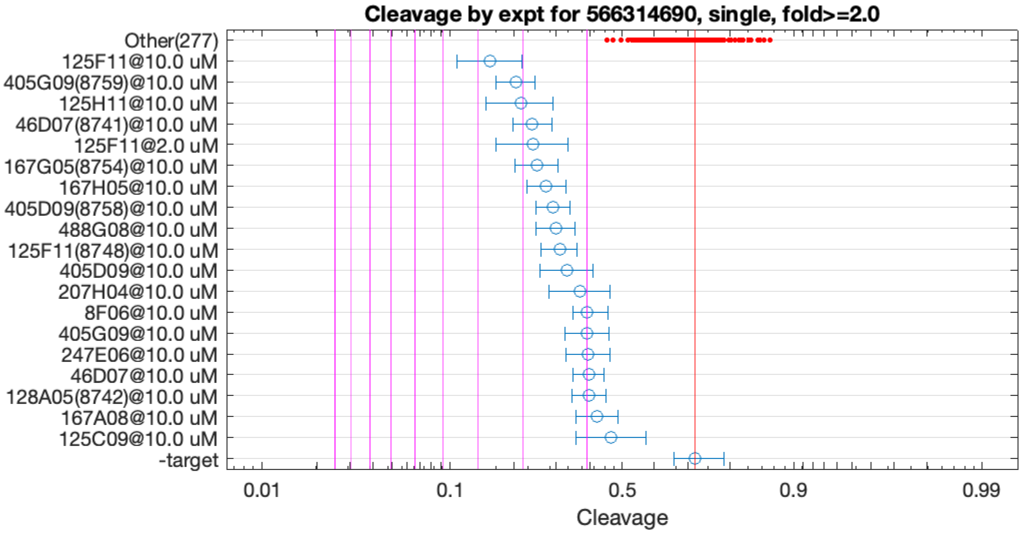

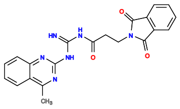

125F11

v>2.8 s=3.7

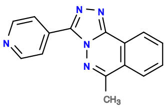

405G09

v>1.8 s=3.2

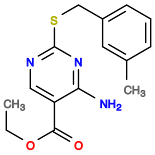

125H11

v>2.5 s=3.0

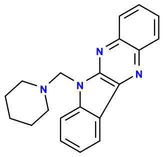

46D07

v>1.6 s=2.8

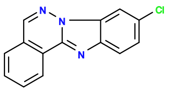

167G05

v>1.7 s=2.8

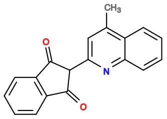

167H05

v>1.5 s=2.6

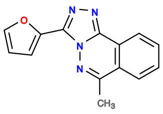

405D09

v>1.5 s=2.5

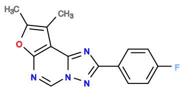

488G08

v>1.4 s=2.4

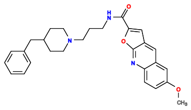

207H04

v>1.6 s=2.0

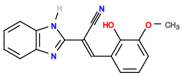

8F06

v>1.4 s=2.0

566334018 GCTGTC ACTGGA TTTTGCGCCGTTCTACTTGAACACT TCCGGT CCGATGA GTCCTT GCGC GGGGAC GAAACAGC

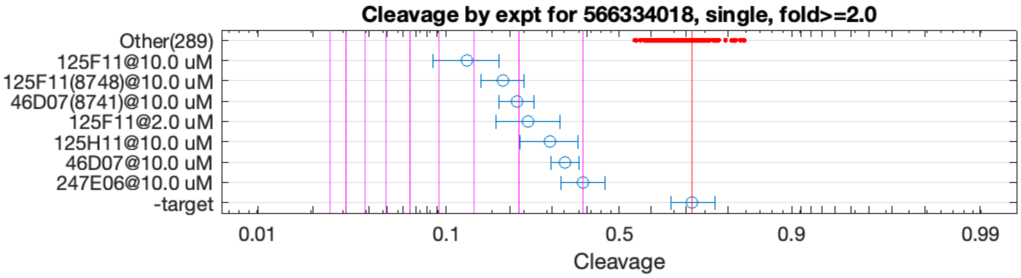

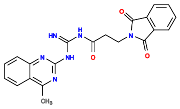

125F11

v>2.6 s=4.2

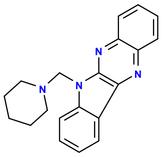

46D07

v>1.6 s=3.0

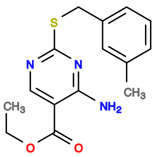

125H11

v>1.9 s=2.5

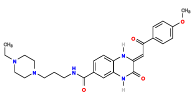

247E06

v>1.2 s=2.0

566566059 GCTGTC ACTGGAG CTGGACA CTCTGGT CTGATGA GTCC ATGAAGAGTGTGCGCAGACAAACGCACGGG GGAC GAAACAGC

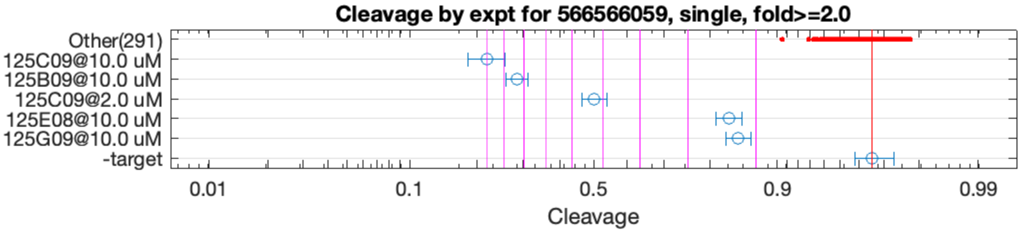

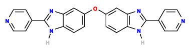

125C09

v>9.9 s=10.1

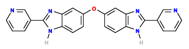

125B09

v>7.7 s=8.3

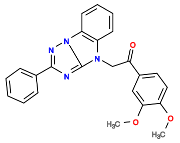

125E08

v>1.5 s=2.4

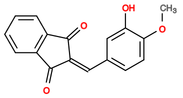

125G09

v>1.9 s=2.2

566568470 GCTGTC ACTGGAC GTGGCGA GTTCGGT CTGATGA GTCC AAAAGGAAGAGTGTGTGTGAGCACAGAGGC GGAC GAAACAGC

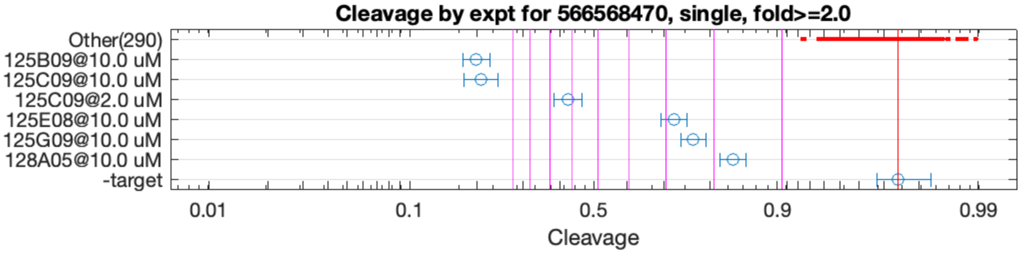

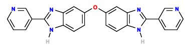

125B09

v>12.1 s=12.3

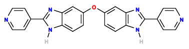

125C09

v>13.6 s=12.0

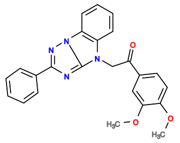

125E08

v>2.0 s=3.9

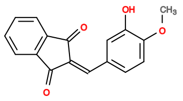

125G09

v>3.2 s=3.4

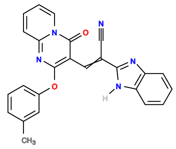

128A05

v>1.4 s=2.7

566652019 GCTGTC ACTGGA TACGATTTGGTGATTTCGCGCATGCCAAAC TCCGGT CTGACGA GTCT CAGTTTCGCATGCCCTTGCCAATGAAGTA GGAC GAAACAGC

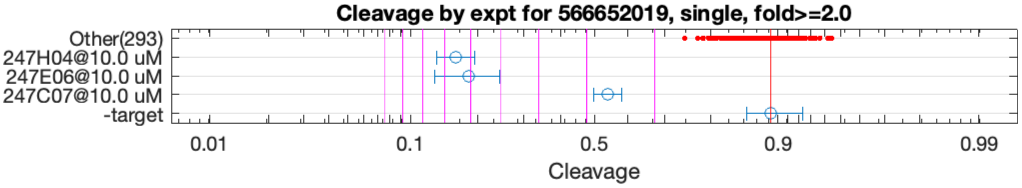

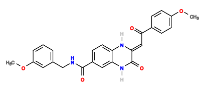

247H04

v>1.4 s=6.5

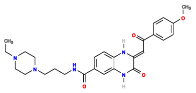

247E06

v>6.8 s=6.0

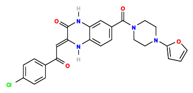

247C07

v>1.7 s=2.7

566655862 GCTGTC ACTGGAT GGT GTCCGGT CCGATGA GTCC GTGTCGAAAAGGTTGATTGCTTCACACCCA GGAC GAAACAGC

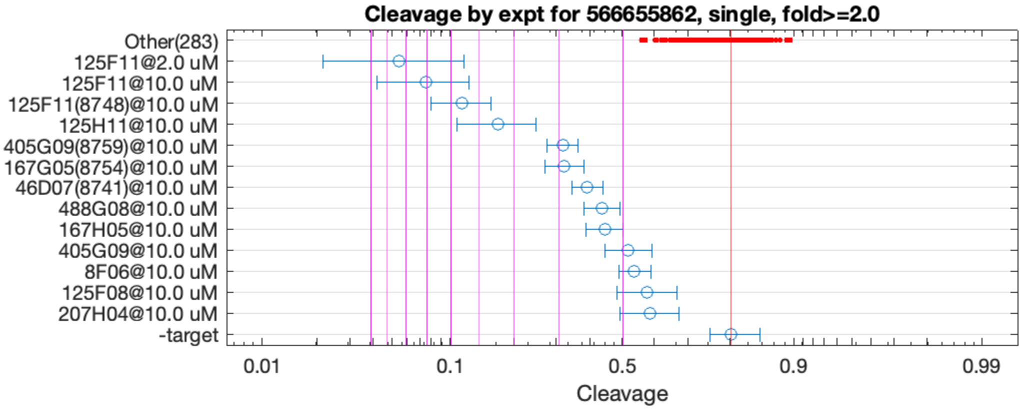

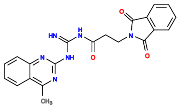

125F11

v>4.0 s=8.5

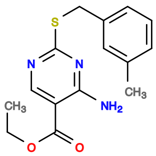

125H11

v>2.8 s=4.4

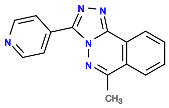

405G09

v>1.4 s=2.9

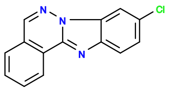

167G05

v>1.5 s=2.9

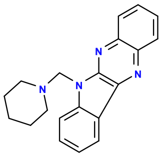

46D07

v>1.3 s=2.5

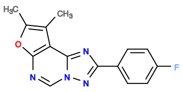

488G08

v>1.2 s=2.3

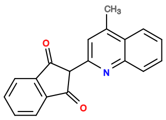

167H05

v>1.3 s=2.2

566669595 GCTGTC ACTGGA CACTCCGTGGGCATTTGGGTTGAATTGGCGAA TCCGGT CTGACGA GTCCTGTA GTCGGTTACCGTGCAGCTCAGG TGTGGGAC GAAACAGC

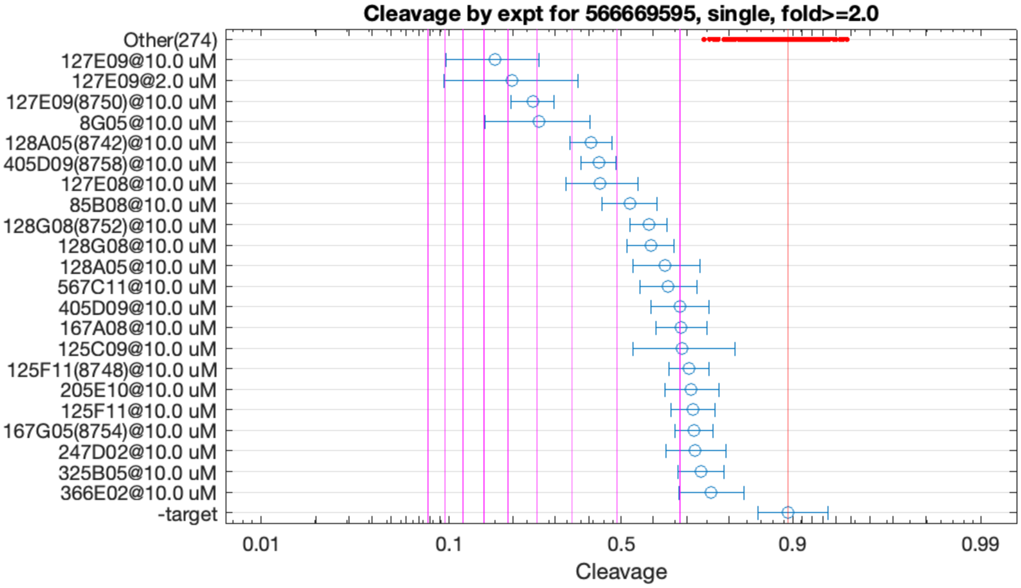

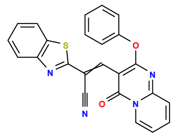

127E09

v>3.4 s=6.6

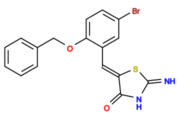

8G05

v>4.7 s=4.9

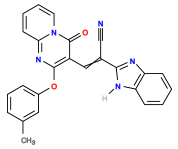

128A05

v>1.6 s=3.5

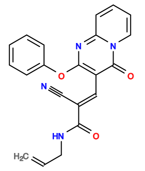

127E08

v>1.6 s=3.3

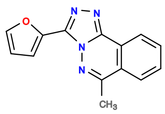

405D09

v>1.7 s=3.3

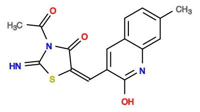

85B08

v>1.4 s=2.7

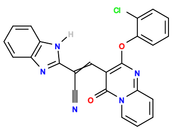

128G08

v>2.0 s=2.4

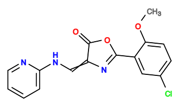

567C11

v>1.5 s=2.2

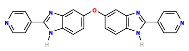

125C09

v>1.3 s=2.0
